# Endocannabinoid Signaling Regulates Sleep Stability

**DOI:** 10.1371/journal.pone.0152473

**Published:** 2016-03-31

**Authors:** Matthew J. Pava, Alexandros Makriyannis, David M. Lovinger

**Affiliations:** 1 Section on Synaptic Pharmacology, Laboratory for Integrative Neuroscience, Division of Intramural Biological and Clinical Research, National Institute on Alcohol Abuse and Alcoholism, National Institutes of Health, Rockville, MD, United States of America; 2 Center for Drug Discovery and Departments of Chemistry and Chemical Biology and Pharmaceutical Sciences, Northeastern University, Boston, Massachusetts, United States of America; Kent State University, UNITED STATES

## Abstract

The hypnogenic properties of cannabis have been recognized for centuries, but endogenous cannabinoid (endocannabinoid) regulation of vigilance states is poorly characterized. We report findings from a series of experiments in mice measuring sleep with polysomnography after various systemic pharmacological manipulations of the endocannabinoid system. Rapid, unbiased scoring of vigilance states was achieved using an automated algorithm that we devised and validated. Increasing endocannabinoid tone with a selective inhibitor of monoacyglycerol lipase (JZL184) or fatty acid amide hydrolase (AM3506) produced a transient increase in non-rapid eye movement (NREM) sleep due to an augmentation of the length of NREM bouts (NREM stability). Similarly, direct activation of type 1 cannabinoid (CB1) receptors with CP47,497 increased NREM stability, but both CP47,497 and JZL184 had a secondary effect that reduced NREM sleep time and stability. This secondary response to these drugs was similar to the early effect of CB1 blockade with the antagonist/inverse agonist AM281, which fragmented NREM sleep. The magnitude of the effects produced by JZL184 and AM281 were dependent on the time of day this drug was administered. While activation of CB1 resulted in only a slight reduction in gamma power, CB1 blockade had dramatic effects on broadband power in the EEG, particularly at low frequencies. However, CB1 blockade did not significantly reduce the rebound in NREM sleep following total sleep deprivation. These results support the hypothesis that endocannabinoid signaling through CB1 is necessary for NREM stability but it is not necessary for sleep homeostasis.

## Introduction

Since antiquity cannabinoids have been used as a treatment for insomnia [1], and the first reports in western medical literature regarding the therapeutic utility and physiological effects of cannabis preparations note their hypnogenic properties [2–5]. Additionally, this effect appears to be conserved across mammalian species [6–11]. Given the long standing recognition of cannabinoids as sleep promoting substances, it is surprising that relatively few studies have examined the role of the endogenous cannabinoid (endocannabinoid; eCB) system in regulating vigilance states.

Cannabinoids produce the majority of their central effects by activating the cannabinoid 1 receptor (CB1), and activation of this G-protein-coupled receptor (GPCR) reduces neurotransmitter release at many synapses [12]. CB1 is a central molecular component of the eCB system, an increasingly well characterized, lipid-based neuromodulatory system. The predominant transmitters for the eCB system are *N*-arachidonyl ethanolamide (anandamide; AEA) and 2-archidonylglycerol (2-AG). These molecules are released during periods of neuronal activity, and their inactivation occurs largely via distinct hydrolytic pathways. AEA is primarily inactivated via fatty acid amide hydrolase (FAAH), and 2-AG signaling is terminated by monoacyglycerol lipase (MAGL). Of the relatively few studies that have been performed, administration of exogenous AEA consistently increases rapid eye movement (REM) sleep and non-REM (NREM) sleep [13–16]. However, conflicting results arise from attempts to increase endogenous AEA levels. Some studies indicate that FAAH inhibition promotes wake [17, 18], but other reports show that blocking the AEA membrane transporter facilitates NREM sleep [19, 20]. Additionally, mice with a constitutive knockout of FAAH have increased NREM sleep time and reduced wake [21]. The effects of MAGL inhibition on sleep have not been examined.

While cannabinoids have been used by humans for many years to increase sleep, patients in clinical trials for the CB1 antagonist/inverse agonist, rimonabant, commonly reported sleep disturbances [22, 23]. In support of a sleep promoting role of eCB signaling, several studies have found fragmented sleep in CB1-null mutant mice [24, 25]. However, studies with constitutive knockout mice are always subject to confounds arising from developmental adaptations, and this has been confirmed for the CB1 knockout mice used in these studies [26, 27]. On the other hand, studies with CB1 antagonists in rodents have had conflicting results with some reporting a weak reduction in NREM sleep [15, 19, 28–30] and others finding no effects on sleep [13, 31, 32]. Of note, all of these studies were performed over short time windows (< 8 Hr recordings), and eCB levels are known to fluctuate over the circadian cycle [33, 34]. Thus, differences in the time of day these experiments were performed could explain some of this discrepancy.

As there is a poor consensus regarding the effects of eCB signaling on sleep, we performed a series of experiments comprising over 11,000 Hr of polysomnographic recordings in mice following a variety of pharmacological manipulations probing different aspects of the eCB system. To more fully account for the time course of effects, sleep measures were assessed over a 23.5 Hr period following all manipulations. To analyze this large volume of data, we developed and validated a novel automated state-scoring algorithm. In addition to a description of sleep, we also report results from power spectral analyses of electroencephalographic (EEG) recordings. Finally, we directly tested whether eCB signaling is necessary for homeostatic regulation of sleep by blocking CB1 signaling during recovery from total sleep deprivation (TSD). Our findings indicate that eCB signaling is both necessary and sufficient to promote long (stable) bouts of NREM sleep, but eCBs are not necessary for sleep homeostasis. These findings constitute a thorough characterization of eCB modulation of vigilance states that should provide a platform for future studies examining the physiological mechanisms of eCB regulation of sleep.

## Methods

### Ethics Statement

This research involved the use of vertebrate animals (mice), including survival surgical procedures to implant electrodes. All methods were approved by the Institutional Animal Care and Use Committee of the National Institute on Alcohol Abuse and Alcoholism (protocol #: LIN-DL-22) and hewed to guidelines specified in the Guide to the Care and Use of Laboratory Animals [35]. Survival surgeries were performed under isoflurane anesthesia, and ketoprofen (5 mg/kg i.p.) analgesic solution was administered immediately after surgery and every 24 hr for the next two days.

### Subjects

Male C57BL/6J mice (103, 8–10 week olds) were obtained from the Jackson Laboratory (Bar Harbor, ME), and initially group housed, 2–4 mice per cage. Mice weighed 25–30 g at the beginning of the study, and body weight did not change substantially over the course of experiments. Following surgery, subjects were single housed for the remainder of the study. At all times, subjects were provided with *ad libitum* food and water. The colony and sleep recording environment were maintained on a 12 hr light:dark cycle with the light photoperiod (LP) starting at 06:30 and the dark photoperiod (DP) beginning at 18:30. For the experiment where JZL 184 was administered prior to the LP, mice were housed in reverse cycle conditions with lights turning on at 18:30 and off at 06:30 for 2 weeks prior to recordings and throughout the recording period. Time of day is expressed throughout this manuscript relative to the light zeitgeber (ZT) with ZT 00:00 coinciding with beginning of LP and ZT 12:00 coinciding with the beginning of the DP. The colony and recording environment were maintained at 22.2°C and 50% humidity.

### Surgical Implantation of Electrodes

Prior to surgery, custom implants were prepared. One end of three single-stranded, Teflon coated stainless steel wires (#791500, A-M Systems, Sequim, WA) was soldered to individual gold-plated sockets (E363/0, PlasticsOne, Roanoke, VA). These three gold sockets and the socket attached to a stainless steel suture pad (E363T/2, PlasticsOne) were arranged in a plastic 6 channel connector (MS363, PlasticsOne) and secured with non-conductive epoxy. During surgery, two of the stainless steel wires emerging from the implant were wrapped, separately, around the frontal electrodes to provide two EEG channels. The ground electrodes were shorted together with the remaining wire. To ensure electrical connectivity with the EEG and ground electrodes a small amount of electrically conductive glue (Bare Paint, Bare Conductive Ltd., London, UK) was applied at the junction between wires and the stainless steel screws.

Stereotaxic surgery was performed to implant subjects with EEG/EMG electrodes. EEG electrodes consisting of stainless steel screws (Small Parts# AMS90/1P-25, Amazon Supply, Seattle, WA) were implanted supradurally through the skull. Two electrodes were implanted over frontal cortex (B: RC +2.64, ML ± 1.38) and referenced to two, connected ground electrodes implanted over occipital cortex (B: RC—2.5, ML ± 2). The EMG electrode (metal suture pad, PlasticsOne, Roanoke, VA) was implanted underneath the nuchal muscle. A head cap was formed with standard, cold-cure dental acrylic, and subjects were allowed to recuperate for two weeks in their home cages.

### Sleep Recordings

Following recuperation from surgery, subjects were lightly anesthetized with isoflurane and connected to a non-motorized commutator (SL6C/SB, Plastics One) via an electrical tether. Subjects were placed into a recording home cage fabricated from a 4 liter, clear polycarbonate bucket (Cambro RFSCW4135, Webstaurant Store, Lancaster, PA). These cages contained standard corncob bedding, and food pellets were placed on the cage floor. Access to water was provided via glass liquid diet feeding tubes (#9019, Bio-serve, Frenchtown, NJ) inserted through a hole drilled through the side of each cage. The commutators were secured to a hole in the cage lid thus ensuring that mice did not become entangled in their tethers. Five cages were placed inside sound and light attenuating chambers equipped with a fan and white LED light strips (# 10434, General Electric, Fairfield, CT). The lights were on a timer synchronized with the colony lights and the data acquisition PC clock. Additionally, the inside of the chamber was lined with either copper mesh or coated in conductive paint to shield the inside from electromagnetic interference.

Prior to recording, mice were habituated to this environment for 7 days. On recording days, data were collected over a 23.5 Hr period. The time at which recordings were initiated depended on the experimental protocol, but this was generally either before the onset of the LP (ZT 00:00) or before the onset of the DP (ZT 12:00). For the sleep deprivation experiment, data collection was initiated in the middle of the LP (ZT 06:00). Individual cages were removed from the recording chambers during the 30 min between recording sessions, and subjects were weighed and administered an i.p. injection of saline, vehicle, or a drug. After injections, subjects were placed back into their respective cages, and these were returned to the recording chambers. In studies with multiple doses of drugs, we explicitly chose to use a schedule of escalating doses over a series of days. Cannabinoids can rapidly induce tolerance, and thus, if a counter-balanced design was implemented, any effect of low-dosage on sleep parameters would likely be prevented by the preceding administration of a high dose. Additionally, compensatory effects were observed following high dose administration of cannabinoids (for example, see results from the recovery day in experiments with JZL184 and AM3506), so a counterbalanced design on successive days could lead to erroneous conclusions that low dosage administration promotes effects opposing those of high-doses.

EEG and EMG signals were amplified 1000x (20x HST/16V-G20 headstage followed by 50x wide-band PBX, Plexon, Dallas, TX). The amplified signals were digitized using a National Instruments digitizer (PCI-6071E, National Instruments, Austin, TX) connected to a standard PC computer (Optiplex GX620, Dell Computers, Round Rock, TX) running Recorder v2 (Plexon). Data were visualized as they were being collected using Recorder software’s built-in oscilloscope, and in cases where signals were observed to be of poor quality (e.g. low signal-to-noise ratio, low dynamic range), the subject was not used for analysis of sleep or drug-induced changes in EEG spectral power. This was almost always the result of a faulty EMG electrode. As an additional check to ensure subjects with corrupt (unscorable) signals were not included in analysis, subsequent visual inspection of the scoring results (including raw EEG/EMG signals) for each subject alerted the investigator to any problems of low signal quality that could arise at a point in the experiment when online data collection was not being monitored. All data were sampled at 1 kHz, and a 60 Hz notch filter was applied to eliminate line noise. EEG signals were low-pass filtered online at 120 Hz with a 2 pole Bessel filter, and EMG signals were high pass filtered at 40 Hz with a 4 pole Bessel filter. Data were saved for offline analysis.

### Sleep Deprivation

The custom sleep deprivation apparatus (S1 Fig, panel A) used in this report was constructed from a 10 in long clear acrylic tube with an internal diameter of 5 in that was suspended slightly above an epoxy-sealed ABS disc that served as the chamber floor. The disc and other structural components of the apparatus were custom designed in CAD software (Sketchup 8, Trimble Navigation Ltd, Sunnyvale, CA) and constructed from 3D printed ABS plastic (3D XL printer, Airwolf3D, Costa Mesa, CA). The cylinder had an acrylic divider that extended the length of its radius from the interior wall directly into the center of the chamber (S1 Fig, panel B). This divider ensured the animal would move when the floor of the device rotated. The chambers contained standard corncob bedding, and a sufficient amount of standard mouse chow was placed on the floor to provide ad libitum access to food. A glass liquid diet feeding tube provided ad libitum access to water. The bottom of ABS disc was attached to a 360 degree servo motor (DF15RSMG, DFRobot, Shanghai, China), and the motors of five chambers were controlled by an Arduino UNO board (Adafruit Industries, New York, NY) that received commands via a serial connection with a laptop running MATLAB (S1 Fig, panel C). The signal from the Arduino board and the servos were calibrated to rotate at approximately 15 rpm. Rotation was engaged for a random time interval between 10–15 sec and was turned off for a random interval between 5–10 sec. Additionally, the rotational direction of the disc was randomly alternated to prevent habituation to movement in one direction. In preliminary studies, we found this intermittent schedule to be effective in producing near total sleep deprivation (< 1% of time spent in NREM sleep) for up to 6 Hr in most subjects. Importantly, the devices were constructed so that a commutator (SL6C/SB, Plastics One) could be connected to the chamber lid. This allowed continuous recording of the EEG and EMG signals while the subject was housed in the apparatus.

Sleep deprivation experiments consisted of three phases: baseline, deprivation, and recovery. Polysomnographic measures of sleep were obtained across all phases of the experiment. Following habituation to the standard recording environment, 48 Hr baseline recordings were obtained from all subjects following an i.p. injection of vehicle (1:1:18 mixture DMSO: Cremaphor: 0.9% Saline) at ZT 06:00. After the baseline recordings, subjects were transferred into the sleep deprivation devices (S1 Fig). Importantly, their original recording cages (including bedding, food, and water bottles) were retained and labelled according to subject. TSD via forced locomotion was initiated at the onset of the LP (ZT 00:00), 18 Hr after the subjects were placed into the deprivation device. The TSD protocol continued for 6 Hr, and afterwards subjects were immediately removed from the deprivation chambers, weighed, and received either an i.p. injection of the vehicle solution (control) or 5 mg/kg AM281. Subjects were then returned to their original recording cage for 48 Hr (recovery phase). TSD was defined as spending less than 1% of total time (< 3.6 minutes) in NREM sleep, and based on this criterion, we disqualified 5 out of 14 mice in the AM281 group and 3 out of 14 mice in the vehicle group.

### Vigilance State Scoring

**Fig 1:**
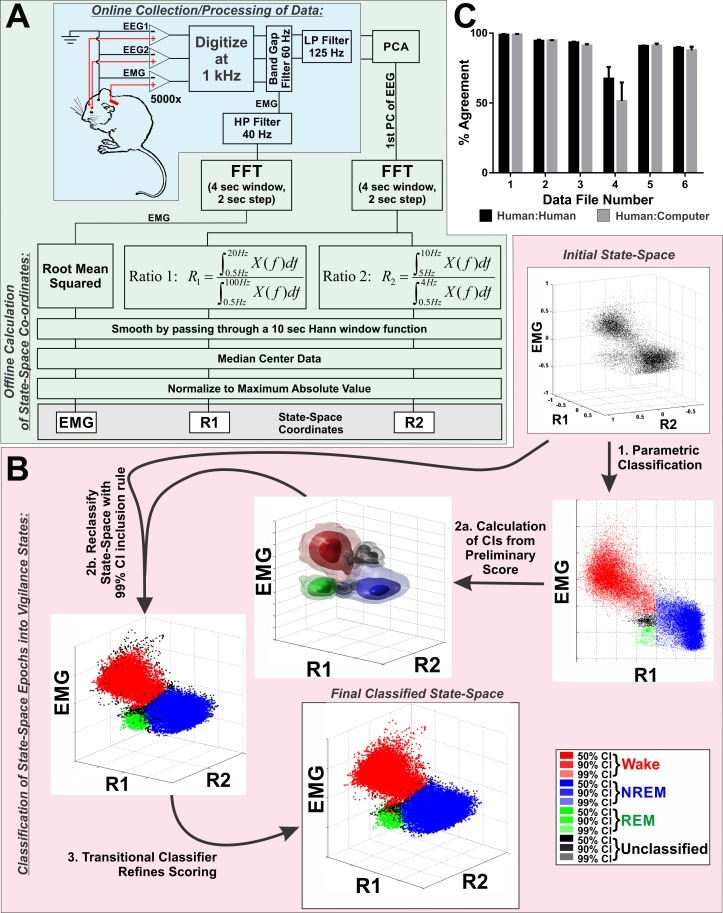
Design and Validation of Fully Automated Vigilance State Scoring Algorithm. **A,** Schematic diagram of data processing during acquisition (blue shaded region) and during offline calculation of 3-dimensional state-space coordinates (green shaded region). The grey box at the end of the flow chart highlights the three coordinates that define the state space. At this stage, no state-assignment has been made. Each point in the final state-space represents a 2 sec epoch. **B,** Schematic illustrating the three-step, automated process for classifying points in the state-space (pink shaded region) into either wake, rapid eye movement (REM), or non-rapid eye movement (NREM) sleep. Additionally, points that are ambiguously positioned on the boundary between clusters can be defined as unclassified. *Step 1*: parametric classification establishes regions of the state-space consistent with the three vigilance states based on hard cutoff criteria determined from the distribution of points within the state-space. *Step 2a*: from the classification performed in step 1, 99% confidence intervals (CIs) are constructed for each state using a product kernel estimator with a Gaussian kernel function and Scott’s Rule for bandwidth determination. *Step 2b*: The state-space is reclassified using a simple inclusion rule with the 99% CIs constructed in step 2a. *Step 3*: A transitional classifier is used to incorporate most points that were outside the 99% CIs into a state-classification. All strings of unclassified points that are bounded on either side by epochs of the same state are incorporated into that state classification (e.g. wake–unclassified–unclassified–wake becomes wake–wake–wake–wake). **C,** Validation results comparing the percent agreement between three trained human scorers (inter-rater reliability) with percent agreement between each human and the computer assigned scores. Bars represent mean±SEM. Abbreviations: CI–confidence interval, EEG–electroencephalogram, EMG–electromyogram (A) or state-space coordinate derived from electromyogram signal (B), FFT–Fast Fourier Transform, HP–high pass, LP–low pass, PC–principle component, PCA–principle component analysis, R1 –ratio 1, R2 –ratio 2.

To obtain an unbiased estimate of sleep-wake states, we devised an automated algorithm to score polysomnographic data as either wake, NREM, or REM sleep (Fig 1A and 1B). Importantly, this software arrives at a deterministic score of a 24 Hr single-subject recording in less than 5 min, assigning scores to 2 sec epochs, and it performs as well as trained human scorers (Fig 1C and S3 Fig).

#### Calculation of the State-Space

The first step, deriving the state-space (Fig 1A), was heavily influenced by the state-space methodology reported by Gervasoni et al [36], but in addition to electrographic signals from the brain, we also incorporated EMG activity to conform with standard polysomnography techniques used in mice [37]. However, our methodology differed somewhat from other state-space based approaches. Because we were recording from two EEG channels, we first compressed this data by taking the first principle component (PC) of the raw data. By only performing analysis on the 1^st^ PC of the two frontal EEG signals, computational overhead is reduced. Next, power spectra were obtained for the EEG and EMG waveforms using a 4 sec sliding window FFT with a 2 sec step. This was implemented using the spectrogram() function that is part of the signal processing toolbox in MATLAB (The Mathworks Inc, Natick, MA). This provided frequency domain data in 2 sec epochs with roughly 0.25 Hz bin resolution. Two power spectral ratios (R1 & R2) were calculated from the EEG data for each epoch:

$$\text{Ratio}\;1=\left[{R1}_{i}\right]=\frac{\sum_{j=0.5\;Hz}^{20\;Hz}\;P\left(f_{j}\right)}{\sum_{j=0.5\;Hz}^{100\;Hz}\;P(f_{j})}\;\text{Ratio}\;2=\left[{R2}_{i}\right]=\frac{\sum_{j=5\;Hz}^{10\;Hz}\;P(f_{j})}{\sum_{j=0.5\;Hz}^{4\;Hz}\;P(f_{j})}$$

Ratio 1 (R1) provides good separation between NREM and wake epochs [36, 38]. However, separating REM from the other two clusters is challenging due to the sparse nature of REM sleep and the similarity of the power spectra between REM and wake in mice. However, theta rhythms (5–8 Hz) are prominent during REM sleep, and this characteristic has been used as a means to separate REM epochs into a distinct, if somewhat diffuse cluster [38–40]. Thus, we defined Ratio 2 (R2) to help pull epochs with high theta power away from the major clusters representing NREM and wake. In addition to prominent theta, REM sleep is also distinguished from wake by significantly reduced muscle tonus which can be measured with EMG. Therefore, we incorporated this criterion into the state-space by including the RMS value of the power spectra of the EMG waveform. In summary, three criteria were used to separate epochs of polysomnographic data into a 3D state-space: (1) Prominence of the frequencies between 0.5 and 20 Hz relative to the entire power spectra (0.5–100 Hz), (2) Prominence of theta power relative to delta (0.5-4Hz), (3) RMS of the power spectra of the EMG waveform.

To provide better cluster separation, the state-space coordinates are smoothed with a 10 sec Hann function and log transformed. To standardize the range of axes occupied by the state-space the log transformed data were median centered and normalized to the max absolute value on each axis. This bounds the state-space between -1 and +1 across all axes for all subjects. The first graph in Fig 1B provides an example of the state-space at this stage of algorithm.

#### Classification of Points in State-Space as Vigilance States

Once the state-space is computed, three distinct clusters can be observed, and the second step of the classification software (Fig 1B) serves to automatically define these clusters. This process occurs over three sequential steps, where a rough description of clusters based on hard cutoffs is used to establish statistical boundaries (confidence intervals) that reclassify the data using an inclusion/exclusion test. In the final step, the remaining epochs that are not assigned to wake, NREM, or REM states are restricted to epochs that are ambiguous to classify because they occur during transitions between vigilance states.

First, a rough estimate of cluster boundaries is obtained by establishing threshold values based on the distribution of points along each axis **(Fig 1B, step 1**). Specifically, a univariate kernel density estimate is performed independently for EMG and R1. The distribution along R1 is bimodal, so the threshold separating wake and REM from NREM along the R1 axis is defined as the local minimum between these modes. The distribution of EMG values is not consistently bimodal, so NREM epochs are constrained by the third quartile along this axis. Similarly, wake was defined as epochs on the opposite side of the R1 distribution threshold with EMG values greater than the median. REM was defined as values in the same mode of R1 as wake with EMG values within the first tercile. Additionally, REM was constrained to only those epochs above the median value on the R2 axis. The purpose of the these hard-cut criteria is to seed clusters based on the consistent topology of the state-space. The next two steps of the classification process substantially refine this initial classification. The values for thresholds were visually determined by the experimenter after trial and error with many data sets as reasonable thresholds to capture the majority of each cluster across the overwhelming majority of datasets tested. Importantly, not all points are classified in this step (see Fig 1B, step 1), and there is a buffer of unclassified points left between wake and REM that is subsequently incorporated into these clusters after completing steps 2 & 3 of the classification process. After inspecting the classified state-space for each data file, there were some rare instances where it was clear that the REM cluster was not adequately defined by this step of the algorithm, and in these instances, a custom software routine allowed for the manual selection of the REM cluster.

Based upon this initial classification, confidence intervals were calculated for each cluster by estimating the probability density function (PDF) for each cluster separately (${\hat{f}}_{State}$) and the whole state-space (${\hat{f}}_{total}$), using kernel estimation with a Gaussian kernel:

$${\hat{f}}_{subset}\left(x\right)={\left(nh_{1}h_{2}h_{3}\right)}^{-1}\sum_{i=1}^{n}\left\{\prod_{j=1}^{3}K\left(\frac{x_{j}-X_{ij}}{h_{j}}\right)\right\},\;K\left(t\right)=\frac{1}{\sqrt{2\pi }}e^{-t^{2}/2}$$

The smoothing parameter was determined based on the dataset using Scott’s Rule:

$${\hat{h}}_{i}={\hat{\sigma }}_{i}n^{-1/7}$$

Because some points were not assigned a state in the first classification step, there was a separate PDF calculated for these points in addition to the estimates for wake, NREM, and REM. After all kernel estimates had been obtained, they were scaled so the maximum value of each component PDF was equal to the corresponding grid location in ${\hat{f}}_{total}$. In this way, the PDF of the entire state-space was decomposed into component densities representing the different states (Fig 1B, step 2a).

$${\hat{f}}_{total}={\hat{f}}_{wake}+{\hat{f}}_{NREM}+{\hat{f}}_{REM}+{\hat{f}}_{Unassigned}$$

To determine the probability that a given point belonged to a specified cluster, the component PDFs were subtracted from one another and normalized to the absolute value of the resulting maxima:

$$P\left(A|x\right)=\frac{{\hat{f}}_{A}-{\hat{f}}_{B}-{\hat{f}}_{C}-{\hat{f}}_{D}}{\left|\text{max}\left({\hat{f}}_{A}-{\hat{f}}_{B}-{\hat{f}}_{C}-{\hat{f}}_{D}\right)\right|}$$

Where A, B, C, and D represent different states (Wake, NREM, REM, and Unassigned), and ***x*** is a three dimensional feature vector for a specified epoch of the state-space. This subtraction and normalization step was performed for each component density yielding four probability matrices. The subtraction step was important to delineate clean borders between states.

At this stage of processing, points in the state-space were reclassified using the probability matrices defined above. To accomplish this, each epoch of the state-space was indexed into the four probability matrices, to determine the probability that it belonged to each state. The epoch was assigned to the state with the highest probability if it fell within confidence intervals specified *a priori*. We established 99.9% confidence intervals for all states. Points that fell outside of these confidence intervals were assigned to the unclassified cluster, and similarly, points that had equivalent probability of belonging to two or more clusters were assigned to the unclassified cluster (Fig 1B, step 2b).

As can be seen in the results following classification with 99.9% confidence intervals (Fig 1B, step 2b), unclassified epochs comprised points on the periphery of clusters and transitional epochs between clusters. To further refine the state assignment, a final classification step was performed using a transitional classifier (Fig 1B, step 3). The point of this last step was to reduce unclassified epochs to only those epochs representing transitions between states where state scoring is inherently ambiguous. Consequently, this classification step assigned all unclassified epochs bounded by an epoch of the same state, while unclassified epochs bounded by different states would remain unclassified. Thus, the sequence [wake, unclassified, unclassified, wake] would become [wake, wake, wake, wake], while [wake, unclassified, unclassified, NREM] would remain the same. As shown in the last graph of Fig 1B, the result of this classification step was to eliminate the penumbra of unclassified epochs surrounding the clusters, while leaving the unclassified epochs between cluster boundaries unchanged.

Most of this analysis was coded in MATLAB and C/C++ (MEX file libraries). However, a parallelized kernel density estimation method, GPUML, was implemented in CUDA/C (NVIDIA Corp, Santa Clara, CA) to speed computation of non-parametric density estimation [41]. Throughout the algorithm, operations were parallelized where possible, and this was achieved via explicit coding of parfor and spmd loops using MATLAB’s Parallel Computing Toolbox. Importantly, for each data set (all subjects * days of experiment), the scoring results were visually examined to ensure there were no obvious defects in scoring.

The prevalence of each state was calculated as the percent of time spent in that state. Additionally, the number and duration of bouts of NREM and REM were calculated (sleep architecture) with one bout defined as a consecutive series of epochs in the same state.

### Drugs

CP47,497 (CP47), AM281, JZL184 (JZL), and URB597 (URB) were all obtained from Tocris Bioscience (Bristol, UK). These compounds are highly lipophilic and only sparingly soluble in aqueous solution. Therefore, these drugs were dissolved in a vehicle solution consisting of a 1:1:18 mixture of DMSO, Cremaphor, and normal saline. Administration of this vehicle had no effect on either sleep or EEG power spectra (S2 Fig). AM3506 was synthesized in the laboratory of Dr. Alexandros Makriyannis (Northeastern University), and was prepared in a 1:1:8 vehicle of DMSO:Cremaphor:Saline because it tends to precipitate when prepared in the standard vehicle solution. For all experiments, 24 Hr recordings of polysomnographic indices following administration of the appropriate vehicle solution were used as a within-subject baseline for comparison. All drug and vehicle solutions were administered via i.p. injections given at a volume of 0.02 mL per gram body weight. Drug were prepared fresh on the day of the experiment.

### Statistics

In all experiments, time of day was included as a factor, but it was necessary to take into account the effect of photoperiod as well. Therefore, all analyses of time course data utilized a hierarchical linear mixed model (HLM) approach. For most experiments, a model with three repeated, fixed factors was implemented. Specifically, the model tested for the interaction between drug treatment and time of day nested within photoperiod. Because we followed subjects across different treatment conditions, all analyses contained repeated measures, and post-hoc comparisons were performed within-subjects. For the sleep deprivation experiment, there were two experimental groups (AM281 treated and vehicle control). For simplicity, only data from the first day following baseline vehicle injection (baseline day 1) and the first day following sleep deprivation (recovery day 1) were statistically compared. In this case, the model design examined the between groups interaction of treatment group (vehicle vs AM281) with time of day nested within photoperiod (light vs dark) nested within experimental phase (baseline vs recovery), where time of day, photoperiod, and experimental phase were all repeated measures. Analysis with HLM was performed in SPSS (IBM, Bethesda, MD). A Bonferroni correction was applied to all pair-wise comparisons of the model-derived estimated marginal means, and all reported *P*-values reflect this correction. For all analyses α = 0.05.

## Results

### Validation of Unsupervised Sleep Staging Algorithm

To validate our method of scoring, the computer-derived vigilance state classification results from 6 datasets were compared against scoring results from three trained humans. Because manual scoring by humans will always be sensitive to issues of subjectivity and scorer vigilance, an appropriate validation of automated methods should take into account how the computer-derived score compares to the inter-rater reliability of manual scoring. Consequently, the percent agreement between scores obtained from the computer algorithm and manual sleep staging were compared to the percent agreement between the manually-derived scores (inter-rater reliability; Fig 1C). There was no interaction between scorer (human vs. computer) and data file (repeated measure; 2-way ANOVA, *F*(5, 20) = 1.05, *p* = 0.42), and there was no effect of scorer (*F*(1, 4) = 1.01, *p* = 0.37). However, there was an effect of data file (*F*(5, 20) = 20.76, *p* < 0.001), because data file 4 was intentionally included as it had a noisy EMG signal. Compared to the other files that were scored, there was a marked reduction in the inter-rater reliability between humans and between human vs computer derived scores. Comparisons of scoring reliability for each vigilance state also found no difference between humans and the computer (S3 Fig). Consequently, we conclude that this algorithm performs comparably to manual sleep staging.

**Fig 2:**
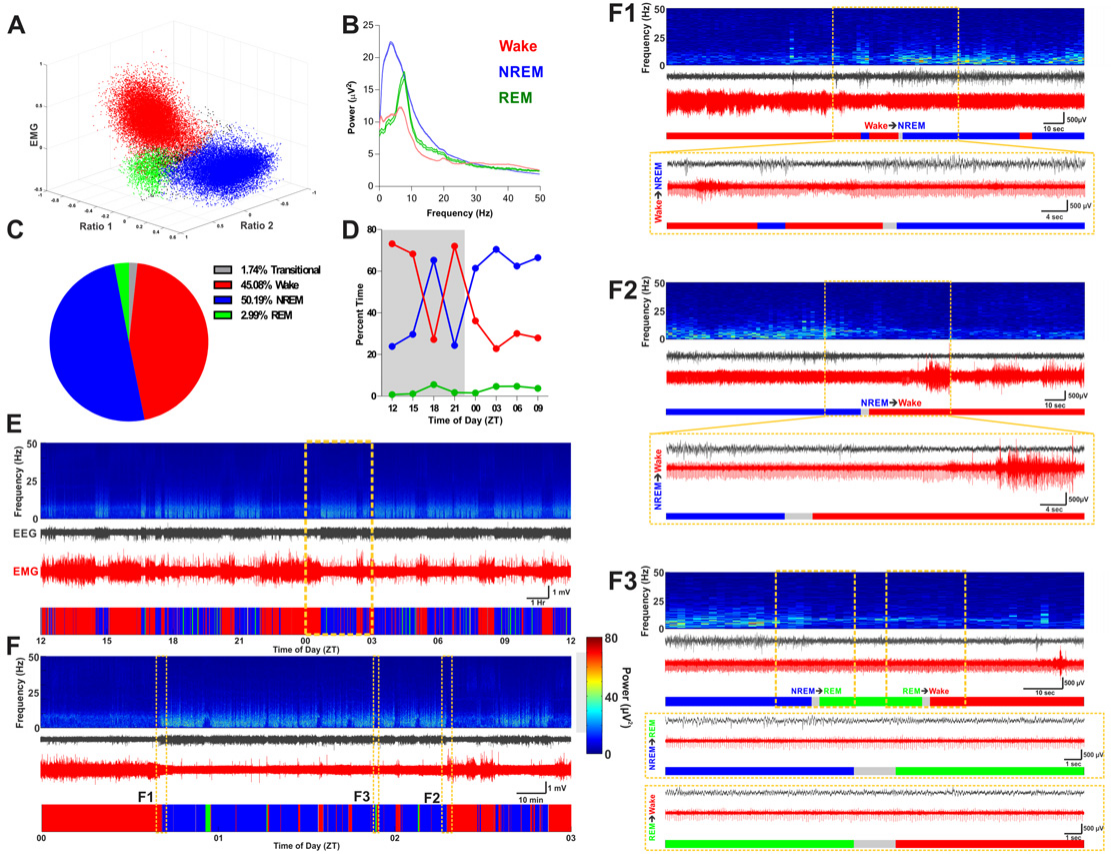
Example Results of State-Scoring. **A**, Example of scored state-space with color-coded state clusters. **B**, Example of power spectra derived from the scored epochs. Power spectra are color-coded according to the state they were derived from. Solid lines indicate borders of 95% confidence interval of power spectra for all epochs of associated state across the day. **C**, Distribution of time spent in each classification criteria over the day. **D**, Pattern of sleep-wake states over the day shown as percent time of 3 Hr bins. Grey background denotes the dark photoperiod. **E,** Aligned time-frequency power spectrum, raw EEG, raw EMG, and color-coded hypnogram for a single recording day. Time of day denoted as zeitgeber time underneath the hypnogram. **F**, Expanded view of hashed yellow box in panel E. Small yellow hashed boxes highlight times with state transitions and correspond to subpanels F1 –F3. **F1**, Wake to NREM transition. **F2,** NREM to wake transition. **F3,** Transition from NREM to REM and transition from REM to wake. For A-D and all hypnograms shown in E–F, wake is indicated in red, NREM is indicated in blue, and REM is indicated in green. For all periodograms shown in E and F, absolute power specified in the heat map is given by the colorbar between panel F and subpanel F3.

Fig 2 shows example scoring results with raw data traces and power spectra including state transitions. One important feature of this vigilance state-scoring program is the necessary inclusion of unclassified/transitional epochs that cannot be assigned to specific states with any rigor (note the black points *between* clusters in Fig 2A). This derives naturally from the fact that state clusters are not cleanly segregated in the state-space, which is consistent with the intuitive notion that state-transitions are not instantaneous (i.e. falling asleep or waking up takes some time as cortical ensembles synchronize or desynchronize, respectively). Thus, the algorithm conservatively estimates vigilance states by only assigning a score when an epoch registers within some statistical bounds of certainty.

### Direct Activation of CB1 Receptors Facilitates NREM Sleep

**Fig 3:**
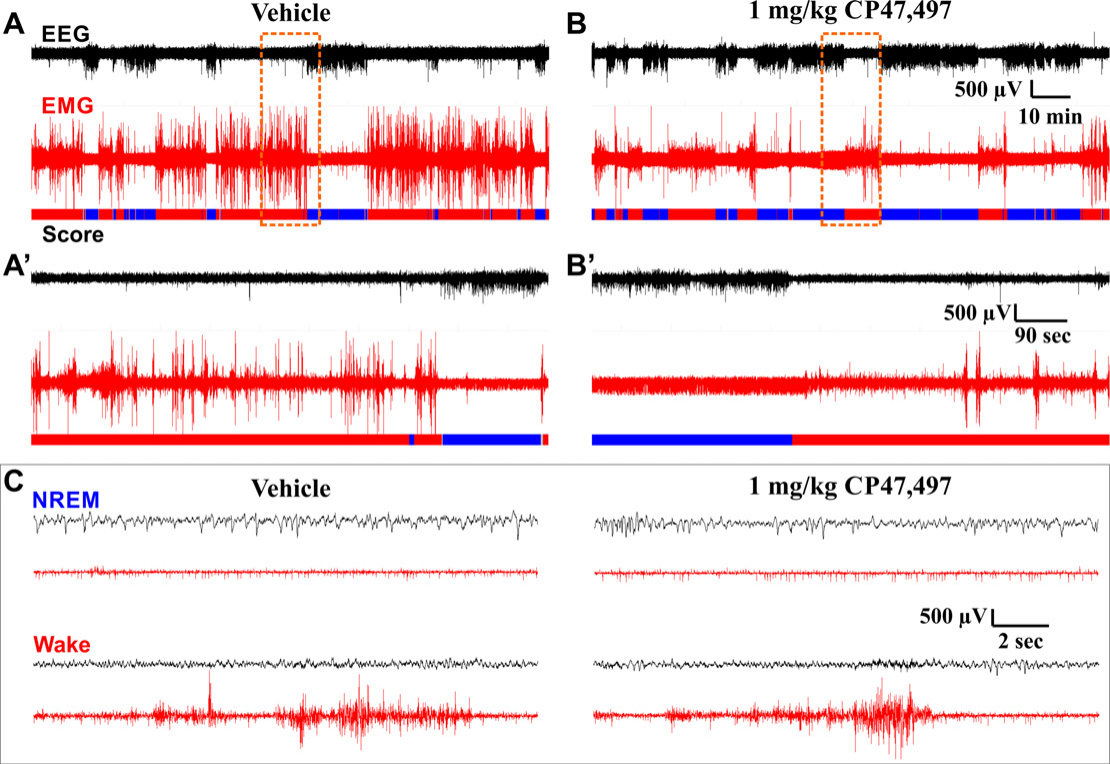
Example EEG/EMG Traces on Different Time Scales Following Vehicle or CP47,497 Administration. EEG and EMG traces are from the same subject at the same stage of the circadian cycle after administration of either vehicle (**A**, **A’**, and left column of **C**) or 1 mg/kg CP47,497 (**B**, **B’**, and right column of **C**). Panels **A** and **B** show a 2 Hr 15 min window from ZT 14:00–16:15, roughly 2 Hr after drug administration, coinciding with peak effects observed on sleep. Panels **A’** and **B’** show a 15 min long segment expanded from the region in **A** and **B** highlighted by the dashed orange box. Panel **C** shows representative 18 sec long data segments corresponding to NREM and Wake obtained following vehicle and CP47 administration. These data segments were taken from the segments shown in **A** and **B**. The color-coded hypnogram shown at the bottom of **A**, **B**, **A’**, and **B’** represents consecutive 2 sec epochs shown as wake (red), NREM (blue), unclassified (grey). No REM occurred during this period. Black traces depict EEG, red traces depict EMG. **A** and **B** are identically scaled. **A’** and **B’** are identically scaled. All traces in **C** are identically scaled.

**Fig 4:**
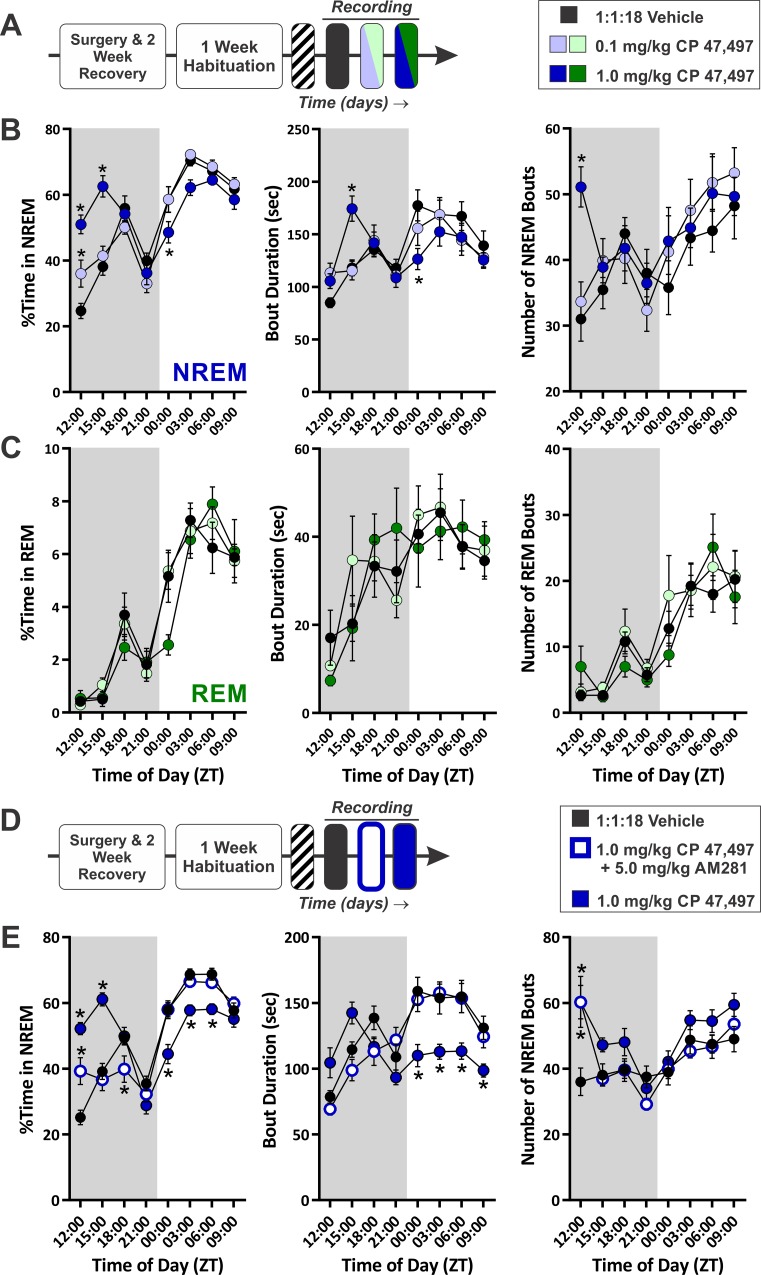
Direct Activation of CB1 with the Full Agonist, CP47,497, Has Biphasic Effects on NREM Sleep that are Mediated by the CB1 Receptor. **A,** Diagram of experimental protocol for recording sleep after administration of the CB1 agonist, CP47,497. All injections given at the onset of the dark photoperiod (ZT 12:00). **B,** Quantification of NREM sleep time and architecture (*N* = 9). **C,** Quantification of REM sleep time and architecture. **D,** Diagram of experimental protocol for recording sleep after co-administering CP47 with AM281. **E,** Quantification of NREM sleep in experiments with co-administration of CP47 and AM281. In all graphs, the grey shaded region denotes the dark photoperiod. Symbols represent mean±SEM for 3 Hr time bins. Asterisks denote significant difference from vehicle baseline.

To determine how activation of CB1 affects sleep, the full CB1 agonist, CP47, was administered just prior to the DP. Consistent with reports that CB1 activation reduces locomotor activity, phasic muscle movements in the EMG were reduced after injection of CP47, and the amount of high voltage, low frequency activity in the EEG was increased (Fig 3). In this experiment, a 0.1 (low) and a 1.0 (high) mg/kg dose of CP47 were administered on subsequent recording days following a baseline day where vehicle was injected (Fig 4A). We assessed the percent time spent in NREM sleep (Fig 4B) and found a significant overall interaction (treatment x time of day within photoperiod, *F*(18, 142.63) = 9.804, *p* < 0.001), secondary interaction (treatment x photoperiod, *F*(2, 96.81) = 26.63, *p* < 0.001), and a main effect of photoperiod (*F*(1, 116.62) = 284.59, *p* < 0.001). High dose CP47 had biphasic effects on sleep time, inducing significantly more NREM during the DP (*t*(85.57) = 5.71, *p* < 0.001) and reducing NREM during the LP (*t*(85.57) = -6.046, *p* = 0.006). NREM sleep time was increased over the first 6 Hr of the DP (low dose, ZT12-15: *t*(191.94) = 2.89, *p* = 0.009; high dose, ZT12-18: *t*(191.94) ≥ 6.21, *p* < 0.001), and high dose CP47 significantly reduced NREM during the first 3 Hr of the LP (ZT00-03: *t*(191.94) = -2.54, *p* = 0.024). Thus, the synthetic cannabinoid CP47 biphasically modulates NREM sleep time.

We next examined effects on NREM architecture. For NREM bout duration, there was an overall interaction (treatment x time of day within photoperiod, *F*(18, 143.87) = 3.854, *p* < 0.001), a secondary interaction (treatment x photoperiod, *F*(2, 78.01) = 4.85, *p* = 0.010), and a main effect of photoperiod (*F*(1, 113.84) = 26.537, *p* < 0.001). CP47 significantly increased NREM bout duration during the second quarter of the DP (ZT15-18: *t*(157.46) = 3.49, *p* = 0.001). In contrast, NREM bout duration was reduced across the LP (*t*(62.72) = 3.23, *p* = 0.032), specifically during the first quarter of the LP (ZT00-03: *t*(157.46) = 3.16, *p* = 0.004). The number of NREM bouts was also affected by an overall interaction (*F*(18, 143.48) = 1.96, *p* = 0.016) and a main effect of photoperiod (*F*(1, 113.30) = 17.81, *p* < 0.001). However, the only significant difference between treatment conditions occurred during the first quarter of the LP when high dose CP47 increased the number of NREM bouts (*t*(157.72) = 3.74, *p* < 0.001). Treatment with CP47 did not affect REM sleep (Fig 4C).

To confirm that CP47’s effects on sleep were mediated through the CB1 receptor, a separate cohort of subjects was administered the vehicle solution followed 24 Hr later by an injection containing a mixture of CP47 (1 mg/kg) and the selective CB1 antagonist, AM281 (5 mg/kg; CP47+AM281; Fig 4D). As an internal positive control, the experiment was continued for a third day when CP47 (1 mg/kg) was administered alone. There was an overall interaction for NREM sleep time (Fig 4E; treatment x time of day within photoperiod, *F*(18, 198.22) = 11.31, *p* < 0.001), a secondary interaction (treatment x photoperiod, *F*(2, 133.28) = 43.47, *p* < 0.001), and a main effect of photoperiod (F(1, 160.97) = 364.21, *p* < 0.001). While CP47 alone increased sleep time during the DP (ZT12-15 & ZT15-18: *t*(263.34) ≥ 6.43, *p* < 0.001) and reduced it during the LP (ZT00-03, 03–06, and 06–09: t(263.34) ≤ -3.12, p ≤ 0.006), co-administering AM281 significantly attenuated the CP47 effects during the DP (AM281+CP47 vs CP47, ZT12-15, 15–18, and 18–21: t(263.34) ≤ -2.94, *p* ≤ 0.011) and reversed them completely during the LP (ZT00-03 and 03–06: *t*(263.34) ≥ 2.57, *p* ≤ 0.033). Compared to vehicle, CP47+AM281 had mixed effects on NREM in the DP (ZT12-15 (increased): *t*(263.34) = 4.14, *p* < 0.001; ZT18-21(decreased): *t*(263.34) = -2.79, *p* = 0.017), but there were no differences during the LP. These results replicated our previous findings on CP47’s biphasic effect on sleep, and co-administration of AM281 blocked this effect suggesting that CP47’s effects on NREM sleep are mediated through CB1.

The CP47-induced changes in sleep architecture were similarly blunted by co-administration of AM281. For NREM bout duration, there was a significant overall interaction (treatment x time of day within photoperiod, *F*(18, 202.64) = 5.487, *p* < 0.001), secondary interaction (treatment x photoperiod, *F*(2, 103.25) = 13.06, *p* < 0.001), and main effects of both drug treatment (*F*(2, 61.707) = 4.376, *p* = 0.017) and photoperiod (*F*(1, 169.28) = 45.05, *p* < 0.001). CP47 produced a large decrease in bout duration across all time points of the LP (CP47 vs vehicle, ZT00-12: *t*(182.30) ≤ -2.69, *p* ≤ 0.025) that was blocked by co-administration of AM281 (CP47+AM281 vs CP47, ZT00-09:: *t*(184.77) ≥ 3.30, *p* ≤ 0.003). CP47+AM281 did not change NREM bout duration relative to vehicle. The number of NREM bouts was affected by a significant overall interaction (treatment x time of day within photoperiod, *F*(18, 192.19) = 5.20, *p* < 0.001) and main effects of both photoperiod (*F*(1, 155.99) = 11.18, *p* = 0.001) and drug treatment (*F*(2, 54.76) = 4.79, *p* = 0.012). Across the entire recording day, CP47 produced an increase in the number of NREM bouts (*t*(48.65) = 2.98, *p* = 0.013), but the only specific time point with significant differences between treatments was the first quarter of the DP where both CP47 (*t*(176.71) = 4.58, *p* < 0.001) and CP47+AM281 (*t*(178.81) = -4.57, *p* < 0.001) produced a significant increase in NREM bouts. As discussed later, AM281 alone increases the number NREM bouts during the first quarter of the DP, so the increased number of NREM bouts is confounded by AM281’s effect. Thus, CP47’s effects on sleep architecture are largely mediated through the CB1 receptor.

### Inhibition of Monoacylglycerol Lipase Stabilizes NREM and Suppresses REM

#### Sleep Measurements

**Fig 5:**
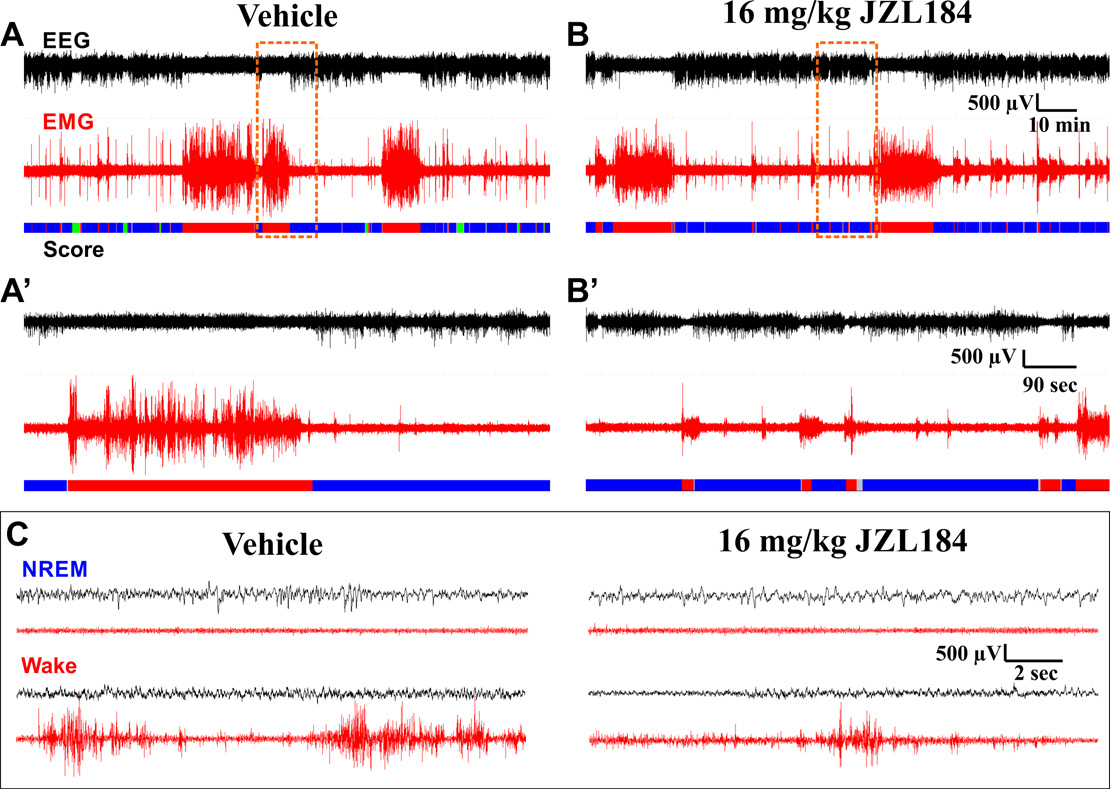
Example EEG/EMG Traces on Different Time Scales Following Vehicle or JZL184 Administration. EEG and EMG traces are from the same subject at the same stage of the circadian cycle after administration of either vehicle (**A**, **A’**, and left column of **C**) or 16 mg/kg JZL184 (**B**, **B’**, and right column of **C**). Data are from experiment with JZL administration before the LP. Panels **A** and **B** show a 2 Hr 15 min window from ZT 02:00–04:15, roughly 2 Hr after drug administration, coinciding with peak effects observed on sleep. Panels **A’** and **B’** show a 15 min long segment expanded from the region in **A** and **B** highlighted by the dashed orange box. Panel **C** shows representative 18 sec long data segments corresponding to NREM and wake obtained following vehicle and JZL administration. These data segments were taken from the segments shown in **A** and **B**. The color-coded hypnogram shown at the bottom of **A**, **B**, **A’**, and **B’** represents consecutive 2 sec epochs shown as wake (red), NREM (blue), REM (green), and unclassified (grey). Note the loss of REM sleep following JZL administration. Black traces depict EEG, red traces depict EMG. **A** and **B** are identically scaled. **A’** and **B’** are identically scaled. All traces in **C** are identically scaled.

**Fig 6:**
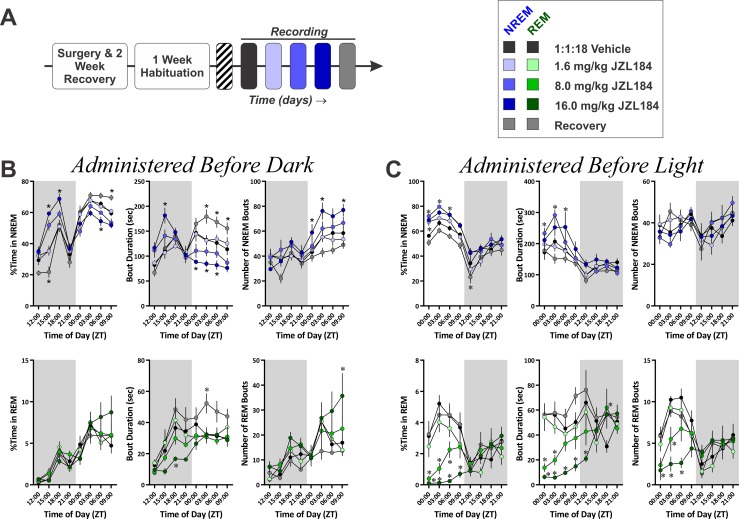
Differential Effects of Increasing 2-AG Tone on NREM and REM Sleep Based on Circadian Timing of Drug Administration. **A,** Diagram of experimental protocol for recording sleep after administration of the MAGL inhibitor, JZL184. **B,** Quantification of NREM (top row) and REM (bottom row) sleep time and architecture for experiment where JZL was administered prior to the DP (*N* = 10). **C**, Quantification of NREM (top row) and REM (bottom row) sleep time and architecture for experiment where JZL was administered prior to the LP (*N* = 8). In all graphs, the grey shaded region denotes the dark photoperiod. Symbols represent mean±SEM for 3 Hr time bins. Asterisks denote significant difference from vehicle baseline.

Considering that activation of CB1 receptors with exogenous ligands can facilitate sleep, we next sought to test the hypothesis that eCBs could similarly promote NREM sleep. Increasing endogenous 2-AG tone with JZL, a selective MAGL inhibitor, reduced phasic EMG activity and increased the amount of low-frequency high-voltage EEG activity characteristic of NREM sleep (Fig 5). To quantify vigilance states after pharmacologically increasing 2-AG levels, subjects were sequentially given a 1.6 (low), 8.0 (moderate), and 16.0 (high) mg/kg doses of JZL, after which an additional 24 Hr recording with no injection (recovery) was obtained (Fig 6A). Within-subject comparisons were made using sleep measures obtained during a 24 Hr baseline recording that followed a vehicle injection. Several reports have suggested that endocannabinoid levels fluctuate across the circadian cycle [33, 34, 42], but it is unclear how or if this may be related to sleep. Therefore, two experiments were performed with JZL in separate groups of mice. In one, JZL was administered before the DP, when mice are most active (Fig 6B), and in the other, JZL was administered prior to the LP (Fig 6C).

When given just before the onset of the DP (between ZT 11:30 and 12:00), JZL administration had obvious effects on NREM sleep that mirrored those seen with CP47 (Fig 6B; top row). This was evidenced by an overall interaction (treatment x time of day within photoperiod, *F*(30, 269.56) = 12.29, *p* < 0.001), secondary interaction (treatment x photoperiod, *F*(4, 198.66) = 48.31, *p* < 0.001), and a main effect of photoperiod (*F*(1, 234.92) = 596.81, *p* < 0.001). JZL had biphasic effects on NREM with increased sleep during the DP (moderate: *t*(172.80) = 4.49, *p* < 0.001; high: *t*(175.26) = 6.71, *p* < 0.001) and a suppression of NREM during the LP (moderate: *t*(172.80) = -3.72, *p* = 0.001; high: *t*(172.80) = -4.62, *p* < 0.001). Specifically, JZL increased NREM sleep during the middle of the DP (moderate, ZT15-18: *t*(357.53) = 4.25, *p* < 0.001; high, ZT15-21: *t*(357.53) ≥ 4.38, *p* < 0.001) and reduced it during the LP (moderate, ZT00-03: *t*(357.53) = -3.17, *p* = 0.007; high, ZT06-09: *t*(357.53) = -2.93, *p* = 0.014). In contrast, on the recovery day NREM was reduced during the DP (recovery vs vehicle, *t*(172.80) = -3.66, *p* = 0.001) and increased during the LP (*t*(172.8) = 2.55, *p* = 0.047), specifically during the last quarter of the LP (ZT09-12: *t*(357.53) = 2.64, *p* = 0.035). Thus, inhibition of MAGL has biphasic effects on NREM sleep, initially increasing the time in NREM, followed by a decrease in NREM that extends into the recovery day.

JZL also produced alterations in NREM architecture similar to CP47. For NREM bout duration, there was an overall interaction (treatment x time of day within photoperiod, *F*(30, 272.08) = 3.55, *p* < 0.001), a secondary interaction (treatment x photoperiod, *F*(4, 153.85) = 20.92, *p* < 0.001), and main effects of both treatment (*F*(4, 93.89) = 3.60, *p* = 0.009) and photoperiod (*F*(1, 230.16) = 4.065, *p* = 0.045). High dose JZL increased NREM bout duration across the DP (*t*(115.00) = 2.83, *p* = 0.022), and both moderate and high doses reduced it across the LP (*t*(115.00) ≤ -2.58, *p* ≤ 0.044). Specifically, high dose JZL increased bout duration during the second quarter of the DP (ZT15-18: *t*(286.90) = 4.29, *p* < 0.001) and reduced it across most of the LP (ZT00-09: *t*(286.90) ≤ -2.75, *p* ≤ 0.026). On the recovery day, NREM bout duration was increased across most of the LP (ZT03-12: *t*(287.54) ≥ 2.56, *p* ≤ 0.044). For the number of NREM bouts, there was also an overall interaction (treatment x time of day within photoperiod, *F*(30, 286.26) = 3.19, *p* < 0.001), a secondary interaction (treatment x photoperiod, *F*(4, 153.85) = 3.23, *p* = 0.014), and main effects of both drug treatment (*F*(4, 75.96) = 6.15, *p* < 0.001) and photoperiod (*F*(1, 272.44) = 99.44, *p* < 0.001). High dose JZL increased NREM bouts across the LP (t(95.33) = 3.57, p = 0.002) with pair-wise differences at two time points (ZT03-06 & ZT09-12: *t*(218.71) ≥ 2.77, *p* ≤ 0.024). Thus, the effects of JZL treatment on NREM sleep were closely mirrored by effects on NREM bout duration, suggesting that MAGL inhibition-induced changes in sleep are due to modulation of NREM stability.

In contrast, administration of JZL before the DP produced only a slight reduction in REM sleep parameters (Fig 6B; bottom row). There was no effect of JZL on REM sleep time. For the duration of REM bouts, there was a nested interaction (time of day within photoperiod, *F*(6, 238.62) = 10.81, *p* < 0.001), and main effects of treatment (*F*(4, 82.54) = 7.01, *p* < 0.001) and photoperiod (*F*(1,238.51) = 34.78, *p* < 0.001). 16.0 mg/kg JZL reduced REM bout duration during across the DP (*t*(110.53) = -2.56, *p* = 0.047), specifically during the third quarter of the DP (ZT18-21: *t*(235.00) = -2.80, *p* = 0.022), and REM bout duration was increased across the LP on the recovery day (*t*(99.54) = 2.77, *p* = 0.027), specifically during the second quarter of the LP (ZT03-06: *t*(237.32) = 2.71, *p* = 0.022). For the number of REM bouts, there was a nested interaction (time of day within photoperiod, (*F*(6, 268.06) = 14.44, *p* < 0.001) and main effects of treatment (*F*(4, 81.95) = 3.17, *p* = 0.018) and photoperiod (*F*(1,254.72) = 55.42, *p* < 0.001). The high dose of JZL increased the number of REM bouts during the last 3 Hr of the LP (ZT09-12: *t*(240.24) = 3.72, *p* = 0.001).

When JZL was administered before the LP, NREM sleep was augmented (Fig 6C; top row), but the magnitude of this effect (deviation from vehicle baseline) was not as great as when the drug was given prior to the DP. The effect was also not biphasic within a circadian cycle. For the percent of time spent in NREM sleep, there was a secondary interaction (treatment x photoperiod, *F*(4, 165.01) = 5.00, *p* = 0.001) and a nested interaction (time of day within photoperiod, *F*(6, 209.40) = 22.04, *p* < 0.001) along with main effects of both treatment (*F*(4, 126.24) = 33.05, *p* < 0.001) and photoperiod (*F*(1, 192.72) = 522.51, *p* < 0.001). Moderate and high dose JZL increased NREM sleep time across the LP (*t*(145.25) ≥ 4.92, *p* < 0.001), while NREM sleep time was reduced on the recovery day during both the LP (*t*(145.26) = -3.36, *p* = 0.004) and DP (*t*(145.26) = -3.61, *p* = 0.002). Specifically, all three doses of JZL increase NREM sleep time during the first 3 Hr of the LP (ZT 00–03: *t*(274.85) ≥ 2.59, *p* ≤ 0.040) with the moderate dose increasing NREM sleep up to 6 Hr after administration (*t*(274.85) = 3.06, *p* = 0.010) and the high dose increasing NREM up to 9 Hr into the LP (*t*(274.85) = 2.52, *p* < 0.050). For NREM bout duration, there was an overall interaction (treatment x time of day within photoperiod; *F*(24, 218.31) = 1.67, *p* = 0.030), a secondary interaction (treatment x photoperiod; *F*(4, 120.79) = 2.80, *p* = 0.029), a nested interaction (time of day within photoperiod; *F*(6, 20.50) = 8.02, *p* < 0.001), and main effects of both treatment (*F*(4, 74.15) = 6.34, *p* < 0.001) and photoperiod (*F*(1, 179.88) = 132.73, *p* < 0.001). High and moderate dose JZL increased NREM bout duration across the LP (*t*(91.06) ≥ 2.94, *p* ≤ 0.016). The moderate dose of JZL increased NREM bout duration across the first 6 Hr of the LP (ZT00-06: *t*(225.33) ≥ 2.59, *p* ≤ 0.041), while high dose JZL only increased NREM bout duration later in the LP (ZT06-09: *t*(225.33) = 3.12, *p* = 0.008). The number of NREM bouts was not affected when JZL was administered before the LP.

In contrast to the modest effects of JZL on REM sleep when the drug was given before the DP, administration of JZL before the LP produced a marked reduction in REM sleep (Fig 6C; bottom row). For the percent of time spent in REM sleep, there was an overall interaction (treatment x time of day within photoperiod, *F*(24, 224.83) = 1.61, *p* = 0.040), secondary interaction (treatment x photoperiod, *F*(4, 116.88) = 13.58, *p* < 0.001), nested interaction (time of day within photoperiod, *F*(6, 212.50) = 9.60, *p* < 0.001), and main effects of both treatment (*F*(4, 61.32) = 7.17, *p* < 0.001) and photoperiod (*F*(1, 200.12) = 10.77, *p* = 0.001). Both moderate and high dose JZL reduced REM sleep time across the LP (*t*(77.11) ≤ -4.81, *p* < 0.001). Specifically, REM sleep was diminished across most time points in the LP following administration of both doses (moderate dose, ZT00-09: *t*(184.07) ≤ -2.81, *p* ≤ 0.022; high dose, ZT00-12: *t*(184.05) ≤ -3.87, *p* ≤ 0.001). For REM bout duration, there was an overall interaction (treatment x time of day within photoperiod, *F*(24, 192.48) = 2.02, *p* = 0.005), secondary interaction (treatment x photoperiod, *F*(4, 113.74) = 6.52, *p* < 0.001), and main effects of both treatment (*F*(4, 70.319) = 11.95, *p* < 0.001) and photoperiod (*F*(1, 165.44) = 15.90, *p* < 0.001). REM bout duration was suppressed by both moderate and high dose JZL across the LP (*t*(85.623) ≤ -27.46, *p* ≤ 0.001). Specifically, the moderate dose of JZL reduced REM bout duration during the first 6 Hr of the LP (ZT00-06: *t*(217.98) ≤ −3.27, *p* ≤ 0.005) and increased REM bout duration during the middle of the DP (ZT18-21: *t*(217.49) = 2.75, *p* = 0.026). High dose JZL reduced REM bout duration across the entire LP and into the first 3 Hr of the DP (ZT00-15: *t*(214.55) ≤ −3.21, *p* ≤ 0.006). For the number of REM bouts, there was a secondary interaction (treatment x photoperiod, *F*(4, 119.91) = 10.01, *p* < 0.001), nested interaction (time of day within photoperiod, *F*(6, 206.20) = 11.17, *p* < 0.001), and main effects of both treatment (*F*(4, 74.09) = 3.14, *p* = 0.019) and photoperiod (*F*(1, 177.54) = 27.29, *p* < 0.001). Again, moderate and high dose JZL reduced the number of REM bouts across the LP (*t*(90.894) ≤ -3.41, *p* ≤ 0.002). The number of REM bouts was reduced at multiple time points during the LP following JZL administration (moderate dose, ZT03-06: *t*(227.24) = 3.19, *p* < 0.001); high dose, ZT00-09: *t*(227.24) ≤ -2.85, *p* ≤ 0.019). Thus, REM sleep is markedly suppressed by acute augmentation of 2-AG tone, but only when this drug is administered immediately before the time of day when mice engage in most of their REM sleep.

#### EEG Power Spectral Measurements

**Fig 7:**
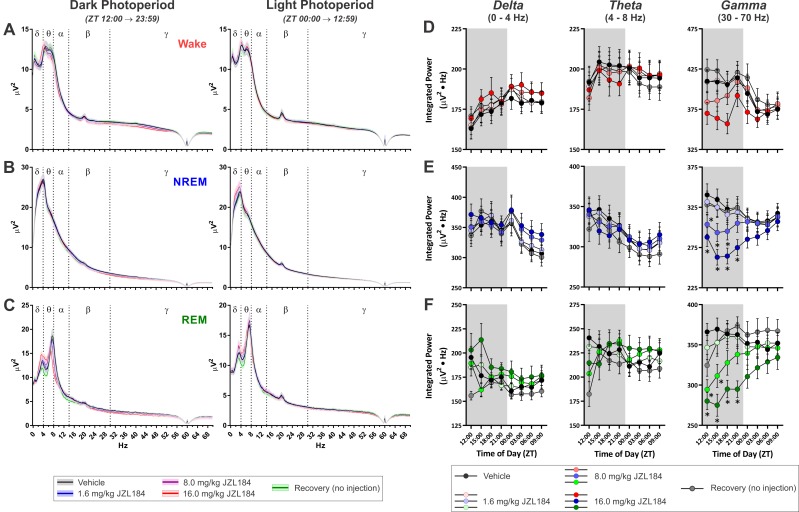
MAGL Inhibition with JZL184 Attenuates Gamma Frequency Oscillations During Sleep. **A-C**, Average power spectra for epochs of different vigilance states across the entire DP (left hand) and LP (right hand). Solid lines denote means and shaded region around lines denotes SEM. **A**, Wake. **B**, NREM. **C**, REM. **D-F**, Change over the day in summated power in different frequency bandwidths from the power spectra: delta (left hand column), theta (middle column), and gamma (right hand column). In these graphs, results from epochs of wake are denoted in red (**D**), NREM are in blue (**E**), and REM are in green (**F**). Symbols/Bars represent mean±SEM for 3 hr time bins (*N* = 10). Grey background in graphs shows dark photoperiod. Asterisks denote significant difference from vehicle baseline. All injections administered at onset of DP (ZT 12:00).

Given the effects of increased 2-AG signaling on NREM and REM sleep described above, we examined the spectral content of the EEG signal from the experiment where JZL was administered before the DP (Fig 7). Similar results were obtained when JZL was administered before the LP (S4 Fig) and when CP47 was administered before the DP (S5 Fig). Despite the robust effects on sleep, JZL produced relatively modest effects on 12 Hr averages of EEG power spectrum from epochs of any state (Fig 7A–7C). To quantify JZL’s effects on EEG power spectra with higher temporal precision, we summed across well-described power spectral bandwidths (delta: 0-4Hz, theta: 4–8 Hz, and gamma: 30–70 Hz) in 3 Hr time bins (Fig 7D–7F). These bandwidths are routinely associated with sleep homeostasis (delta [43, 44], theta [45]), pneumonic processes (theta [46]), and attention (gamma [47]). Treatment with JZL had no effect on delta, theta, or gamma power during wake epochs (Fig 7D). For NREM epochs (Fig 7E), there was no effect of JZL on delta power, but for theta power there was a significant overall interaction (treatment x time of day within photoperiod, *F(*24, 335.61) = 1.84, *p* = 0.010), a nested interaction (time of day within photoperiod, *F*(6, 304.79) = 9.24, *p* < 0.001), and a main effect of photoperiod (F(1, 159.84) = 85.90, p < 0.001). However, there were no specific time points where JZL significantly altered NREM theta power relative to vehicle. For NREM gamma power, there was an overall interaction (treatment x time of day within photoperiod, *F*(24, 344.26) = 3.21, *p* < 0.001), a secondary interaction (treatment x photoperiod, *F*(4, 354.88) = 14.62, *p* < 0.001), a nested interaction (time of day within photoperiod, *F*(6, 304.49) = 25.78, *p* < 0.001), and main effects of both drug treatment (*F*(4, 220.36) = 10.85, *p* < 0.001) and photoperiod (*F*(1, 168.31) = 5.16, *p* = 0.024). JZL184 produced a dose-dependent reduction of NREM gamma power, with 8.0 mg/kg JZL184 decreasing gamma during the first 9 Hr of the DP (ZT 12–21: *t*(240.60) ≤ -2.67, *p* ≤ 0.032) and 16.0 mg/kg JZL reducing NREM gamma across the entire DP (ZT 12–00: *t*(159.50) ≤ -4.14, *p* ≤ 0.001). NREM gamma was no different from vehicle following the 1.6 mg/kg dose or on the recovery day. For REM epochs (Fig 7F), there was an there was an overall interaction (treatment x time of day within photoperiod, *F*(24, 284.87) = 1.71, *p* ≤ 0.022) and a main effect of photoperiod (*F*(1, 306.75) = 16.23, *p* < 0.001) for delta power. However, there were no pair-wise differences between treatment/recovery conditions and vehicle. Similarly, for REM theta power, there was an overall interaction (treatment x time of day within photoperiod, *F*(24, 293.65) = 2.36, *p* < 0.001) and a nested interaction (time of day within photoperiod, *F*(6, 292.49) = 8.09, *p* < 0.001), but there were no pair-wise differences between treatment/recovery conditions and vehicle. For REM gamma, there was a secondary interaction (treatment x photoperiod, *F*(4, 252.78) = 5.03, *p* = 0.001) and a nested interaction (time of day within photoperiod, *F*(6, 292.39) = 10.94, *p* < 0.001) with main effects of drug treatment (*F*(4, 83.77) = 7.39, *p* < 0.001) and photoperiod (*F*(1, 235.60) = 15.65, *p* < 0.001). JZL reduced REM gamma in a dose-dependent manner with 8.0 mg/kg JZL184 decreasing REM gamma across the first half of the DP (ZT12-18: *t*(121.00) ≤ -3.04, *p* ≤ 0.011) and 16.0 mg/kg JZL184 decreasing REM gamma across the entire DP (ZT12-00: *t*(85.92) ≤ -3.64, *p* ≤ 0.002). There was no difference in REM gamma power following low dose JZL184 or on the recovery day. Thus, increasing endogenous 2-AG tone with JZL has little effect on delta or theta power, but it attenuates gamma oscillations, particularly during sleep. Additionally, this effect is consistent irrespective of the time of day JZL is administered (see S4 Fig).

### Inhibition of Fatty Acid Amide Hydrolase Stabilizes NREM and Suppresses REM

#### Sleep Measurements

**Fig 8:**
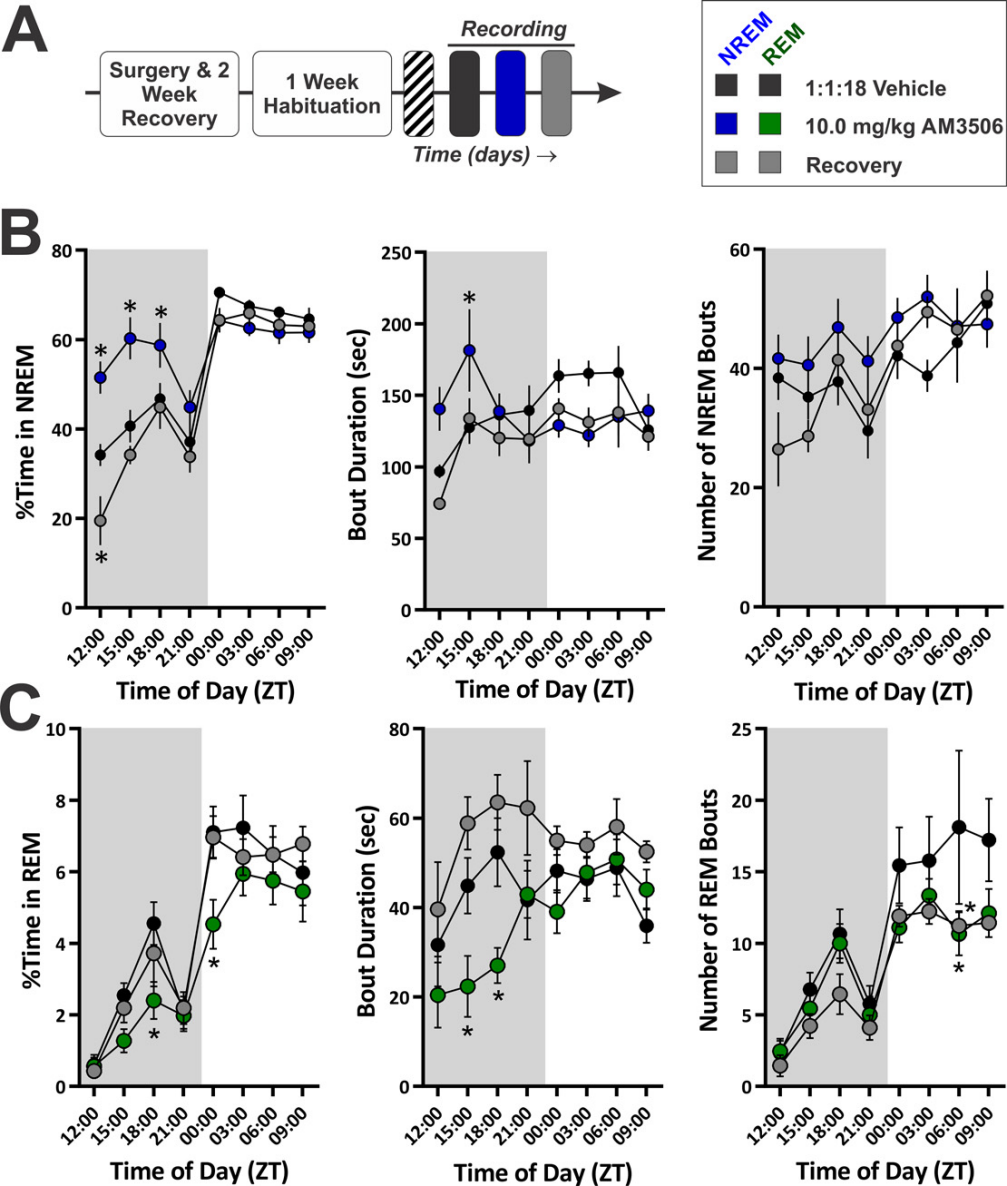
FAAH Inhibition with AM3506 Increases NREM Sleep Time and Stability while Decreasing REM Sleep. **A,** Diagram of experimental protocol for recording sleep after administration of the long-lasting FAAH inhibitor AM3506. **B,** Quantification of NREM sleep time and architecture for the AM3506 experiment (*N* = 9). **C,** Quantification of REM sleep time and architecture. In all graphs, the grey shaded region denotes the dark photoperiod. Symbols represent mean±SEM for 3 Hr time bins. Asterisks denote significant difference from vehicle baseline. All injections administered at onset of dark photoperiod (ZT 12:00).

Next, we tested the hypothesis that endogenous *N*-acylethanolamines, including AEA, could modulate sleep. The FAAH inhibitor URB was injected systemically at three different doses (0.1, 1.0, and 10.0 mg/kg) over successive days (S6 Fig, panel A), but URB did not have a substantial effect on either NREM (S6 Fig, panel B) or REM (S6 Fig, panel C) sleep, in contrast to the counterintuitive effects of i.c.v. injection reported previously [18]. These data would appear to suggest that *N*-acylethanolamines are not important for the regulation of vigilance states. However, application of exogenous AEA is known to facilitate NREM sleep [14, 15], and the elevation of *N*-acylethanolamines in rodent brain tissue by URB lasts only a few hours [48]. Thus, we performed a separate experiment with a single dose of the selective, long-lasting FAAH inhibitor AM3506 (10.0 mg/kg; Fig 8A) that reduces FAAH activity for up to 10 days after administration [49]. In this experiment, subjects were administered a vehicle injection followed 24 Hr later by an injection of AM3506, and polysomnographic measures of sleep were obtained over the following 48 Hr. As shown in Fig 8B, AM3506 significantly altered NREM sleep. For NREM sleep time, there was a significant overall interaction (treatment x time of day within photoperiod, *F*(18, 142.90) = 3.68, *p* < 0.001), a secondary interaction (treatment x photoperiod, *F*(2, 80.60) = 20.57), and main effects of both treatment (*F*(2, 56.53) = 11.63, *p* < 0.001) and photoperiod (*F*(1, 111.44) = 231.09, *p* < 0.001). During the DP, AM3506 significantly augmented NREM sleep time (*t*(66.28) = 5.16, *p* < 0.001), and similar to the effect of JZL, there was a significant reduction in NREM sleep during the DP on the recovery day (*t*(66.63) = -2.41, *p* = 0.038). In contrast to the effects of JZL and CP47, NREM sleep time during the LP was unaffected. Pair-wise comparisons at individual time bins found AM3506 significantly increased NREM sleep across the first 9 Hr of the DP (ZT12-21: *t*(168.29) ≥ 2.64, *p* ≤ 0.018), and on the recovery day, NREM sleep time was significantly reduced during the first three hours of the DP (ZT12-15: *t*(168.35) = -3.25, *p* = 0.003). Thus, increasing *N*-acylethanolamine signaling with long-lasting inhibition of FAAH increases NREM sleep time, but this does not produce the biphasic effect seen with JZL and CP47.

To ascertain the effect of FAAH inhibition on NREM architecture, we measured the number and duration of NREM bouts at 3 Hr time points across the circadian cycle (Fig 8B). There was an overall interaction for NREM bout duration (treatment x time of day within photoperiod, *F*(18, 148.23) = 2.79, *p* < 0.001) and a secondary interaction between treatment and photoperiod (*F*(2, 75.68) = 3.54, *p* = 0.034). There was a significant increase in NREM bout duration during the second quarter of the dark photoperiod (ZT15-18: *t*(135.38) = 2.77, *p* = .013). The number of NREM bouts was not affected by treatment with AM3506. These findings demonstrate that FAAH inhibition promotes sleep by increasing NREM stability shortly after drug administration.

Similar to JZL, AM3506 reduced REM sleep (Fig 8C). For the percent time spent in REM, there was a nested interaction (time of day within photoperiod, *F*(6, 139.71) = 7.51, *p* < 0.001) and main effects of treatment (*F*(2, 51.93) = 4.399, *p* = 0.017) and photoperiod (*F*(1, 112.07) = 227.69, *p* < 0.001). Overall, AM3506 reduced REM sleep (*t*(47.65) = -2.75, *p* = 0.017), specifically during the third quarter of the DP (ZT18-21: *t*(158.67) = -2.54, *p* = 0.024) and first quarter of the LP (ZT00-03: *t*(158.67) = -3.05, *p* = 0.005). For the duration of REM bouts, there was a nested interaction (time of day within photoperiod, *F*(6, 132.64) = 3.99, *p* = 0.001) and main effects of treatment (*F*(2, 59.13) = 10.66, *p* < 0.001) and photoperiod (*F*(1,105.96) = 7.72, *p* = 0.006). Overall, REM bouts were longer on the recovery day (*t*(60.507) = 2.74, *p* = 0.016), but AM3506 reduced REM bout duration across the DP (*t*(73.75) = -2.70, *p* = 0.017), specifically during the middle of the DP (ZT15-21: *t*(170.06) ≤ -2.61, *p* ≤ 0.020). Finally, for the number of REM bouts, there was a nested interaction (time of day within photoperiod, *F*(6, 142.95) = 5.23, *p* < 0.001) and main effects of treatment (*F*(2, 46.33) = 4.39, *p* = 0.018) and photoperiod (*F*(1, 120.03) = 84.76, *p* < 0.001). The number of REM bouts during the LP was reduced by AM3506 and on the recovery day (*t*(55.64) ≤ -2.80, *p* ≤ 0.014), specifically during the third quarter of the LP (ZT06-09: *t*(139.96) ≤ -2.68, *p* ≤ 0.017).

#### EEG Power Spectral Measurements

The results of power spectral analysis of the EEG from the AM3506 experiment were very similar to those obtained following CP47 and JZL administration (S7 Fig). Specifically, increasing AEA tone with AM3506 had modest effects on delta and theta bandwidths, but it reduced gamma power during NREM and REM epochs. Thus, the attenuation of gamma oscillations, particularly during sleep, seems to be a consistent effect of increased eCB signaling.

### Blockade of CB1 Fragments NREM Sleep and Substantially Alters Power Spectral Features of the EEG

#### Sleep Measurements

**Fig 9:**
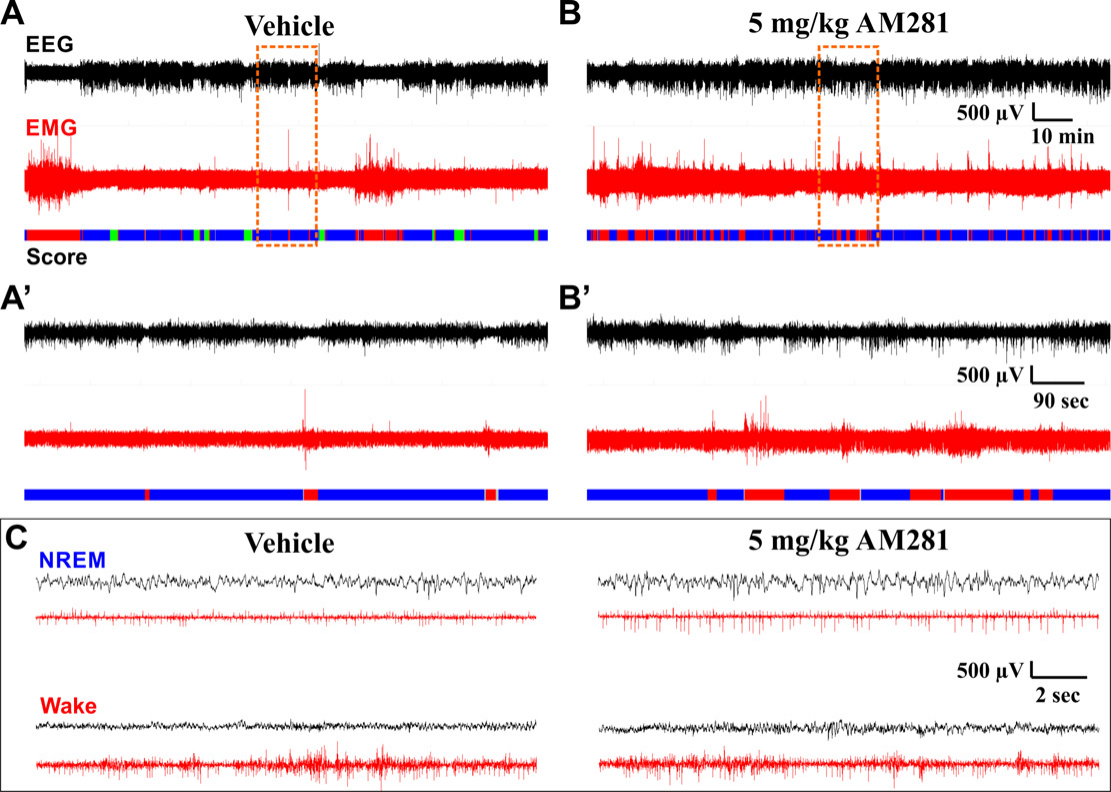
Example EEG/EMG Traces on Different Time Scales Following Vehicle or AM281 Administration. EEG and EMG traces are from the same subject at the same stage of the circadian cycle after administration of either vehicle (**A**, **A’**, and left column of **C**) or 5 mg/kg AM281 (**B**, **B’**, and right column of **C**). Data are from experiment with AM281 administration before the LP. Panels **A** and **B** show a 2 Hr 15 min window from ZT 00:15–02:30, roughly 15–30 min after drug administration, coinciding with peak effects observed on sleep. Panels **A’** and **B’** show a 15 min long segment expanded from the region in **A** and **B** highlighted by the dashed orange box. Panel **C** shows representative 18 sec long data segments corresponding to NREM and wake obtained following vehicle and AM281 administration. These data segments were taken from the segments shown in **A** and **B**. The color-coded hypnogram shown at the bottom of **A**, **B**, **A’**, and **B’** represents consecutive 2 sec epochs shown as wake (red), NREM (blue), REM (green), and unclassified (grey). Note the loss of REM sleep and fragmentation of NREM following AM281 administration. Black traces depict EEG, red traces depict EMG. **A** and **B** are identically scaled. **A’** and **B’** are identically scaled. All traces in **C** are identically scaled.

**Fig 10:**
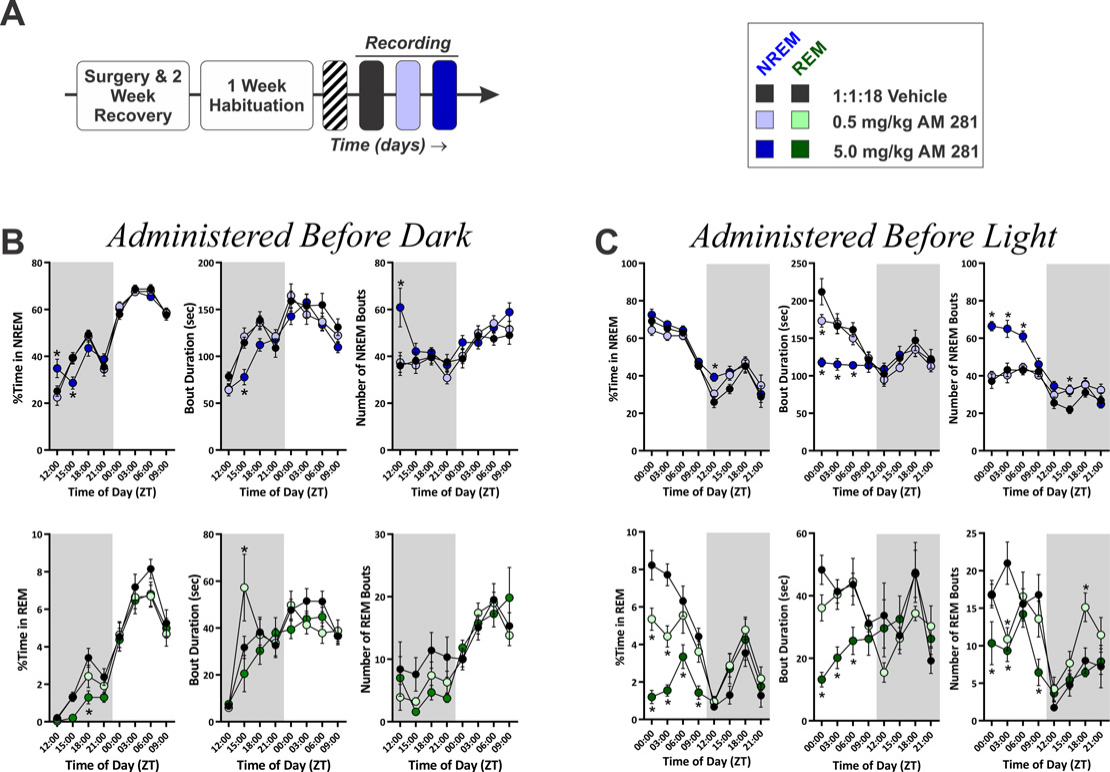
Blockade of CB1 Has Minimal Effects on NREM Sleep Time but Fragments NREM, Resulting in Reduced REM Sleep. **A,** Schematic overview of experimental paradigm. **B,** Effect of administering different doses of AM281 at the onset of the DP (ZT 12:00, *N* = 12). **C,** Effect of administering different doses of AM281 at the onset of the LP (ZT 00:00, *N* = 9). **B & C,** Measures of NREM (top row) and REM (bottom row) sleep time and architecture are shown. In all graphs, grey shaded regions denote the the DP. Asterisks (*) denote significant pair-wise comparisons between drug conditions and measures obtained during vehicle baseline. Symbols represent means±SEM across all subjects for each 3 Hr time bin.

To determine if eCB/CB1 signaling is necessary for the normal circadian fluctuation in NREM and REM sleep, we performed experiments with the full, selective CB1 antagonist/inverse agonist AM281. Following AM281 administration, there was a substantial fragmentation of NREM sleep and a loss of REM, particularly when this drug was administered prior to the LP (Fig 9). Again, given that eCBs exhibit a circadian fluctuation that differs by brain region, it was not easy to predict *a priori* an optimal time to administer the drug, so two separate experiments were performed where AM281 was given at opposite points in the circadian phase, immediately before either the LP or DP. In both experiments, two doses of AM281, 0.5 mg/kg (low) and 5.0 mg/kg (high), were administered sequentially on consecutive days following a baseline day when vehicle was given (Fig 10A).

When AM281 was administered prior to the DP (Fig 10B, top row), NREM sleep time was affected by an overall interaction (treatment x time of day within photoperiod, *F*(18, 198.82) = 10.27, *p* < 0.001) with a main effect of photoperiod (*F*(1, 169.19) = 836.77, *p* < 0.001). Only the high dose of AM281 produced effects on NREM sleep time, and the magnitude of these effects was small. Specifically, there was an increase in NREM sleep time during the first quarter of the DP (*t*(261.07) = 2.93, *p* = 0.007) and a decrease during the second quarter (*t*(261.09) = -3.20, *p* = 0.003). The effect on NREM bout duration was more pronounced with a significant overall interaction (treatment x time of day within photoperiod, *F*(18, 201.36) = 7.20, *p* < 0.001) and main effects of both treatment (*F*(2, 69.38) = 3.86, *p* = 0.026) and photoperiod (*F*(1, 163.20) = 86.17, *p* < 0.001). Following administration of high dose AM281, there was an overall reduction in NREM bout duration (*t*(72.79) = -2.64, *p* = 0.020) with a significant reduction during the second quarter of the DP (ZT15-18: *t*(205.68) = -3.02, *p* = 0.006). The number of NREM bouts was also affected by an overall interaction (*F*(18, 204.63) = 3.46, *p* < 0.001) with main effects of both treatment (*F*(2, 61.58) = 3.30, *p* = 0.043) and photoperiod (*F*(1, 173.11) = 25.90, *p* < 0.001). However, this result was largely driven by an increased number of NREM bouts during the first quarter of the DP following high dose AM281 (ZT612-15: *t*(181.59) = 4.81, *p* < 0.001). Thus, administration of AM281 prior to the DP yields subtle effects on sleep time but decreases the stability of NREM bouts.

REM sleep was disrupted by AM281, but the effect size was small as baseline REM is normally very low during the DP, when mice are most active (Fig 10B, bottom row). For the percent of time spent in REM, there was a nested interaction (time of day within photoperiod, *F*(6, 187.08) = 16.15, *p* < 0.001) and main effects of both treatment (*F*(2, 85.82) = 4.28, *p* = 0.017) and photoperiod (*F*(1, 149.93) = 377.93, *p* < 0.001). Overall, 5 mg/kg AM281 decreased REM sleep (*t*(89.77) = -2.92, *p* = 0.009), particularly during the third quarter of the DP (ZT18-21: *t*(241.64) = -2.88, *p* = 0.009). REM bout duration was affected by an overall interaction (treatment x time of day within photoperiod, *F*(12, 176.73) = 2.60, *p* = 0.003), nested interaction (time of day within photoperiod, *F*(6, 169.25) = 6.37, *p* < 0.001), and main effect of photoperiod (*F*(1, 151.57) = 24.16, *p* < 0.001). The 0.5 mg/kg dose of AM281 increased REM bout duration during the second quarter of the DP (ZT15-18: *t*(179.07) = 3.04, *p* = 0.005). There was no effect of AM281 on the number of REM bouts.

When AM281 was administered prior to the LP, overall sleep time did not change substantially, but there was a profound fragmentation of NREM (Fig 10C, top row). For NREM sleep time, there was an overall interaction (treatment x time of day within photoperiod, *F*(18, 146.08) = 8.76, *p* < 0.001), a secondary interaction (treatment x photoperiod, *F*(2, 102.87) = 3.31, *p* = 0.040) and main effects of both treatment (F(2, 81.59) = 3.97, *p* = 0.023) and photoperiod (*F*(1, 122.15) = 383.92, *p* < 0.001). When delivered at the onset of the LP, AM281 increased overall NREM sleep time (*t*(84.01) = 2.74, *p* = 0.015), but a comparison between photoperiods found that NREM sleep time was only increased during the DP (low dose: *t*(91.65) = 2.38, *p* = 0.039; high dose: *t*(91.65) = 2.84, *p* = 0.011). More specifically, there was a significant increase in NREM following high dose AM281 during the first 3 Hr of the DP (ZT12-15: *t*(191.99) = 3.15, *p* = 0.004), which was the same point in the circadian cycle when NREM sleep time was increased in the experiment where AM281 was delivered before the DP. For NREM bout duration, there was an overall interaction (treatment x time of day within photoperiod, *F*(18, 141.98) = 4.74, *p* < 0.001), a secondary interaction (treatment x photoperiod, *F*(2, 82.68) = 12.61, *p* < 0.001), and main effects of both treatment (*F*(2, 59.88) = 9.86, *p* < 0.001) and photoperiod (*F*(1, 109.96) = 31.83, *p* < 0.001). High dose AM281 substantially reduced NREM bout duration (*t*(62.07) = -4.42, *p* < 0.001), particularly during the LP (*t*(69.68) = -6.23, *p* < 0.001). More specifically, NREM bout duration was reduced for the first 3 Hr of the LP following low dose AM281 (ZT00-03: *t*(176.12) = -2.82, *p* = 0.011) and for the first 9 Hr following high dose AM281 (ZT00-09: *t*(176.12) ≤ -3.46, *p* ≤ 0.001). The number of NREM bouts was affected in the opposite manner. There was an overall interaction (treatment x time of day within photoperiod, *F*(18, 144.093) = 3.266, *p* < 0.001), a secondary interaction (treatment x photoperiod, *F*(2, 78.77) = 13.65, *p* < 0.001), and main effects of both treatment (*F*(2, 53.14) = 19.99, *p* < 0.001) and photoperiod (F(1, 113.94) = 148.145, *p* < 0.001). High dose AM281 increased the number of NREM bouts (*t*(63.61) = 6.79, *p* < 0.001) particularly during the first 9 Hr of the LP (ZT00-09: *t*(159.32) ≥ 4.22, *p* < 0.001), and both doses increased the number of NREM bouts during the second quarter of the DP (ZT15-18: *t*(159.32) ≥ 2.31, *p* ≤ 0.045). Thus, blockade of CB1 receptors greatly fragments NREM sleep, but opposing effects on NREM bout duration and the number of NREM bouts result in subtle changes in total sleep time.

In addition to the effects on NREM sleep, REM sleep time was significantly reduced following AM281 administration prior to the LP (Fig 10C, bottom row). For the percent of time spent in REM sleep, there was a secondary interaction (treatment x photoperiod, *F*(2, 95.51) = 36.30, *p* < 0.001), nested interaction (time of day within photoperiod, *F*(6, 143.275) = 11.15, *p* < 0.001), and main effects of both treatment (*F*(2, 74.2) = 21.38, *p* < 0.001) and photoperiod (*F*(1, 116.42) = 68.40, *p* < 0.001). Both low and high dose AM281 reduced REM sleep during the LP (*t*(84.01) ≤ -4.22, *p* < 0.001). Low dose AM281 reduced REM sleep during the first 6 Hr of the LP (ZT00-06: *t*(190.28) ≤ -3.30, *p* ≤ 0.002), and high dose AM281 reduced REM at all times during the LP (ZT00-12: *t*(190.28) ≤ -3.40, *p* ≤ 0.002). For REM bout duration, there was a secondary interaction (treatment x photoperiod, *F*(2, 81.71) = 7.10, *p* = 0.001), nested interaction (time of day within photoperiod, *F*(6, 139.18) = 5.34, *p* < 0.001), and a main effect of treatment (*F*(2, 56.07) = 3.35, *p* = 0.042). The high dose of AM281 reduced REM bout duration during the LP (*t*(64.52) = -4.05, *p* < 0.001), particularly during the first 9 Hr of the LP (ZT00-09: *t*(156.05) ≤ -2.31, *p* ≤ 0.044). For the number of REM bouts, there was an overall interaction (treatment x time of day within photoperiod, *F*(12, 133.54) = 1.85, *p* = 0.046), secondary interaction (treatment x photoperiod, *F*(2, 68.89) 7.463, *p* = 0.001), nested interaction (time of day within photoperiod, *F*(6, 127.15) = 3.99, *p* = 0.001), and main effects of both treatment (*F*(2, 46.46) = 6.39, *p* = 0.004) and photoperiod (*F*(1, 99.89) = 59.86, *p* < 0.001). The number of REM bouts were reduced by high dose AM281 during the LP (*t*(54.99) = -4.49, *p* < 0.001) and increased by low dose AM281 during the DP (*t*(54.65) = 2.44, *p* = 0.036). At the high dose, AM281 reduced the number of REM bouts across most of the LP (ZT00-06 & 09–12: *t*(162.69) ≤ -2.67, *p* = 0.002), while low dose AM281 decreased the number of REM bouts during the second quarter of the LP (ZT03-06: *t*(162.62) = -3.30, *p* = 0.002) and increased REM bouts during the third quarter of the DP (ZT18-21: *t*(157.68) = 2.51, *p* = 0.026). Thus, blockade of CB1 signaling fragments NREM and decreases REM sleep, suggesting that this receptor is necessary for NREM stability.

#### EEG Power Spectral Measurements

**Fig 11:**
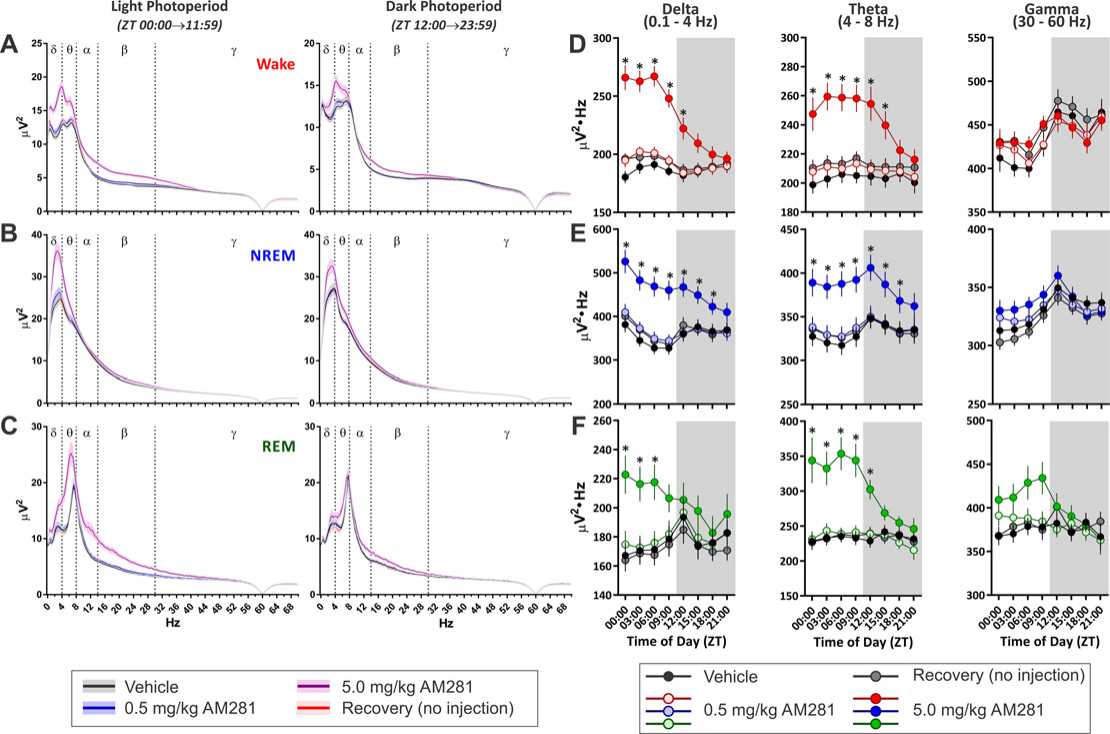
Blockade of CB1 Receptors Produces Broadband Changes in EEG Power Spectral Features. Results are from experiment where AM281 was administered at the onset of the LP. **A—C**, Average of power spectra for each photoperiod and for each vigilance state across different days of the experiment. Solid lines indicate mean of all subjects as a function of frequency, and shaded regions surrounding lines denotes standard error of the mean. **A,** Power spectra from wake epochs. **B**, Power spectra from NREM epochs. **C**, Power spectra from REM epochs. **D–F**, Average power in specified bandwidths in each state for 3 Hr epochs over the day. Data from each vigilance state are color coded with wake in red (**D**), NREM in blue (**E**), and REM in green (**F**). Gray backgrounds indicate the DP. Asterisks (*) denote significant pair-wise comparisons between drug conditions and measures obtained during vehicle baseline. Symbols represent means±SEM across all subjects (*N* = 9) for each 3 Hr time bin.

Given that blockade of endocannabinoid signaling through CB1 fragments NREM sleep, we hypothesized that power spectral features associated with sleep might be disrupted after acute administration of CB1 antagonists. Administration of AM281 before the LP had large effects on power spectral features of the EEG across vigilance states, but the nature of these effects was different across states (Fig 11A–11C). Notably, the power of low frequencies (< 8 Hz) was consistently increased, and high frequencies (gamma, > 30 Hz) were much less affected by CB1 blockade. These effects lasted for most of the day, and a similar time course was observed in experiments where AM281 was administered before the DP (S8 Fig), suggesting that this effect is not modulated by circadian processes.

As for we did for CP47, AM3506, and JZL184 we quantified power spectral bandwidths (delta, theta, and gamma) in 3 Hr time bins over the entire recording (Fig 11D–11F). During wake epochs (Fig 11D) there was a significant interaction (treatment x time of day within photoperiod, *F*(24, 229.63) = 5.26, *p* < 0.001) with main effects of both treatment (*F*(3, 73.99) = 42.19, *p* < 0.001) and photoperiod (*F*(1, 154.27) = 127.39, *p* < 0.001) for delta power. Only the 5.0 mg/kg dose significantly elevated wake delta relative to vehicle (*t*(53.52) = 7.21, *p* < 0.001), and comparisons at individual time points found that this effect lasted for 18 Hr after drug administration (ZT00-18: *t*(83.70) > 3.13, *p* ≤ 0.007). Wake theta power was also modulated by a significant interaction (*F*(24, 228.72) = 3.23, *p* < 0.001) and main effects of both treatment (*F*(3, 69.952) = 20.74, *p* < 0.001) and photoperiod (*F*(1, 157.78) = 13.20, *p* < 0.001). Again, only the high dose of AM281 significantly elevated theta power over the circadian cycle (*t*(50.532) = 5.35, *p* < 0.001), and theta power was increased over the first 18 Hr of the recording period (ZT00-18: *t*(80.45) > 3.97, *p* < 0.001). Analysis of wake gamma power also found an overall interaction (*F*(24, 227.65) = 8.013, *p* < 0.001) with a main effect of photoperiod (*F*(1, 143.48) = 89.70, *p* < 0.001), but no pair-wise comparisons between treatment conditions and the vehicle baseline reached significance.

AM281 also altered EEG power spectra during NREM epochs (Fig 11E). For NREM delta power, there was a significant overall interaction (*F*(24, 229.40) = 9.84, *p* < 0.001) with a main effect of treatment (*F*(3, 80.45) = 28.89, *p* < 0.001). 5 mg/kg AM281 produced an overall increase in NREM delta power (*t*(58.23) = 5.54, *p* < 0.001) with pair-wise differences noted across the vast majority of the recording (ZT00-21: *t*(88.09) ≥ 2.53, *p* ≤ 0.039). There was also an overall interaction for NREM theta power (*F*(24, 235.54) = 6.31, *p* < 0.001) with main effects of both treatment (*F*(3, 148.62) = 35.06, *p* < 0.001) and photoperiod (*F*(1, 135.42) = 5.438, *p* = 0.21). 5 mg/kg AM281 increased the overall power in the theta bandwidth during NREM sleep (*t*(118.00) = 5.01, *p* < 0.001) with specific pair-wise comparisons over the majority of the recording (ZT00-21: *t*(150.13) ≥ 2.86, p ≤ 0.014). Finally, analysis of NREM gamma found an overall interaction (*F*(24, 233.59) = 14.55, *p* < 0.001) with main effects of both treatment (F(3, 121.33) = 7.128, *p* < 0.001) and photoperiod (*F*(1, 127.916) = 93.21, *p* < 0.001), but there were no significant differences between AM281 and vehicle.

The CB1 antagonist also affected EEG spectral content during REM (Fig 11F). For REM delta power, there was an overall interaction (*F*(24, 217.83) = 1.68, *p* = 0.028) with a main effect of treatment (*F*(3, 54.98) = 7.64, *p* < 0.001). 5.0 mg/kg AM281 increased delta power during REM sleep epochs across the day (*t*(40.91) = 2.82, *p* < 0.022), but this was mainly due to increased REM delta during the first 9 Hr (ZT00-09: *t*(76.78) ≥ 3.54, *p* ≤ 0.002). For REM theta power, there was a secondary interaction (treatment x photoperiod, *F*(3, 146.49) = 9.23, *p* < 0.001) with main effects of both treatment (*F*(3, 51.66) = 19.22, *p* < 0.001) and photoperiod (*F*(1, 219.58) = 18.05, *p* < 0.001). Overall, 5.0 mg/kg AM281 increased REM theta power (*t*(39.91) = 5.23, *p* < 0.001), particularly during the first 15 Hr of the recording (ZT00-15: *t*(85.58) ≥ 3.92, p ≤ 0.001). There was a treatment x photoperiod interaction for REM gamma power (*F*(3, 168.32) = 3.61, *p* = 0.015) with a main effect of photoperiod (*F*(1, 196.66) = 8.13, *p* = 0.005). Overall, REM gamma was augmented by the high dose of AM281 during the light photoperiod (*t*(51.71) = 3.12, *p* = 0.009), but there were no differences at specific time points. Thus, blockade of CB1 receptors produces broadband changes in the EEG waveform that are particularly evident in lower frequencies irrespective of vigilance state.

### eCB Signaling is Not Necessary for Sleep Homeostasis but is Required for the Stability of Rebound Sleep

**Fig 12:**
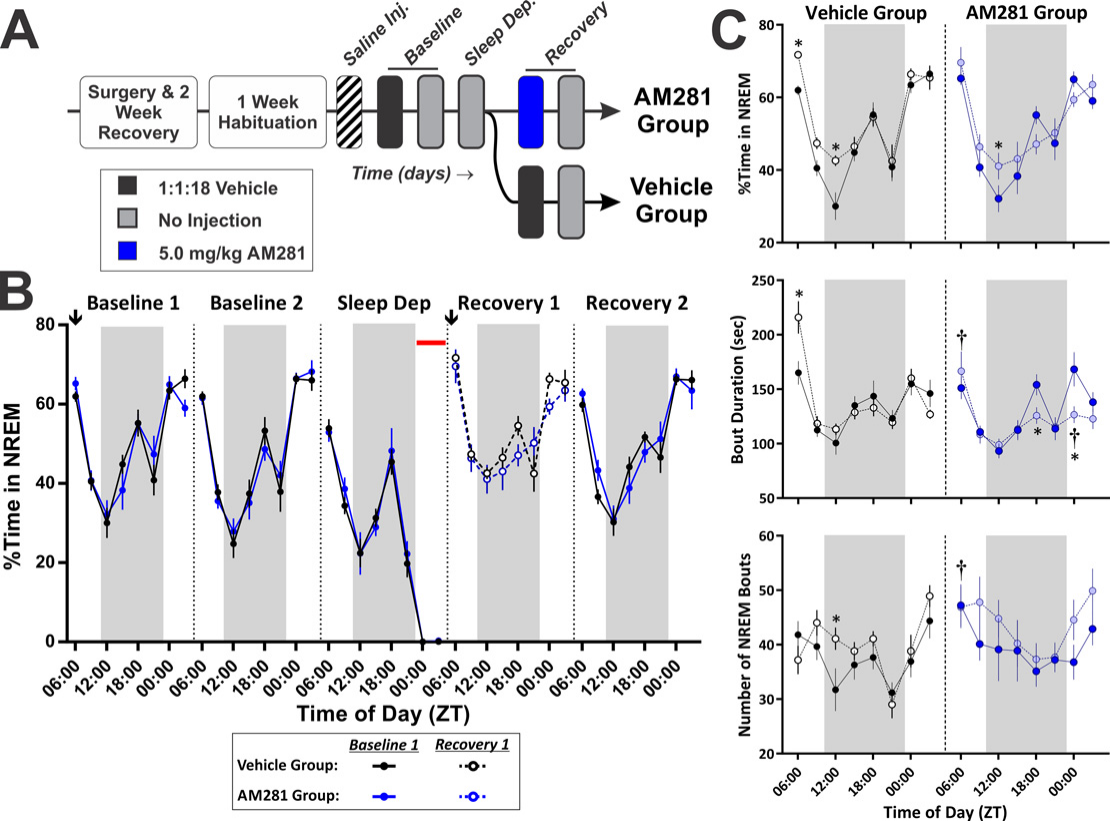
Endocannabinoid Signaling Is Necessary for the Rebound in NREM Duration Following Sleep Deprivation, but Blockade of CB1 Has only a Weak Effect on the Rebound in Total Sleep Time. **A**, Schematic overview of sleep deprivation experimental paradigm. **B**, Time course of NREM sleep time across all days of sleep deprivation experiment. Horizontal red bar on the *Sleep Dep* day indicates when sleep deprivation took place. Vertical dotted lines denote boundaries between experimental phases. Blue symbols/lines show AM281 group (*N* = 9) while the vehicle group (*N* = 11) is depicted in black. Downward facing arrows () indicate time of drug administration. **C**, Comparisons of total NREM sleep time (top), NREM bout duration (middle), and the number of NREM bouts (bottom) on the first baseline day (vehicle administration all groups) and first day of recovery (vehicle or AM281 administration). Asterisks (*) denote significant pair-wise comparisons within-groups between drug conditions and measures obtained during vehicle baseline. Daggers (**†**) denote significant pair-wise comparisons between groups on the recovery day. In **B** & **C**, Grey shaded regions indicate the DP, and symbols/bars represent means±SEM across all subjects for each 3 Hr time bin.

Measures of EEG delta and theta power are frequently used as an index of sleep drive [44, 50, 51], and according to this interpretation, the large increase in NREM delta power following administration of AM281 may be indicative of augmented sleep homeostatic drive. Thus, we sought to test this possibility by administering AM281 following 6 Hr of total sleep deprivation (TSD) using a rotating disc paradigm (Fig 12A & S1 Fig). Measurements of sleep during the deprivation period clearly show that this method was successful in achieving TSD for 6 Hr (Fig 12B).

Examination of the percent of time subjects spent in NREM sleep (Fig 12C, top) found an overall interaction (group x time of day within photoperiod within experimental phase, *F*(24, 213.06) = 10.34, *p* < 0.001) with main effects of experimental phase (*F*(1, 112,24) = 8.26, *p* < 0.005) and photoperiod (*F*(1, 165.42) = 187.28, *p* < 0.001). In general, subjects engaged in more NREM sleep following sleep deprivation (*t*(112.24) = 2.88, *p* = 0.005), indicating a significant NREM rebound. Compared to baseline, the vehicle group spent significantly more time in NREM during the first 3 Hr of recovery (ZT06-09: *t*(286.99) = 2.40, *p* = 0.017) and during the first 3 Hr of the DP (ZT12-15: *t*(286.99) = 3.08, *p* = 0.002). Similarly, the AM281 group had increased NREM sleep during the first 3 Hr of the DP (ZT12-15: *t*(286.99) = 1.99, *p* = 0.47). There were no differences in the amount of NREM sleep between the vehicle and AM281 treatment groups during either baseline or recovery. Additionally, there was no difference between treatment groups in the recovery of the sleep deficit induced by the 6 Hr TSD protocol (S9 Fig). Thus, both groups displayed an augmentation of NREM sleep time following 6 Hr sleep deprivation, and the two groups did not differ in regards to the total amount of sleep obtained during recovery from sleep deprivation.

In contrast, there were significant between-group differences for NREM architecture during recovery from sleep deprivation. For NREM bout duration (stability; Fig 12C, middle), there was an overall interaction (group x time of day within photoperiod within experimental phase, *F*(24, 213.74) = 8.66, *p* < 0.001) with main effects of treatment group (*F*(1, 76.81) = 7.14, *p* = 0.009) and photoperiod (*F*(1, 158.63) = 37.26, *p* < 0.001). The AM281 group had reduced NREM stability compared to the vehicle group (*t*(76.81) = -2.67, *p* = 0.009), with specific differences evident at two time points of recovery (ZT06-09: *t*(256.30) = -3.63, *p* < 0.001; ZT00-03: *t*(256.30) = -2.47, *p* = 0.014). There were no between-group differences during the baseline recordings. Relative to their own baseline, the vehicle group had increased NREM stability during the first 3 Hr of recovery (ZT06-09: *t*(265.32) = 3.95, *p* < 0.001), but the AM281 treated mice did not show increased NREM stability following TSD. Rather, the AM281 group had decreased NREM stability during the second half of the recording (ZT18-21 & 00–03: *t*(265.32) ≤ -1.99, *p* ≤ 0.048). For the number of NREM bouts (Fig 12C, bottom), there was an overall interaction (group x time of day within photoperiod within experimental phase, *F*(24, 220.93) = 2.95, *p* < 0.001) and a main effect of photoperiod (*F*(1, 174.17) = 17.77, *p* < 0.001). There were no differences between groups during baseline, but the AM281 group had significantly more NREM bouts than the vehicle group during the first three hours of recovery (ZT06-09: *t*(169.85) = 2.16, *p* = 0.032). Relative to their own baseline, the vehicle group increased the number of NREM bouts during the first quarter of the DP (ZT12-15: *t*(177.13) = 2.20, *p* = 0.029). These findings show that AM281 blocks the increase in NREM stability during recovery from TSD and increases the number of NREM bouts to compensate.

REM sleep was also affected by TSD with reduced REM early in recovery in both the AM281 and vehicle groups (S10 Fig). Late in the recovery, there was increase in REM sleep in the AM281 treated group due to an increase in the number of REM bouts. Consistent with our previous experiments, blockade of CB1 receptors produced a large increase in low frequency oscillations that occluded any changes due to sleep deprivation (S11 Fig). However, wake theta power did increase during the sleep deprivation period (S11 Fig, panel B). Thus, power spectral results cannot be used as an index of homeostatic drive in this experiment as they are confounded by the baseline effects of CB1 antagonism.

## Discussion

The major findings of this work are that eCB signaling through the CB1 receptor is necessary and sufficient for the stability of NREM sleep. Direct activation of CB1 with CP47 or increasing eCB tone with JZL or AM3506 augmented the time spent in NREM primarily due to increased NREM bout length. This suggests that increasing eCB signaling stabilizes the NREM state. Notably these effects were biphasic, where the initial increase in sleep and NREM stability gave way to a secondary reduction and destabilization of NREM bouts suggesting that homeostatic regulation of sleep remained intact. Further support for the role of eCBs in regulating NREM stability comes from experiments with the CB1 antagonist AM281, where blockade of CB1 reduced the duration of NREM bouts. Importantly, this effect was largely offset by a concomitant increase in the number of NREM bouts resulting in minimal changes in overall sleep time. Again, the opposing effects of reduced NREM bout duration and number suggest homeostatic regulation of sleep remains intact in the absence of CB1 signaling. However, CB1 blockade consistently resulted in substantial broadband changes to EEG power spectra, particularly in low frequency bandwidths that are often used as correlates of sleep drive. To test the involvement of eCB signaling in sleep homeostatic processes, we administered AM281 immediately prior to a recovery from TSD. Blockade of CB1 during recovery from TSD did not occlude the rebound in NREM sleep time following TSD, but it did completely block the increased stability (bout duration) of NREM sleep. Unfortunately, the baseline effects of CB1 antagonism on EEG power spectra hobbled our attempts to use this as a metric of sleep drive. Thus, the major conclusion from this work is that eCBs regulate sleep stability, but eCB signaling is not necessary for sleep homeostasis.

### Validity and Robustness of State-Scoring Algorithm

In order to perform the series of experiments described herein, we needed to find a way to score mouse polysomnographic data in an accurate and timely manner, so we developed and validated the state-space based approach. Our method was heavily influenced by previous reports, but we modified and extended this work, in part by automating the state-assignment process after state-space coordinates have been defined. Discrete fourier analysis is the basis of this approach, because the state-space coordinates are derived from power spectral ratios similar to those used by others [36, 38, 39, 52, 53]. The use of power spectral ratios (effectively normalizing the FFT) to define state-space coordinates, as opposed to raw power spectral values [54], means that our method of state-scoring is robust to changes in the FFT, and this is evidenced by our experimental results. Activating CB1/eCB signaling with CP47, JZL, or AM3506 produced only modest effects on power spectral measurements, but these drugs had large biphasic effects on measures of sleep time. Importantly, the effects of these drugs on sleep and EEG power spectra were not temporally aligned. In contrast, CB1 blockade consistently produced broadband changes in EEG power spectra that were most evident for low frequency bandwidths, and these effects were evident irrespective of the time of day of drug administration or if CB1 was blocked following TSD. However, CB1 antagonism did not produce substantial effects on total sleep time and the magnitude of effects on sleep architecture varied with time of drug administration. Thus, while CB1 antagonism consistently and substantially alters EEG power spectra, the time course of these effects are not aligned with changes in the sleep score or even sleep architecture. Consequently, the various data sets presented here show large changes in sleep in the absence of substantial changes in state-dependent EEG power spectra (direct and indirect CB1 agonists) and large changes in power spectra in the absence of substantive changes in sleep time (CB1 antagonism). Therefore, we conclude that in addition to performing comparably to human scoring, our vigilance state scoring algorithm is robust to cannabinoid pharmacological manipulations that alter EEG power spectra.

### The Biphasic Effects of Augmented eCB Signaling

The early facilitation of NREM sleep seen in the present study following activation of CB1 is largely in agreement with earlier findings [6–11, 13–15, 29, 32], but the secondary (wake promoting/sleep fragmenting) effect of CP47 and JZL to reduce NREM sleep at a time of day when mice normally engage in most of their daily sleep is a novel finding. It should be mentioned that JZL had lower magnitude effects (change from vehicle) on NREM when it was administered before the LP, and did not produce substantial fragmentation of sleep during the subsequent DP. It is unclear why a secondary process was not evident in this experiment, but the acute augmentation of sleep stability was, in this case, synchronized with the normal time of day when mice are inactive. Thus, there was no perturbation in the circadian timing or homeostatic regulation of NREM and wake as in experiments where JZL, CP47, and AM3506 were administered before the DP.

Based on several observations, we hypothesize that the sleep fragmentation during the secondary phase of CP47, JZL, and AM3506 effects is due to a rapid reduction in CB1 signaling. First, JZL selectively increases 2-AG levels via inhibition of MAGL, and both CP47 and 2-AG are highly potent, full agonists at the CB1 receptor [55–57]. In contrast, as an irreversible inhibitor of FAAH, AM3506 increases *N*-acylethanolamine transmitters for several days [49], and AEA is only a weakly efficacious agonist at CB1 [58]. This discrepancy could explain the difference in the magnitude of effects seen between these drugs. On the other hand, the lack of any effect of URB597 even during the first few hours of the recording is surprising, and it is possible that the early induction of sleep by AM3506 could be due to off-target augmentation of 2-AG. However, administration of exogenous AEA increases NREM sleep [13, 14], and administration of THC, a partial agonist at CB1, also increases NREM sleep (unpublished observation). Thus, partial activation of CB1 with endogenous *N*-acylethanolamines is likely to have hypnogenic effects. Second, following acute activation, CB1 receptors are rapidly down-regulated on a time scale compatible with the biphasic effects of CP47 and JZL [59, 60]. As these compounds are slowly eliminated in the hours following injection, receptor function will be decreased compared to baseline. Although data on diurnal expression of CB1 are extremely limited, a few studies have provided evidence that CB1 accumulates in brain and neuroendocrine tissue during the DP [61, 62]. Thus, it is possible that diurnal fluctuations in CB1 expression could account for the blunted biphasic effects seen with JZL administration before the LP. Third, NREM fragmentation was also observed in the present study following administration of CB1 antagonists, and CB1 knockout mice also have fragmented sleep [24, 25].

### On the Timing of Drug Administration

Although CB1 blockade consistently reduced NREM bout duration, the effects were greatest when AM281 was administered at the onset of the LP. Similarly, JZL had differential effects on sleep depending on the circadian timing of drug administration. When JZL was administered either before the LP or the DP, it increased NREM sleep time and bout duration, but the magnitude of these effects was blunted when the drug was given before the LP. Consequently, the timing of maximal effects for activation and inhibition of eCB signaling peaks at opposite poles of the circadian cycle, suggesting that eCB signaling interacts with or is controlled by circadian processes. These are not mutually exclusive possibilities, and there is evidence supporting both mechanisms. First, cannabinoids alter the light-induced phase-shift in activity in free running rodents [63, 64], suggesting that CB1 can influence entrainment to photic stimulation. Additionally, eCB levels fluctuate over the circadian period in a brain-region dependent manner [33, 34], suggesting that eCB signaling may be under circadian regulation. In fact, levels of AEA and 2-AG are lowest in cortical and hypothalamic tissues at the onset of the DP [33], and in the present work, administration of CB1 antagonists at this time point produced smaller effects on sleep architecture. Thus, our findings provide evidence that changing levels of the molecular components of the eCB system have functional consequences for eCB regulation of vigilance states.

### eCBs and EEG Power Spectra

NREM delta power has been used as an index for sleep homeostatic drive because of the correlation of this measure with the difference in cumulative time awake versus asleep [44, 50, 51]. However, as Davis et al [65] point out, some pharmacological manipulations can dissociate alterations in delta power and changes in vigilance states, thus confounding NREM delta power as a metric of sleep homeostatic drive. This situation is clearly applicable to the current dataset, where CB1 antagonists increased delta power across all vigilance states without changing total sleep time. On the contrary, CB1 antagonists fragment NREM sleep, and this may lead one to speculate that the augmentation of delta is a response to poor sleep quality, and therefore, an increase in sleep drive. In fact, sleep fragmentation protocols are associated with increasing homeostatic sleep drive [66–68]. However, evidence from the current dataset argues against the hypothesis that the CB1 antagonist-induced increase in delta power is indicative of an increase in sleep homeostatic processes. First, the time course for augmented delta does not reflect a gradual increase that one would expect if homeostatic sleep drive were building over time with reduced sleep quality. Second, the rebound in total NREM sleep following 6 Hr sleep deprivation was not occluded by CB1 antagonists. Third, blockade of CB1 did not alter the rate of recovery from sleep deprivation.

It should be noted that we did not normalize our power spectral data as in other reports [17, 28, 29, 32], and our presentation of raw power spectral results may explain some discrepancies between our findings and those of others. It is not clear why other researchers have not observed the large augmentation of delta and theta power following administration of CB1 antagonists reported here. In fact, several reports have observed reduced delta and theta power following administration of CB1 antagonists [19, 28] while others have concluded that these drugs have little or no effect on the EEG power spectrum [29, 32]. We can only speculate that the variability in methods used to normalize power spectral bandwidths has contributed to these inconsistencies.

Although we observed no changes in low-frequency oscillations following drugs that activate eCB signaling (JZL, AM3506, CP47), gamma oscillations were consistently reduced, particularly during NREM and REM sleep. This result is consistent with previous reports suggesting reduced gamma power is a robust effect of increased CB1 activation [69–73]. Another consistent effect of cannabinoid agonists is to facilitate high-voltage spindles/spike wave discharges (HVS) [74–76], but in the present study, power spectral analysis found no changes in the HVS bandwidth. This is not surprising given that spindles are rare in C57 mice under baseline conditions [77, 78], so at 3–12 Hr time bins used here, spindle events would only account for a small fraction of the NREM epochs. Thus, it is highly likely that any changes would be averaged out with the analysis performed here. In all cases where cannabinoids have been reported to augment spindles and their respective power spectral correlates [74–76], some prior detection method has been implemented to isolate epochs containing these events before comparing power spectral features across drug conditions. Consequently, no conclusions regarding cannabinoid-induced changes in the incidence of spindles can be derived from the present results.

### eCB Signaling and Sleep

Previous reports of CB1 antagonist effects in animal studies of sleep indicated either subtle augmentation of wake at the expense of NREM [15, 19, 28, 29] or no effect [13, 31, 32]. The subtle differences observed with respect to CB1 antagonist effects on sleep time across these studies are likely due to different doses and times of administration. Additionally, most of these studies used relatively short recordings (4–8 Hr). In the reports indicating increased wake time following administration of CB1 antagonists, the arousing properties of these drugs were only observed when summating across the entire recording. On the other hand, fragmentation of NREM has not been reported, but only a few studies have examined metrics of sleep architecture following administration of CB1 antagonists [29, 32]. Studies in CB1 KO mice have found significantly reduced NREM bout duration with an increased number of NREM bouts [24, 25]. REM sleep is consistently reduced following administration of CB1 antagonists [28, 29, 32, 79, 80], and reduced REM sleep is frequently associated with NREM fragmentation [67, 68]. In the present study, reductions in REM sleep time were associated with time points where there was noticeable fragmentation of NREM. Thus, it is likely that NREM fragmentation is a common outcome of CB1 antagonism, and this may give rise to reduced REM.

Importantly, a large reduction in REM sleep was seen following JZL administration before the LP. This finding was not as evident when this drug was administered prior to the DP, likely due to a floor effect as REM is very infrequent the DP. However, some impairment of REM was seen in all experiments in the present study with eCB/CB1 system activation and by others with THC administration [81], suggesting that suppression of REM is a consistent effect of globally increasing eCB signaling. Nevertheless, the similarity between the effect of JZL and AM281 to reduce REM is striking. In contrast to the NREM fragmentation evoked by AM281, facilitating eCB/CB1 signaling stabilizes NREM sleep, and the similar suppression of REM seen with these two opposite manipulations indicates that an optimal level of NREM stabilization must be achieved to allow for REM to emerge. On the neurobiological level, it is possible that the REM suppressing effects of CB1 antagonism and activation arise from different circuit elements controlling vigilance states. This would not be surprising for the eCB system, considering its molecular constituents are widespread throughout structures known to control sleep-wake transitions and considering CB1-activation reduces neurotransmission at both excitatory and inhibitory synapses. However, all drug administrations were performed systemically, so no specific conclusions about neurobiological loci can be drawn from the results presented herein.

According to the influential two-process model, sleep timing is regulated by interactions between homeostatic and circadian processes, where homeostatic control of sleep is defined as a mechanism that increases the propensity to sleep as a function of the amount of time spent awake [44, 51]. Changes in the amount of sleep arise from modulation of one of these two processes. Our data do not support a role for eCB signaling in sleep homeostasis. First, CB1 activation causes a biphasic response in NREM sleep time, increasing sleep during the DP and reducing it during the LP. This reduction in sleep time during the LP (secondary response) is the expected homeostatic response to increased sleep during the DP. Second, blockade of CB1 signaling does not substantially alter sleep time. While the duration of NREM bouts is reduced by CB1 antagonism, the compensatory increase in the number of bouts argues for an intact homeostatic mechanism. Finally, there was not a robust effect of CB1 blockade on rebound sleep time or the amount of sleep recovered following TSD. Consequently, we conclude that it is unlikely that eCB signaling is an essential component of sleep homeostatic machinery.

### Relevance and Future Directions

In both rodents and humans, chronic cannabinoid administration produces down-regulation of CB1 signaling [82, 83], and although sleep disturbances are a central feature of cannabis withdrawal in humans [84], there is marked paucity of objective studies describing exactly how sleep is disrupted much less a mechanism for these disruptions [85]. Interestingly, insomnia and poor sleep quality were commonly reported adverse effects in clinical trials with the CB1 antagonist rimonabant, but again, no objective measures of sleep were obtained [22, 23]. The rimonabant trial and trials with other CB1 antagonists were terminated due to the increased depression, anxiety, and suicidality that was associated with these drugs, so it is unlikely that any data pertaining to how CB1 antagonists affect human sleep will be forthcoming in the near future. Nevertheless, the self-reports of sleep disturbances from these clinical trials are interesting considering the association between insomnia and depression [86]. Our findings demonstrate that eCB signaling is necessary and sufficient for the control of sleep stability, but this neurotransmitter system is not necessary for sleep homeostasis.

## Supporting Information

S1 Data Zip File Containing Data Relevant to This Manuscript. (ZIP)

S1 Fig Sleep Deprivation Apparatus. **A**, Exploded schematic view of structural components of the sleep deprivation chambers labelled with dimensions in inches. **B**, Photograph of an assembled device and a schematic of a top down view of the chamber. Note that the rotation described in the schematic implies rotation of the chamber floor/disc suspended beneath the clear acrylic chamber wall. Also note the commutator and tether in the photograph. Polysomnographic activity can be recorded in these chambers during the sleep deprivation. **C,** Schematic overview of assembled system including electronic control components: break-our board (BoB), Arduino, TTL controlled relay to control power circuit to motors, voltage regulator, and computer running custom control software written in MATLAB.
(PDF)

S2 Fig Vehicle Solution Does Not Alter Sleep Parameters in C57BL/6J Mice. Data are from experiment with CP47 (*N* = 9), where subjects were administered a saline injection i.p. the day prior to the vehicle injection. The vehicle data depicted here are the same as those depicted in Fig 4. **A,** NREM sleep time or architecture. Top graph: Percent time in NREM was not affected by the vehicle solution. Middle graph: The duration of NREM bouts was not affected by vehicle injection. Bottom graph: The number of NREM bouts was not affected by the vehicle solution. **B**, REM sleep time and architecture. Top graph: The percent time in REM was not affected by vehicle injection. Middle graph: The duration of REM bouts was not affected by vehicle injection. Bottom graph: For the number of REM bouts, there was an overall interaction (treatment x time of day within photoperiod, *F*(6,98.77) = 2.63, *p* = 0.021), nested interaction (time of day within photoperiod, *F*(6, 95.49) = 6.56, *p* < 0.001), and a main effect of treatment (*F*(1, 74.92) = 82.37, *p* < 0.001). Overall, the vehicle solution did not alter the number of REM bouts when data were collapsed across the day or when comparisons were made with data collapsed within either LP or DP. However, there was a slight reduction in the number of REM bouts at one point in the LP (ZT06-09: *t*(82.02) = -2.10, *p* = 0.039). Given the small effect size, limited to only one measure of REM architecture in a very restricted timeframe many hours after the injection, we conclude that the vehicle solution used in this study has little or no effect on sleep in C57BL/6 mice. **C-E**, There were no obvious changes in EEG power spectra following vehicle administration. **C**, Power spectra from wake epochs. **D,** Power spectra from NREM epochs. **E**, Power spectra from REM epochs. In **A** & **B**, Grey shaded regions indicate the DP, and symbols/bars represent means±SEM across all subjects for each 3 Hr time bin.
(PDF)

S3 Fig Percent Agreement Between Automated and Human Scoring of Data by Vigilance State. To compute percent agreement by vigilance state, each human’s score and the computer’s score were compared against a template derived from human scored data. This meant that for each human there were two possible templates, and these values were averaged together yielding one human:human percent agreement score per human scorer per each of 5 data files used (the data file with corrupt EMG channel used in overall percent agreement, Fig 1C, was excluded for this analysis as it was unscorable). Thus, for each data file there were three human:human measures and three computer:human measures. The state-specific percent agreement was calculated as the fraction of epochs where the scorer and template agreed that epochs were or were not a target state over the total number of epochs (% agreement = 100% x [agree State + agree not State]/total number of epochs). For each state (wake, NREM, and REM) a two-way repeated measures ANOVA was performed with data file as a repeated factor and scoring comparison (human:human vs. computer:human) as a between-groups factor. **A**, Results for percent agreement for wake epochs. There was an interaction between scoring type and datafile (*F*(4,16) = 3.82, *p* = 0.023) and a main effect of data file (*F*(4,16) = 21.92, *p* < 0.001). However, there was a only a slight reduction in percent agreement for data file number 5 in the human:computer (*t*(20) = 4.14, *p* = 0.003). **B**, Results for percent agreement for NREM epochs. There was only a main effect of data file (*F*(4, 16) = 28.53, *p* < 0.001). **C,** Results for percent agreement for REM epochs. There was only a main effect of data file (*F*(4, 16) = 33.18, *p* < 0.001). **D,** Shows data collapsed across scorers and the results of a paired comparison by data file. Grey, connected points superimposed on the bar graph indicate mean human:human and computer:human agreement for each data file. A paired t-test was performed for each vigilance state. Percent agreement was not significantly different for human:human vs human:computer scored data for wake (*t*(4) = 1.94, *p* = 0.12), NREM (*t*(4) = 1.09, *p* 0.34), or REM (*t*(4) = 0.77, *p* = 0.48).
(PDF)

S4 Fig MAGL Inhibition with JZL184 Administration Before the LP Attenuates Gamma Frequency Oscillations During Sleep. **A-C**, Average power spectra for epochs of different vigilance states across the entire LP (left hand) and DP (right hand). Solid lines denote means and shaded region around lines denotes SEM. **A**, Wake. **B**, NREM. **C**, REM. **D-F**, Change over the day in summated power in different frequency bandwidths from the power spectra: delta (left hand column), theta (middle column), and gamma (right hand column). **D, Wake epochs**. Left panel: For wake delta, there was an overall interaction (treatment x time of day within photoperiod, *F*(24, 261.30) = 2.08, *p* = 0.003), nested interaction (time of day within photoperiod, *F*(6, 242.10) = 9.33, *p* < 0.001), and a main effect of photoperiod (*F*(1, 134.75) = 6.88, *p* = 0.010). The only time point that significantly deviated from vehicle was during the first 3 Hr of the recovery day, when there was an increase in delta power (*t*(215.67) = 2.86, *p* = 0.018). Middle panel: No effect of JZL184 on wake theta power. Right panel: For wake gamma power, there was a nested interaction (time of day within photoperiod, *F*(6, 253.19) = 6.08, *p* < 0.001) and main effects of both treatment (*F*(4,67.43) = 3.21, *p* = 0.018) and photoperiod (*F*(1, 179.51) = 115.90, *p* < 0.001). Specifically, 16 mg/kg JZL reduced gamma power during the first 3 Hr of the LP (ZT 00–03: *t*(57.18) = -2.68, *p* = 0.038). **E, NREM epochs**. Left panel: For NREM delta power, there was no effect of JZL treatment. Middle panel: For NREM theta power, there was an overall interaction (treatment x time of day within photoperiod, *F*(24, 268.23) = 1.64, *p* = 0.033), a nested interaction (time of day within photoperiod, *F*(6, 238.31) = 20.36, *p* < 0.001), and a main effect of photoperiod (*F*(1, 159.84) = 85.90, *p* < 0.001). However, there were no specific time points where JZL184 significantly altered NREM theta power relative to vehicle. Right panel: For NREM gamma power, there was an overall interaction (treatment x time of day within photoperiod, *F*(24, 267.36) = 2.46, *p* < 0.001), a nested interaction (time of day within photoperiod, *F*(6, 234.97) = 31.18, *p* < 0.001), and main effects of both treatment (*F*(4, 163.42) = 22.79, *p* < 0.001) and photoperiod (*F*(1, 126.56) = 230.95, *p* < 0.001). Overall, JZL had dose-dependent effects on NREM gamma, with decreases observed following 8.0 (*t*(145.58) = -3.45, *p* = 0.003) and 16.0 mg/kg doses (*t*(94.93) = -4.97, *p* < 0.001). 8.0 mg/kg JZL decreased NREM gamma power for 18 Hr following drug administration (ZT 00–18: *t*(179.23) ≤ -2.71, *p* ≤ 0.030), and 16.0 mg/kg JZL reduced NREM gamma across the entire recording (ZT 00–00: *t*(116.56) ≤ -2.97, *p* ≤ 0.015). **F, REM epochs**. Left panel: JZL had no effect on REM delta power. Middle panel: JZL had no effect on REM theta power. Right panel: For REM gamma, there was an overall interaction (treatment x time of day within photoperiod, *F*(24, 236.78) = 1.81, *p* = 0.014), a secondary interaction (treatment x photoperiod, *F*(4, 235.193) = 4.82, *p* = 0.001), a nested interaction (time of day within photoperiod, *F*(6, 217.40) = 15.30, *p* < 0.001), and main effects of both treatment (*F*(4, 96.44) = 14.58, *p* < 0.001) and photoperiod (*F*(1, 121.40) = 77.38, *p* < 0.001). There was an overall dose-dependent effect of JZL on REM gamma power, with reduced gamma following both 8.0 (*t*(80.55) = -2.97, *p* = 0.015) and 16.0 mg/kg doses (*t*(49.88) = -4.32, *p* < 0.001). The 8.0 mg/kg dose reduced REM gamma across all time points in the first 15 Hr following administration (ZT 00–15: *t*(113.90) ≤ -2.58, *p* ≤ 0.044), while the 16.0 mg/kg dose decreased gamma power for 18 Hr after administration (ZT 00–18: *t*(67.41) ≤ -3.12, *p* ≤ 0.011). Symbols/Bars represent mean±SEM for 3 hr time bins (*N* = 8). Grey background in graphs shows dark photoperiod. Asterisks denote significant difference from vehicle baseline. All injections administered at onset of LP (ZT 00:00).
(PDF)

S5 Fig Direct Activation of CB1 with CP47,497 Attenuates Gamma Frequency Oscillations During Sleep. **A-C,** Average power spectra for epochs of different vigilance states across the entire DP (left hand) and LP (right hand). Solid lines denote means and shaded region around lines denotes SEM. **A,** Wake. **B,** NREM. **C,** REM. **D-F,** Change over the day in summated power in different frequency bandwidths from the power spectra: delta (left hand column), theta (middle column), and gamma (right hand column). **D, Wake Epochs**. Left panel: CP47 had no effect on wake delta power. Middle panel: For wake theta power, there was a significant overall interaction (drug x time of day within photoperiod, *F*(15, 180.83) = 9.73, *p* < 0.001) with a significant reduction in wake theta at only a single time point during the dark photoperiod (ZT 15–18: *t*(186.39) = -2.29, *p* = 0.047). Right panel: For wake gamma, there was a significant overall interaction (drug x time of day within photoperiod, *F*(15, 179.99) = 3.04, *p* < 0.001) with a main effect of photoperiod (*F*(1, 135.02) = 6.86, *p* = 0.010). However, there was not a difference at any specific time point between low or high dose CP47 and vehicle. **E, NREM Epochs.** Left panel: For NREM delta there was an overall interaction (drug x time of day within photoperiod, *F*(15, 179.22) = 3.07, *p* < 0.001). However, there were no pair-wise differences at any time point between high or low dose CP47 and vehicle. Middle panel: For NREM theta power, there was an overall interaction (*F*(15, 180.85) = 2.79, *p* = 0.001) with main effect of photoperiod (*F*(1, 157.14) = 50.99, *p* < 0.001). However, there were no pair-wise difference between drug treatment conditions and vehicle. Right panel: For NREM gamma, there was an overall interaction (drug x time of day within photoperiod, *F*(15, 181.48) = 3.50, *p* < 0.001), secondary interaction (drug x photoperiod, *F*(2, 184.76) = 8.82, *p* < 0.001) with main effects of drug treatment (*F*(2, 175.98) = 7.13, *p* < 0.001) and photoperiod (*F*(1, 174.89) = 11.39, *p* = 0.001). Specifically, 1.0 mg/kg CP47 reduced NREM gamma power during the first 9 Hr of the dark photoperiod (ZT12-21: *t*(191.79) = -2.38, *p* = 0.037). **F, REM Epochs.** Left panel: There was no effect of CP47 treatment on REM delta power. Middle panel: For REM theta power, there was an overall interaction (drug x time of day within photoperiod, *F*(15, 164.20) = 2.55, *p* = 0.002). High dose CP47 reduced REM theta power only during the first 3 Hr of recording (ZT12-15: *t*(150.16) = 3.14, *p* = 0.004). Right panel: For REM gamma, there was an overall interaction (drug x time of day within photoperiod, *F*(15, 167.92) = 6.02, *p* < 0.001) with a main effect of photoperiod (*F*(1, 134.36) = 14.73, *p* < 0.001). Specifically, high dose CP47 reduced REM gamma power during the first 6 Hr of the dark photoperiod (ZT 12–18: *t*(172.91) = -3.20, *p* ≤ 0.003). Symbols/Bars represent mean±SEM for 3 hr time bins (*N* = 9). Grey background in graphs shows dark photoperiod. Asterisks denote significant difference from vehicle baseline. All injections administered at onset of DP (ZT 12:00).
(PDF)

S6 Fig URB597 Does Not Produce Substantial Effects on Sleep When Administered Systemically. **A,** Diagram of experimental protocol for recording sleep after administration of the reversible FAAH inhibitor, URB597. **B,** Effect of URB597 on NREM sleep time and architecture. Left Graph: There was no effect of URB on NREM sleep time. Middle Graph: For NREM bout duration, there was a nested interaction (time of day within photoperiod, *F*(22,609.62) = 3.04, *p* < 0.001) and main effects of treatment (*F*(4, 312.20) = 56.45, *p* < 0.001) and photoperiod (*F*(1,365.61) = 80.16, *p* < 0.001). 10.0 mg/kg URB produced and overall increase in NREM bout duration (*t*(303.24) = 3.40, *p* = 0.003), specifically during the third hour of the DP (ZT14-15: *t*(1002.87) = 2.82, *p* = 0.020). Right Graph: For the number of NREM bouts, there was a nested interaction (time of day within photoperiod, *F*(22,690.70) = 1.60, *p* = 0.041) and main effects of both treatment (*F*(4,198.00) = 2.97, *p* = 0.021) and photoperiod (*F*(1,253.69) = 88.28, *p* < 0.001). Overall, 10.0 mg/kg URB reduced the number of NREM bouts (*t*(194.74) = -2.84, *p* = 0.020), but there were no differences at specific time points. **C,** Effect of URB597 of REM sleep time and architecture. Left Graph: There was no effect of URB on REM sleep time. Middle Graph: For REM bout duration, there was a nested interaction (time of day within photoperiod, *F*(22,478.80) = 2.31, *p* = 0.001) and main effects of drug treatment (*F*(4,249.61) = 3.80, *p* = 0.005) and photoperiod (*F*(1,302.99) = 11.14, *p* = 0.001). Overall, 10.0 mg/kg URB increased REM bout duration, specifically during the fifth hour of the DP (ZT16-17: *t*(726.45) = 2.54, *p* = 0.045). Right Graph: For the number of REM bouts, there was a nested interaction (time of day within photoperiod, *F*(22,266.50) = 4.84, *p* = 0.001), and main effects of treatment (*F*(4, 266.50) = 4.84, *p* = 0.001) and photoperiod (*F*(1,318.73) = 152.30, *p* < 0.001). Overall, 10.0 mg/kg URB reduced the number of REM bouts (*t*(263.14) = -2.90, *p* = 0.016), but there were several specific time points throughout the day when 10.0 mg/kg URB decreased the number of REM bouts (ZT17-18, 04–05, 09–10: *t*(955.91) ≤ -2.54, *p* ≤ 0.045). Symbols/Bars represent mean±SEM for 1 Hr time bins (*N* = 10). Grey background in graphs shows dark photoperiod. Asterisks denote significant difference from vehicle baseline.
(PDF)

S7 Fig Long-lasting FAAH Inhibition with AM3506 Attenuates Gamma Frequency Oscillations During Sleep. **A-C**, Average power spectra for epochs of different vigilance states across the entire DP (left hand) and LP (right hand). Solid lines denote means and shaded region around lines denotes SEM. **A**, Wake. **B**, NREM. **C**, REM. **D-F**, Change over the day in summated power in different frequency bandwidths from the power spectra: delta (left hand column), theta (middle column), and gamma (right hand column). **D, Wake epochs**. Left panel: For wake delta power, there was an overall interaction (drug x time of day within photoperiod, *F*(12, 182.27) = 2.47, *p* = 0.005) and a secondary interaction (drug x photoperiod, *F*(2, 185.16) = 3.62, *p* = 0.029) with a main effect of photoperiod (*F*(1, 176.67) = 34.22, *p* < 0.001). Specifically, there was increased wake delta power during the first half of the dark photoperiod on the recovery day (ZT 12–18: *t*(191.85) ≥ 2.94, *p* ≤ 0.007). Middle panel: For wake theta power, there was an overall interaction (drug x time of day within photoperiod, *F*(12, 180.96) = 2.05, *p* = 0.022), but there were no pair-wise differences at any time point on either the drug or recovery days and vehicle. Right panel: No effect of AM3506 treatment on wake gamma power. **E, NREM epochs**. Left panel: For NREM delta power, there was an overall interaction (drug x time of day within photoperiod, *F*(12, 178.50) = 3.88, *p* < 0.001) with a main effect of photoperiod (*F*(1, 115.69) = 6.88, *p* = 0.010). On the recovery day, NREM delta was elevated at one point during the dark photoperiod (ZT 15–18: *t*(151.37) = 2.55, *p* = 0.024). Middle panel: No effect of AM3506 on NREM theta. Right panel: For NREM gamma power, there was a secondary interaction (drug x photoperiod, *F*(2, 185.23) = 3.54, *p* = 0.031) and a nested interaction (time of day within photoperiod, *F*(6, 180.37) = 8.35, *p* < 0.001) with a main effect of drug treatment (*F*(2, 171.77) = 9.94, *p* < 0.001). Overall, AM3506 reduced NREM gamma (*t*(155.32) = -3.45, *p* = 0.001), and relative to vehicle, AM3506 specifically reduced NREM gamma across the first 18 Hr of the experiment (ZT 12–06: *t*(170.04) ≤ -2.42, *p* ≤ 0.033). **F, REM epochs**. Left panel: No effect of AM3506 on REM delta power. Middle panel: For REM theta, there was a significant overall interaction (drug x time of day within photoperiod, *F*(12, 157.56) = 1.96, *p* = 0.031) with a main effect of photoperiod (*F*(1, 104.31) = 6.78, *p* = 0.011). Compared to vehicle, REM theta was increased during the first 3 Hr immediately following administration of AM3506 (*t*(63.88) = 3.17, *p* = 0.005). Right panel: For REM gamma power, there was an overall interaction (drug x time of day within photoperiod, *F*(12, 166.38) = 2.09, *p* = 0.020), a secondary interaction (drug x photoperiod, *F*(2, 172.23) = 3.29, *p* = 0.039) with main effects of drug (*F*(2, 148.36) = 8.64, *p* < 0.001) and photoperiod (*F*(1, 146.71) = 19.90, *p* < 0.001). Overall, REM gamma power was reduced following AM3506 administration (*t*(125.06) = -2.67, *p* = 0.017) with specific reduction in REM gamma during the first 9 Hr of the experiment (ZT 12–21: *t*(143.63) ≤ -3.10, *p* ≤ 0.005). Symbols/Bars represent mean±SEM for 3 Hr time bins (*N* = 9). Grey background in graphs shows dark photoperiod. Asterisks denote significant difference from vehicle baseline. All injections administered at onset of dark photoperiod (ZT 12:00).
(PDF)

S8 Fig Power Spectral Features of EEG are Altered by Administration of the CB1 Antagonist AM281 Before the Dark Photoperiod. **A—C**, Power spectra from different vigilance states averaged over 12 Hr light/dark photoperiods. Dark lines represent group means and shaded regions surrounding the lines represent SEM. **A**, Wake. **B**, NREM. **C**, REM. **D–F**, Quantification of delta (0–4 Hz), theta (4–8 Hz), and gamma (30-60Hz) bandwidths of power spectra across the three vigilance states. **D, Wake epochs**. Left panel: For delta power, there was a significant overall interaction (drug x time of day within photoperiod, *F*(12, 232.82) = 2.05, *p* = 0.021) with a main effect of drug treatment (*F*(2, 61.15) = 12.80, *p* < 0.001). 5.0 mg/kg AM281 significantly increased delta power during wake epochs across most of the experiment (ZT 12–21 & 00–09; *t*(144.52) ≥ 2.71, *p* ≤ 0.015). Middle panel: For wake theta power, there was a nested interaction (time of day within photoperiod, *F*(6, 237.52) = 3.78, *p* = 0.001) with a main effect of drug treatment (*F*(2, 72.91) = 7.53, *p* < 0.001). Theta power was increased at one time point during the dark photoperiod (ZT 15–18: *t*(159.69) = 2.18, *p* = 0.049) and the first half of the light photoperiod (ZT 00–06: *t*(159.69) ≥ 2.87, *p* ≤ 0.009). Right panel: There was no effect of treatment on power in the gamma bandwidth. **E, NREM epochs**. Left panel: For NREM delta power, there was a significant overall interaction (drug x time of day within photoperiod, *F*(12, 243.32) = 2.77, *p* = 0.002), a secondary interaction (drug x photoperiod, *F*(2, 248.67) = 6.84, *p* = 0.001), a nested interaction (time of day within photoperiod, *F*(6, 232.06) = 30.63, *p* < 0.001), and main effects of both drug treatment (*F*(2, 104.70) = 35.00, *p* < 0.001) and photoperiod (*F*(1, 156.74) = 45.51, *p* < 0.001). Specifically, high dose AM281 increased NREM delta power across most time bins (ZT 12–21: *t*(195.82) ≥ 2.89, *p* ≤ 0.009). Middle panel: For NREM theta power, there was a significant overall interaction (drug x time of day within photoperiod, *F*(12, 247.79) = 2.40, *p* = 0.006), a nested interaction (time of day within photoperiod, *F*(6, 238.8) = 11.47, *p* < 0.001), and main effects of both photoperiod (*F*(1, 187.29) = 58.41, *p* < 0.001) and treatment (*F*(2, 189.81) = 19.12, *p* < 0.001). Pair-wise comparisons found that high dose AM281 increased NREM theta power across much of the day starting 3 Hr after drug administration (ZT 15–21: *t*(250.10) ≥ 2.32, *p* ≤ 0.042). Right panel: For NREM gamma, there was an overall interaction (*F*(12, 248.00) = 5.11, *p* < 0.001), a nested interaction (time of day within photoperiod, *F*(6,241.11) = 10.37, *p* < 0.001), and a main effect of photoperiod (*F*(1, 198.44) = 83.52, *p* < 0.001). Following AM281 administration, NREM gamma power was increased at only one time point during the dark photoperiod (ZT 06–09: *t*(255.10) = 2.60, *p* = 0.020). **F, REM epochs**. Left panel: For REM delta power, there was a secondary interaction (drug x photoperiod, *F*(2, 167.31) = 7.24, *p* = 0.001), a nested interaction (time of day within photoperiod, *F*(6, 204.83) = 4.16, *p* = 0.001), and main effects of drug treatment (*F*(2, 61.28) = 14.18, *p* < 0.001) and photoperiod (*F*(1, 195.42) = 38.71, *p* < 0.001). Specifically, AM281 increased REM delta across all time points in the dark photoperiod (ZT12-00: *t*(172.89) = 3.20, *p* ≤ 0.003). Middle panel: For REM theta power, there was an overall interaction (drug x time of day within photoperiod, *F*(12, 211.73) = 2.20, *p* = 0.013), a nested interaction (time of day within photoperiod, *F*(6, 208.14) = 2.52, *p* = 0.023), and a main effect of drug treatment (*F*(2, 79.84) = 9.51, *p* < 0.001). There were several time points over the day when AM281 administration increased REM theta power (ZT 15–03: *t*(164.93) ≥ 2.42, *p* ≤ 0.033). Right panel: There was no effect of AM281 treatment on REM gamma power. Symbols/Bars represent mean±SEM for 3 hr time bins (*N* = 12). Grey background in graphs shows dark photoperiod. Asterisks denote significant difference from vehicle baseline. All injections administered at onset of DP (ZT 12:00).
(PDF)

S9 Fig Blockade of CB1 Does Not Alter Recovery of Sleep Following 6 Hr TSD. To determine if sleep homeostatic mechanisms were altered by administration of the CB1 antagonist immediately following 6 Hr of acute TSD, we determined the sleep deficit incurred during TSD and computed the recovery from this deficit. **A,** First the cumulative NREM sleep time was calculated for each subject in both groups over sequential 3 Hr bins across three phases of the experiment: baseline vehicle administration, sleep deprivation, and the recovery day immediately following sleep deprivation. **B**, Second, each subject’s baseline cumulative sleep was subtracted from each of these three curves. The baseline day is only shown here to demonstrate this normalization. **C**, The sleep debt incurred by each animal after sleep deprivation was taken as the last bin of the baseline normalized cumulative sleep on the deprivation day. This value was separately calculated for each subject and was subtracted from each point of the baseline normalized cumulative sleep plot for the recovery day (open symbols panel **B**). This yielded the two curves depicted in panel **C** for the recovery from sleep debt incurred during TSD. A two-way repeated measures ANOVA was performed on the NREM recovery data shown in panel **C** with treatment group as a between-groups factor and time of day as a within-subjects repeated measure. There was a significant main effect of time of day (*F*(7, 126) = 51.62, *p* < 0.001), but there was neither an interaction (*F*(7, 126) = 1.35, *p* = 0.23) nor a main effect of treatment (*F*(1, 18) = 0.75, *p* = 0.40), suggesting that both groups recovered similarly from TSD. In panels **A** and **B**, the red arrows denote the two time bins when the sleep deprivation device was activated. There was generally lower overall sleep for most of sleep deprivation day in both groups, even prior to activation of the rotor. However, this is not surprising given that the subjects had just been placed into the deprivation chambers and were likely habituating to the new environment. Symbols/Bars represent mean±SEM for 3 Hr time bins. Grey background in graphs shows dark photoperiod. Injections were delivered on baseline and recovery days half-way through the LP (ZT 06:00). On the baseline day, both groups received a vehicle injection. On the recovery day, the vehicle group (*N* = 11) received another vehicle injection, while the AM281 group (*N* = 9) received a 5.0 mg/kg injection of AM281.
(PDF)

S10 Fig Treatment with AM281 Results in a Late Rebound in REM. **A**, Diagram of experimental protocol repeated here for clarity with different color coding to indicate measures reflect REM sleep parameters. **B**, Overall fluctuation in REM sleep throughout the entire experiment. Downward facing arrows denote times at which injections were given. The red horizontal line indicates the time at which the sleep deprivation chambers were activated. **C**, Comparisons within and between treatment groups across the first baseline and recovery days. Top Graph: Percent time in REM sleep. There was a significant overall interaction (treatment group x time of day within photoperiod within experimental phase, *F*(24, 270) = 7.01, *p* < 0.001), a secondary interaction (treatment group x photoperiod within experimental phase, *F*(3, 270) = 3.56, *p* = 0.015), a tertiary interaction (treatment group x experimental phase, *F*(1, 270) = 6.71, *p* = 0.010), and a main effect of photoperiod (*F*(1, 270) = 32.26, *p* < 0.001). For the vehicle group, there was significantly less REM sleep overall on the recovery day compared to baseline (*t*(270) = -2.64, *p* = 0.009), but there was not an overall difference between groups for the amount of REM on either the baseline or recovery days. However, the AM281 group had significantly more REM sleep than vehicle treated mice towards the end of the recovery day (ZT 21–06: *t*(98.63) ≥ 2.01, *p* ≤ 0.047). Compared to their baseline sleep, both vehicle and AM281 treated mice had significantly less REM during the first 3 Hr of the recording (ZT 06–09: *t*(270) ≤ -3.13, *p* ≤ 0.002), but the AM281 group went on to exhibit a REM rebound late in the recording (ZT 21–06: *t*(270) ≥ 2.12, *p* ≤ 0.035), while the vehicle group continued to have less REM than their baseline (ZT 00–03: *t*(270) = -2.51, *p* = 0.013). Middle Graph: REM bout duration. There was no effect of either sleep deprivation or AM281 treatment on REM bout duration in this experiment. This is possibly because the estimates of REM bout duration were taken from a small number of REM bouts for each subject following sleep deprivation. Bottom graph: For the number of REM bouts, there was an overall interaction (treatment group x time of day within photoperiod within experimental phase, *F*(24, 270) = 6.78, *p* < 0.001), a secondary interaction (treatment group x photoperiod within experimental phase, *F*(3, 270) = 9.94, *p* < 0.001), a tertiary interaction (treatment group x experimental phase, *F*(1, 270) = 4.20, *p* = 0.041), and a main effect of photoperiod (*F*(1, 270) = 11.22, *p* = 0.001). However, the AM281 group had fewer REM bouts than vehicle group during the DP on the baseline day (*t*(40.83) = -2.86, *p* = 0.007). Specifically, on the baseline day the AM281 group had fewer REM bouts than the vehicle group, during the first 9 Hr of the DP (ZT12-21: *t*(160.70) = -2.34, *p* = 0.020) and significantly more REM bouts during the first 3 Hr of the subsequent LP (ZT00-03, *t*(160.70) = 2.21, *p* = 0.029). Consequently, the accentuated baseline circadian fluctuation in REM in the AM281 group should be taken into account when interpreting between-group differences during recovery. Relative to their own baselines, both groups had a reduction in REM bouts during the first 3 Hr of the recovery (ZT06-09: *t*(270) ≤ -2.29, *p* = 0.023). For the vehicle group, this reduction in the number of REM bouts continued into the DP (ZT18-21: *t*(270) = -4.07, *p* < 0.001), while for the AM281 group, the number of REM bouts increased above baseline levels late in the recovery (ZT00-06: *t*(270) ≥ 3.14, *p* ≤ 0.002). Late in the recovery day, the AM281 group had significantly more REM bouts compared to vehicle treated mice at the same time points where the AM281 group had more REM bouts than its own baseline (ZT00-06: *t*(270) ≥ 4.03, *p* < 0.001). Additionally, considering there were baseline differences between groups at only one of these time points (ZT00-03), it is likely that this late augmentation in the number of REM bouts reflects a real phenomenon and is not a reflection of a pre-existing group difference. Nevertheless, obtaining a measure where baseline differences do not exist between groups would provide more confidence that another, uncontrolled factor is not involved in this process. Green symbols/lines show AM281 group (*N* = 9) while the vehicle group (*N* = 11) is depicted in black. Downward facing arrows (↓) indicate time of drug administration. Asterisks (*) denote significant pair-wise comparisons within-groups between drug conditions and measures obtained during vehicle baseline. Daggers (**†**) denote significant pair-wise comparisons between groups on the recovery day. Pound symbols (#) denote significant pair-wise comparisons between groups on the baseline day. The legend on the bottom right corresponds to panel **C**. In **B** & **C**, Grey shaded regions indicate the DP, and symbols/bars represent means±SEM across all subjects for each 3 Hr time bin
(PDF)

S11 Fig Blockade of CB1 During Recovery from TSD Significantly Augments Low Frequency Power Spectral Features, Confounding Attempts to Use These as Indices of Sleep Homeostatic Drive. **A,** Diagram of experimental protocol for sleep deprivation. **B,** Wake theta power during sleep deprivation. Horizontal red bar indicates sleep deprivation session during first 6 Hr of the LP. **C, Wake epochs.** *Left panel*: For wake delta, there was an overall interaction (treatment group x time of day within photoperiod within experimental phase, *F*(12, 259.47) = 12.27, *p* < 0.001) and a secondary interaction (treatment group x experimental phase, *F*(1, 192.08) = 16.66, *p* < 0.001). For the first 6 Hr following TSD, wake delta was increased by AM281 administration relative to delta power measurements in the vehicle group (ZT06-12: *t*(35.02) ≥ 3.20, *p* ≤ 0.003). Additionally, within-group comparisons found that AM281 elevated wake delta across the first 12 Hr of recovery from TSD (ZT06-18: *t*(255.42) ≥ 2.09, *p* ≤ 0.038). In contrast, wake delta power was reduced in the vehicle treated group but only during the last 3 Hr of the recovery (ZT03-06: *t*(255.42) = -2.04, *p* = 0.042). *Middle panel*: For wake theta, there was an overall interaction (treatment group x time of day within photoperiod within experimental phase, *F*(12, 259.47) = 12.27, *p* < 0.001) and a secondary interaction (treatment group x experimental phase, *F*(1, 185.67) = 6.26, *p* = 0.013). There were no pair-wise differences between groups during baseline or recovery. However, treatment with AM281 increased wake theta relative to baseline during the first 12 Hr of recovery (ZT06-18: *t*(252.32) ≥ 2.18, *p* ≤ 0.030). For the vehicle group, there were no pair-wise differences in wake theta between baseline and recovery. *Right panel*: For wake gamma, there was a nested interaction (time of day within photoperiod within experimental phase, *F*(12, 255.50) = 13.77, *p* < 0.001) and a main effect of experimental phase (*F*(1, 139.37) = 9.85, *p* = 0.002). Across treatment groups, there was an overall reduction of wake gamma power during recovery from TSD relative to baseline (*t*(139.37) = -3.14, *p* = 0.002). **D, NREM epochs.** *Left panel*: For NREM delta, there was an overall interaction (treatment group x time of day within photoperiod within experimental phase, *F*(12, 255.42) = 8.84, *p* < 0.001), secondary interaction (treatment x experimental phase, *F*(1, 156.93) = 25.84, *p* < 0.001), and a main effect of experimental phase (*F*(1, 156.93) = 6.37, *p* = 0.013). Relative to the vehicle group, AM281 administration during recovery from TSD increased NREM delta power across the first 15 Hr of the recording (ZT06-21: *t*(38.44) ≥ 2.60, *p* ≤ 0.013). Within-groups comparisons between recovery and baseline found increased NREM delta power following AM281 administration after TSD across the majority of the recording (ZT06-00: *t*(233.13) ≥ 2.18, *p* ≤ 0.030). For the vehicle group, NREM delta during recovery was reduced relative to baseline measures during the first 9 Hr of the DP (ZT12-21: *t*(233.13) ≤ -2.27, *p* ≤ 0.024). *Middle panel*: For NREM theta power, there was an overall interaction (treatment group x time of day within photoperiod within experimental phase, *F*(12, 263.39) = 5.07, *p* < 0.001) and a secondary interaction (treatment group x experimental phase, *F*(1, 238.82) = 18.25, *p* < 0.001). Between groups comparisons on the recovery day found increased theta power in the AM281-treated group (ZT06-21: *t*(29.42) ≥ 3.04, *p* ≤ 0.005), but there were no differences between groups during baseline. Within groups comparisons found elevated NREM theta in the AM281 group during recovery relative to baseline (ZT06-21: *t*(277.23) ≥ 2.84, *p* ≤ 0.005). In contrast, NREM theta was reduced at several time points during recovery in the vehicle-treated group (ZT09-21: *t*(277.23) ≤ -2.01, *p* ≤ 0.045). *Right panel*: For NREM gamma power, there was an overall interaction (treatment group x time of day within photoperiod within experimental phase, *F*(12, 264.25) = 2.73, *p* < 0.002). There were no pair-wise differences between groups during baseline or recovery. However, for the AM281 group, NREM gamma was increased relative to baseline for the first 6 Hr of the DP (ZT12-18: *t*(281.46) ≥ 2.44, *p* ≤ 0.015). **E, REM Epochs.** *Left panel*: For REM delta power, there was a secondary interaction between treatment group and experimental phase (*F*(1, 85.32) = 6.01, *p* = 0.016). There were no differences between groups during either baseline or recovery phases of the experiments. However, for the AM281 group, there was an overall increase in REM delta during recovery relative to baseline (*t*(86.08) = 2.80, *p* = 0.006). There was no difference between baseline and recovery for the vehicle treated group. *Middle panel*: For REM theta power, there was an overall interaction (treatment group x time of day within photoperiod within experimental phase, *F*(12, 216.66) = 1.83, *p* = 0.045) with main effects of treatment group (*F*(1, 28.36) = 18.34, *p* < 0.001) and experimental phase (*F*(1, 59.83) = 4.26, *p* = 0.043). There were no differences between treatment groups during baseline, but REM theta was elevated at all time points of the recovery day relative to the vehicle group (ZT 06–06: *t*(106.40) ≥ 1.99, *p* ≤ 0.049). Within-groups comparisons between recovery and baseline found increased REM theta during the first 9 Hr of recovery in the AM281 group (ZT 06–15: *t*(170.25) ≥ 2.31, *p* ≤ 0.022). There were no differences in REM theta between recovery and baseline recordings for the vehicle treated group. *Right panel*: There was no effect of TSD or AM281 treatment on REM gamma power. For B-D: Grey shaded regions denote dark photoperiod. Open symbols with dotted lines indicate data from the recovery day 1 while closed symbols with solid lines represent data from the baseline day 1. Asterisks (*) denote significant pair-wise comparisons within-groups between drug conditions and measures obtained during vehicle baseline. Daggers (**†**) denote significant pair-wise comparisons between groups on the recovery day. Symbols/bars represent means±SEM across all subjects for each 3 Hr time bin. For AM281 group *N* = 9, and for the vehicle group *N* = 11.
(PDF)

## References

[1] Russo E. Cannabis in India: ancient lore and modern medicine. In: Mechoulam R, editor. Cannabinoids as Therapeutics. Milestones in drug therapy. Basel; Boston: Birkhäuser; 2005. p. 1–22.

[2] O'Shaughnessy WB. On the Preparations of the Indian Hemp, or Gunjah: Cannabis Indica Their Effects on the Animal System in Health, and their Utility in the Treatment of Tetanus and other Convulsive Diseases. Prov Med J Retrosp Med Sci. 1843;5(123):363–9. PubMed Central PMCID: PMCPMC2490264. PMC5592602 30161735

[3] Wallich GC. Cannabis Indica. Br Med J. 1883;1(1173):1224 . 20750654 10.1136/bmj.1.1173.1224 PMC2372636

[4] Bradbury JB. The Croonian Lectures on some Points Connected with Sleep, Sleeplessness, and Hypnotics: Delivered before the Royal College of Physicians of London. Br Med J. 1899;2(2011):134–8. . 20758585 10.1136/bmj.2.2011.134 PMC2411681

[5] Clendinning J. Observations on the medicinal properties of the Cannabis Sativa of India. Medico-chirurgical transactions. 1843;26:188–210. PMC2116906. 20895771 10.1177/095952874302600116 PMC2116906

[6] Moreton JE, Davis WM. Electroencephalographic study of the effects of tetrahydrocannabinols on sleep in the rat. Neuropharmacology. 1973;12(9):897–907. Epub 1973/09/01. . 4355678 10.1016/0028-3908(73)90042-7

[7] Watanabe K, Narimatsu S, Yamamoto I, Yoshimura H. Difference in tolerance development of hypothermia and pentobarbital-induced sleep prolongating effect of 11-hydroxy-delta 8-tetrahydrocannabinol and 11-oxo-delta 8-tetrahydrocannabinol in mice. European journal of pharmacology. 1982;77(1):53–6. Epub 1982/01/08. . 6277654 10.1016/0014-2999(82)90535-0

[8] Watanabe K, Narimatsu S, Yamamoto I, Yoshimura H. Cross-tolerance development to the prolongation of pentobarbitone-induced sleep by delta 8-tetrahydrocannabinol and 11-hydroxy-delta 8-tetrahydrocannabinol in mice. The Journal of pharmacy and pharmacology. 1987;39(11):945–7. Epub 1987/11/01. . 2892923 10.1111/j.2042-7158.1987.tb03136.x

[9] Zarcone VP Jr. Marijuana and ethanol: effects on sleep. Int J Psychiatry Med. 1973;4(2):201–12. . 4352042 10.2190/ba5b-1lvx-0lq5-xv02

[10] Fujimori M, Himwich HE. Delta 9-tetrahydrocannabinol and the sleep-wakefulness cycle in rabbits. Physiology & behavior. 1973;11(3):291–5. Epub 1973/09/01. . 4355439 10.1016/0031-9384(73)90003-6

[11] Wallach MB, Gershon S. The effects of delta8-THC on the EEG, reticular multiple unit activity and sleep of cats. European journal of pharmacology. 1973;24(2):172–8. . 4358207 10.1016/0014-2999(73)90068-x

[12] Kano M, Ohno-Shosaku T, Hashimotodani Y, Uchigashima M, Watanabe M. Endocannabinoid-mediated control of synaptic transmission. Physiol Rev. 2009;89(1):309–80. 10.1152/physrev.00019.2008 . 19126760

[13] Murillo-Rodriguez E, Cabeza R, Mendez-Diaz M, Navarro L, Prospero-Garcia O. Anandamide-induced sleep is blocked by SR141716A, a CB1 receptor antagonist and by U73122, a phospholipase C inhibitor. Neuroreport. 2001;12(10):2131–6. Epub 2001/07/12. . 11447321 10.1097/00001756-200107200-00018

[14] Murillo-Rodriguez E, Sanchez-Alavez M, Navarro L, Martinez-Gonzalez D, Drucker-Colin R, Prospero-Garcia O. Anandamide modulates sleep and memory in rats. Brain research. 1998;812(1–2):270–4. Epub 1998/11/14. . 9813364 10.1016/s0006-8993(98)00969-x

[15] Murillo-Rodriguez E, Blanco-Centurion C, Sanchez C, Piomelli D, Shiromani PJ. Anandamide enhances extracellular levels of adenosine and induces sleep: an in vivo microdialysis study. Sleep. 2003;26(8):943–7. Epub 2004/01/30. . 14746372 10.1093/sleep/26.8.943

[16] Rueda-Orozco PE, Soria-Gomez E, Montes-Rodriguez CJ, Perez-Morales M, Prospero-Garcia O. Intrahippocampal administration of anandamide increases REM sleep. Neuroscience letters. 2010;473(2):158–62. Epub 2010/03/02. 10.1016/j.neulet.2010.02.044 . 20188142

[17] Murillo-Rodriguez E, Palomero-Rivero M, Millan-Aldaco D, Arias-Carrion O, Drucker-Colin R. Administration of URB597, oleoylethanolamide or palmitoylethanolamide increases waking and dopamine in rats. PloS one. 2011;6(7):e20766 10.1371/journal.pone.0020766 21779318 PMC3136458

[18] Murillo-Rodriguez E, Vazquez E, Millan-Aldaco D, Palomero-Rivero M, Drucker-Colin R. Effects of the fatty acid amide hydrolase inhibitor URB597 on the sleep-wake cycle, c-Fos expression and dopamine levels of the rat. European journal of pharmacology. 2007;562(1–2):82–91. Epub 2007/03/06. 10.1016/j.ejphar.2007.01.076 . 17336288

[19] Murillo-Rodriguez E, Millan-Aldaco D, Di Marzo V, Drucker-Colin R. The anandamide membrane transporter inhibitor, VDM-11, modulates sleep and c-Fos expression in the rat brain. Neuroscience. 2008;157(1):1–11. Epub 2008/09/30. 10.1016/j.neuroscience.2008.08.056 . 18822353

[20] Murillo-Rodriguez E, Palomero-Rivero M, Millan-Aldaco D, Di Marzo V. The administration of endocannabinoid uptake inhibitors OMDM-2 or VDM-11 promotes sleep and decreases extracellular levels of dopamine in rats. Physiology & behavior. 2013;109:88–95. Epub 2012/12/15. 10.1016/j.physbeh.2012.11.007 . 23238438

[21] Huitron-Resendiz S, Sanchez-Alavez M, Wills DN, Cravatt BF, Henriksen SJ. Characterization of the sleep-wake patterns in mice lacking fatty acid amide hydrolase. Sleep. 2004;27(5):857–65. . 15453543 10.1093/sleep/27.5.857

[22] Nathan PJ, O'Neill BV, Napolitano A, Bullmore ET. Neuropsychiatric adverse effects of centrally acting antiobesity drugs. CNS Neurosci Ther. 2011;17(5):490–505. 10.1111/j.1755-5949.2010.00172.x . 21951371 PMC6493804

[23] Steinberg BA, Cannon CP. Cannabinoid-1 receptor blockade in cardiometabolic risk reduction: safety, tolerability, and therapeutic potential. Am J Cardiol. 2007;100(12A):27P–32P. 10.1016/j.amjcard.2007.10.011 . 18154743

[24] Pava MJ, den Hartog CR, Blanco-Centurion C, Shiromani PJ, Woodward JJ. Endocannabinoid modulation of cortical up-states and NREM sleep. PloS one. 2014;9(2):e88672 Epub 2014/02/13. 10.1371/journal.pone.0088672 24520411 PMC3919802

[25] Silvani A, Berteotti C, Bastianini S, Lo Martire V, Mazza R, Pagotto U, et al Multiple sleep alterations in mice lacking cannabinoid type 1 receptors. PloS one. 2014;9(2):e89432 Epub 2014/03/04. 10.1371/journal.pone.0089432 24586776 PMC3930731

[26] Zimmer A, Zimmer AM, Hohmann AG, Herkenham M, Bonner TI. Increased mortality, hypoactivity, and hypoalgesia in cannabinoid CB1 receptor knockout mice. Proceedings of the National Academy of Sciences of the United States of America. 1999;96(10):5780–5. 10318961 10.1073/pnas.96.10.5780 PMC21937

[27] Wu CS, Zhu J, Wager-Miller J, Wang S, O'Leary D, Monory K, et al Requirement of cannabinoid CB(1) receptors in cortical pyramidal neurons for appropriate development of corticothalamic and thalamocortical projections. The European journal of neuroscience. 2010;32(5):693–706. 10.1111/j.1460-9568.2010.07337.x 21050275 PMC2970673

[28] Santucci V, Storme JJ, Soubrie P, Le Fur G. Arousal-enhancing properties of the CB1 cannabinoid receptor antagonist SR 141716A in rats as assessed by electroencephalographic spectral and sleep-waking cycle analysis. Life sciences. 1996;58(6):PL103–10. Epub 1996/01/01. . 8569415 10.1016/0024-3205(95)02319-4

[29] Goonawardena AV, Plano A, Robinson L, Ross R, Greig I, Pertwee RG, et al Modulation of food consumption and sleep-wake cycle in mice by the neutral CB1 antagonist ABD459. Behav Pharmacol. 2015;26(3):289–303. 10.1097/FBP.0000000000000108 25356730 PMC4445652

[30] Perez-Morales M, Alvarado-Capuleno I, Lopez-Colome AM, Mendez-Diaz M, Ruiz-Contreras AE, Prospero-Garcia O. Activation of PAR1 in the lateral hypothalamus of rats enhances food intake and REMS through CB1R. Neuroreport. 2012;23(14):814–8. Epub 2012/08/15. 10.1097/WNR.0b013e328357615a . 22889888

[31] Mendelson WB, Basile AS. The hypnotic actions of oleamide are blocked by a cannabinoid receptor antagonist. Neuroreport. 1999;10(15):3237–9. . 10574567 10.1097/00001756-199910190-00021

[32] Goonawardena AV, Plano A, Robinson L, Platt B, Hampson RE, Riedel G. A Pilot Study into the Effects of the CB1 Cannabinoid Receptor Agonist WIN55,212–2 or the Antagonist/Inverse Agonist AM251 on Sleep in Rats. Sleep disorders. 2011;2011:178469 Epub 2011/01/01. 10.1155/2011/178469 23471192 PMC3581240

[33] Liedhegner ES, Sasman A, Hillard CJ. Brain region-specific changes in N-acylethanolamine contents with time of day. J Neurochem. 2014;128(4):491–506. 10.1111/jnc.12495 24138639 PMC3946166

[34] Valenti M, Vigano D, Casico MG, Rubino T, Steardo L, Parolaro D, et al Differential diurnal variations of anandamide and 2-arachidonoyl-glycerol levels in rat brain. Cell Mol Life Sci. 2004;61(7–8):945–50. 10.1007/s00018-003-3453-5 . 15095014 PMC11138870

[35] National Research Council Committee for the Update of the Guide for the Care and Use of Laboratory Animals, Institute for Laboratory Animal Research, National Academies Press. Guide for the care and use of laboratory animals 8th ed. Washington, D.C.: National Academies Press; 2011 xxv, 220 p. p.

[36] Gervasoni D, Lin SC, Ribeiro S, Soares ES, Pantoja J, Nicolelis MA. Global forebrain dynamics predict rat behavioral states and their transitions. The Journal of neuroscience: the official journal of the Society for Neuroscience. 2004;24(49):11137–47. Epub 2004/12/14. 10.1523/JNEUROSCI.3524-04.2004 . 15590930 PMC6730270

[37] Mang GM, Franken P. Sleep and EEG Phenotyping in Mice. Curr Protoc Mouse Biol. 2012;2(1):55–74. 10.1002/9780470942390.mo110126 . 26069005

[38] Diniz Behn CG, Klerman EB, Mochizuki T, Lin SC, Scammell TE. Abnormal sleep/wake dynamics in orexin knockout mice. Sleep. 2010;33(3):297–306. 20337187 10.1093/sleep/33.3.297 PMC2831423

[39] Bastianini S, Berteotti C, Gabrielli A, Del Vecchio F, Amici R, Alexandre C, et al SCOPRISM: a new algorithm for automatic sleep scoring in mice. J Neurosci Methods. 2014;235:277–84. 10.1016/j.jneumeth.2014.07.018 . 25092499

[40] Weber F, Chung S, Beier KT, Xu M, Luo L, Dan Y. Control of REM sleep by ventral medulla GABAergic neurons. Nature. 2015;526(7573):435–8. 10.1038/nature14979 . 26444238 PMC4852286

[41] Srinivasan BV, Qi H, Duraiswami R. GPUML: Graphical processors for speeding up kernel machines. Siam Conference on Data Mining; April 2010; Columbus, OH2010.

[42] Vaughn LK, Denning G, Stuhr KL, de Wit H, Hill MN, Hillard CJ. Endocannabinoid signalling: has it got rhythm? British journal of pharmacology. 2010;160(3):530–43. Epub 2010/07/02. 10.1111/j.1476-5381.2010.00790.x 20590563 PMC2931554

[43] Vyazovskiy VV, Riedner BA, Cirelli C, Tononi G. Sleep homeostasis and cortical synchronization: II. A local field potential study of sleep slow waves in the rat. Sleep. 2007;30(12):1631–42. 18246973 10.1093/sleep/30.12.1631 PMC2276140

[44] Borbely AA, Achermann P. Sleep homeostasis and models of sleep regulation. J Biol Rhythms. 1999;14(6):557–68. . 10643753 10.1177/074873099129000894

[45] Kim T, Ramesh V, Dworak M, Choi DS, McCarley RW, Kalinchuk AV, et al Disrupted sleep-wake regulation in type 1 equilibrative nucleoside transporter knockout mice. Neuroscience. 2015;303:211–9. 10.1016/j.neuroscience.2015.06.037 26143012 PMC4532636

[46] Jacobs J. Hippocampal theta oscillations are slower in humans than in rodents: implications for models of spatial navigation and memory. Philos Trans R Soc Lond B Biol Sci. 2014;369(1635):20130304 10.1098/rstb.2013.0304 24366145 PMC3866455

[47] Fries P. Neuronal gamma-band synchronization as a fundamental process in cortical computation. Annu Rev Neurosci. 2009;32:209–24. 10.1146/annurev.neuro.051508.135603 . 19400723

[48] Fegley D, Gaetani S, Duranti A, Tontini A, Mor M, Tarzia G, et al Characterization of the fatty acid amide hydrolase inhibitor cyclohexyl carbamic acid 3'-carbamoyl-biphenyl-3-yl ester (URB597): effects on anandamide and oleoylethanolamide deactivation. J Pharmacol Exp Ther. 2005;313(1):352–8. 10.1124/jpet.104.078980 . 15579492

[49] Gunduz-Cinar O, MacPherson KP, Cinar R, Gamble-George J, Sugden K, Williams B, et al Convergent translational evidence of a role for anandamide in amygdala-mediated fear extinction, threat processing and stress-reactivity. Mol Psychiatry. 2013;18(7):813–23. 10.1038/mp.2012.72 22688188 PMC3549323

[50] Dijk DJ, Brunner DP, Beersma DG, Borbely AA. Electroencephalogram power density and slow wave sleep as a function of prior waking and circadian phase. Sleep. 1990;13(5):430–40. . 2287855 10.1093/sleep/13.5.430

[51] Borbely AA. A two process model of sleep regulation. Hum Neurobiol. 1982;1(3):195–204. . 7185792

[52] Dzirasa K, Ribeiro S, Costa R, Santos LM, Lin SC, Grosmark A, et al Dopaminergic control of sleep-wake states. The Journal of neuroscience: the official journal of the Society for Neuroscience. 2006;26(41):10577–89. 10.1523/JNEUROSCI.1767-06.2006 . 17035544 PMC6674686

[53] Lin SC, Gervasoni D. Defining Global Brain States Using Multielectrode Field Potential Recordings. In: Nicolelis MAL, editor. Methods for Neural Ensemble Recordings. Frontiers in Neuroscience. 2nd ed. Boca Raton (FL)2008. 21204450

[54] Sunagawa GA, Sei H, Shimba S, Urade Y, Ueda HR. FASTER: an unsupervised fully automated sleep staging method for mice. Genes to cells: devoted to molecular & cellular mechanisms. 2013;18(6):502–18. 10.1111/gtc.12053 23621645 PMC3712478

[55] Savinainen JR, Jarvinen T, Laine K, Laitinen JT. Despite substantial degradation, 2-arachidonoylglycerol is a potent full efficacy agonist mediating CB(1) receptor-dependent G-protein activation in rat cerebellar membranes. British journal of pharmacology. 2001;134(3):664–72. 10.1038/sj.bjp.0704297 11588122 PMC1572991

[56] Melvin LS, Milne GM, Johnson MR, Subramaniam B, Wilken GH, Howlett AC. Structure-activity relationships for cannabinoid receptor-binding and analgesic activity: studies of bicyclic cannabinoid analogs. Mol Pharmacol. 1993;44(5):1008–15. . 8246904

[57] Compton DR, Johnson MR, Melvin LS, Martin BR. Pharmacological profile of a series of bicyclic cannabinoid analogs: classification as cannabimimetic agents. J Pharmacol Exp Ther. 1992;260(1):201–9. . 1309872

[58] Mackie K, Devane WA, Hille B. Anandamide, an endogenous cannabinoid, inhibits calcium currents as a partial agonist in N18 neuroblastoma cells. Mol Pharmacol. 1993;44(3):498–503. . 8371711

[59] Garzon J, de la Torre-Madrid E, Rodriguez-Munoz M, Vicente-Sanchez A, Sanchez-Blazquez P. Gz mediates the long-lasting desensitization of brain CB1 receptors and is essential for cross-tolerance with morphine. Mol Pain. 2009;5:11 10.1186/1744-8069-5-11 19284549 PMC2657119

[60] Zhuang S, Kittler J, Grigorenko EV, Kirby MT, Sim LJ, Hampson RE, et al Effects of long-term exposure to delta9-THC on expression of cannabinoid receptor (CB1) mRNA in different rat brain regions. Brain Res Mol Brain Res. 1998;62(2):141–9. . 9813289 10.1016/s0169-328x(98)00232-0

[61] Martinez-Vargas M, Morales-Gomez J, Gonzalez-Rivera R, Hernandez-Enriquez C, Perez-Arredondo A, Estrada-Rojo F, et al Does the neuroprotective role of anandamide display diurnal variations? Int J Mol Sci. 2013;14(12):23341–55. 10.3390/ijms141223341 24287910 PMC3876049

[62] Yasuo S, Koch M, Schmidt H, Ziebell S, Bojunga J, Geisslinger G, et al An endocannabinoid system is localized to the hypophysial pars tuberalis of Syrian hamsters and responds to photoperiodic changes. Cell Tissue Res. 2010;340(1):127–36. 10.1007/s00441-010-0930-7 . 20165884

[63] Acuna-Goycolea C, Obrietan K, van den Pol AN. Cannabinoids excite circadian clock neurons. The Journal of neuroscience: the official journal of the Society for Neuroscience. 2010;30(30):10061–6. 10.1523/JNEUROSCI.5838-09.2010 20668190 PMC2927117

[64] Sanford AE, Castillo E, Gannon RL. Cannabinoids and hamster circadian activity rhythms. Brain research. 2008;1222:141–8. 10.1016/j.brainres.2008.05.048 . 18582849

[65] Davis CJ, Clinton JM, Jewett KA, Zielinski MR, Krueger JM. Delta wave power: an independent sleep phenotype or epiphenomenon? J Clin Sleep Med. 2011;7(5 Suppl):S16–8. 10.5664/JCSM.1346 22003323 PMC3190419

[66] Baud MO, Magistretti PJ, Petit JM. Sustained sleep fragmentation induces sleep homeostasis in mice. Sleep. 2015;38(4):567–79. 10.5665/sleep.4572 25325477 PMC4355896

[67] He J, Kastin AJ, Wang Y, Pan W. Sleep fragmentation has differential effects on obese and lean mice. J Mol Neurosci. 2015;55(3):644–52. 10.1007/s12031-014-0403-7 25152064 PMC4320048

[68] Ringgold KM, Barf RP, George A, Sutton BC, Opp MR. Prolonged sleep fragmentation of mice exacerbates febrile responses to lipopolysaccharide. J Neurosci Methods. 2013;219(1):104–12. 10.1016/j.jneumeth.2013.07.008 23872243 PMC3993011

[69] Hajos M, Hoffmann WE, Kocsis B. Activation of cannabinoid-1 receptors disrupts sensory gating and neuronal oscillation: relevance to schizophrenia. Biol Psychiatry. 2008;63(11):1075–83. 10.1016/j.biopsych.2007.12.005 . 18261715

[70] Hajos N, Katona I, Naiem SS, MacKie K, Ledent C, Mody I, et al Cannabinoids inhibit hippocampal GABAergic transmission and network oscillations. The European journal of neuroscience. 2000;12(9):3239–49. . 10998107 10.1046/j.1460-9568.2000.00217.x

[71] Kucewicz MT, Tricklebank MD, Bogacz R, Jones MW. Dysfunctional prefrontal cortical network activity and interactions following cannabinoid receptor activation. The Journal of neuroscience: the official journal of the Society for Neuroscience. 2011;31(43):15560–8. 10.1523/JNEUROSCI.2970-11.2011 . 22031901 PMC6703515

[72] Cortes-Briones J, Skosnik PD, Mathalon D, Cahill J, Pittman B, Williams A, et al Delta(9)-THC Disrupts Gamma (gamma)-Band Neural Oscillations in Humans. Neuropsychopharmacology: official publication of the American College of Neuropsychopharmacology. 2015;40(9):2124–34. 10.1038/npp.2015.53 . 25709097 PMC4613601

[73] Robbe D, Montgomery SM, Thome A, Rueda-Orozco PE, McNaughton BL, Buzsaki G. Cannabinoids reveal importance of spike timing coordination in hippocampal function. Nat Neurosci. 2006;9(12):1526–33. 10.1038/nn1801 . 17115043

[74] Buonamici M, Young GA, Khazan N. Effects of acute delta 9-THC administration on EEG and EEG power spectra in the rat. Neuropharmacology. 1982;21(8):825–9. . 6289162 10.1016/0028-3908(82)90071-5

[75] Sales-Carbonell C, Rueda-Orozco PE, Soria-Gomez E, Buzsaki G, Marsicano G, Robbe D. Striatal GABAergic and cortical glutamatergic neurons mediate contrasting effects of cannabinoids on cortical network synchrony. Proceedings of the National Academy of Sciences of the United States of America. 2013;110(2):719–24. Epub 2012/12/28. 10.1073/pnas.1217144110 23269835 PMC3545808

[76] Turkanis SA, Karler R. Central excitatory properties of delta 9-tetrahydrocannabinol and its metabolites in iron-induced epileptic rats. Neuropharmacology. 1982;21(1):7–13. . 6278353 10.1016/0028-3908(82)90204-0

[77] Kim D, Hwang E, Lee M, Sung H, Choi JH. Characterization of topographically specific sleep spindles in mice. Sleep. 2015;38(1):85–96. 10.5665/sleep.4330 25325451 PMC4262960

[78] Ryan LJ. Characterization of cortical spindles in DBA/2 and C57BL/6 inbred mice. Brain Res Bull. 1984;13(4):549–58. . 6441615 10.1016/0361-9230(84)90037-6

[79] Perez-Morales M, De La Herran-Arita AK, Mendez-Diaz M, Ruiz-Contreras AE, Drucker-Colin R, Prospero-Garcia O. 2-AG into the lateral hypothalamus increases REM sleep and cFos expression in melanin concentrating hormone neurons in rats. Pharmacology, biochemistry, and behavior. 2013;108:1–7. Epub 2013/04/23. 10.1016/j.pbb.2013.04.006 . 23603032

[80] Jacobson LH, Commerford SR, Gerber SP, Chen YA, Dardik B, Chaperon F, et al Characterization of a novel, brain-penetrating CB1 receptor inverse agonist: metabolic profile in diet-induced obese models and aspects of central activity. Naunyn-Schmiedeberg's archives of pharmacology. 2011;384(6):565–81. Epub 2011/09/29. 10.1007/s00210-011-0686-y . 21947251

[81] Feinberg I, Jones R, Walker J, Cavness C, Floyd T. Effects of marijuana extract and tetrahydrocannabinol on electroencephalographic sleep patterns. Clin Pharmacol Ther. 1976;19(6):782–94. . 178475 10.1002/cpt1976196782

[82] Ceccarini J, Kuepper R, Kemels D, van Os J, Henquet C, Van Laere K. [18F]MK-9470 PET measurement of cannabinoid CB1 receptor availability in chronic cannabis users. Addict Biol. 2015;20(2):357–67. 10.1111/adb.12116 . 24373053

[83] Sim LJ, Hampson RE, Deadwyler SA, Childers SR. Effects of chronic treatment with delta9-tetrahydrocannabinol on cannabinoid-stimulated [35S]GTPgammaS autoradiography in rat brain. The Journal of neuroscience: the official journal of the Society for Neuroscience. 1996;16(24):8057–66. . 8987831 10.1523/JNEUROSCI.16-24-08057.1996 PMC6579228

[84] American Psychiatric Association. Diagnostic and statistical manual of mental disorders: DSM-5 5th ed. Washington, D.C.: American Psychiatric Association; 2013 xliv, 947 p. p.

[85] Gates P, Albertella L, Copeland J. Cannabis withdrawal and sleep: A systematic review of human studies. Subst Abus. 2015:0 10.1080/08897077.2015.1023484 . 25893849

[86] Fernandez-Mendoza J, Shea S, Vgontzas AN, Calhoun SL, Liao D, Bixler EO. Insomnia and incident depression: role of objective sleep duration and natural history. J Sleep Res. 2015;24(4):390–8. 10.1111/jsr.12285 . 25728794 PMC7155163

