# Supplementary figures and images for: Endocannabinoid Signaling Regulates Sleep Stability
Source: PLoS One. 2016 Mar 31;11(3):e0152473. doi: 10.1371/journal.pone.0152473 (PMC4816426; doi:10.1371/journal.pone.0152473)

**A**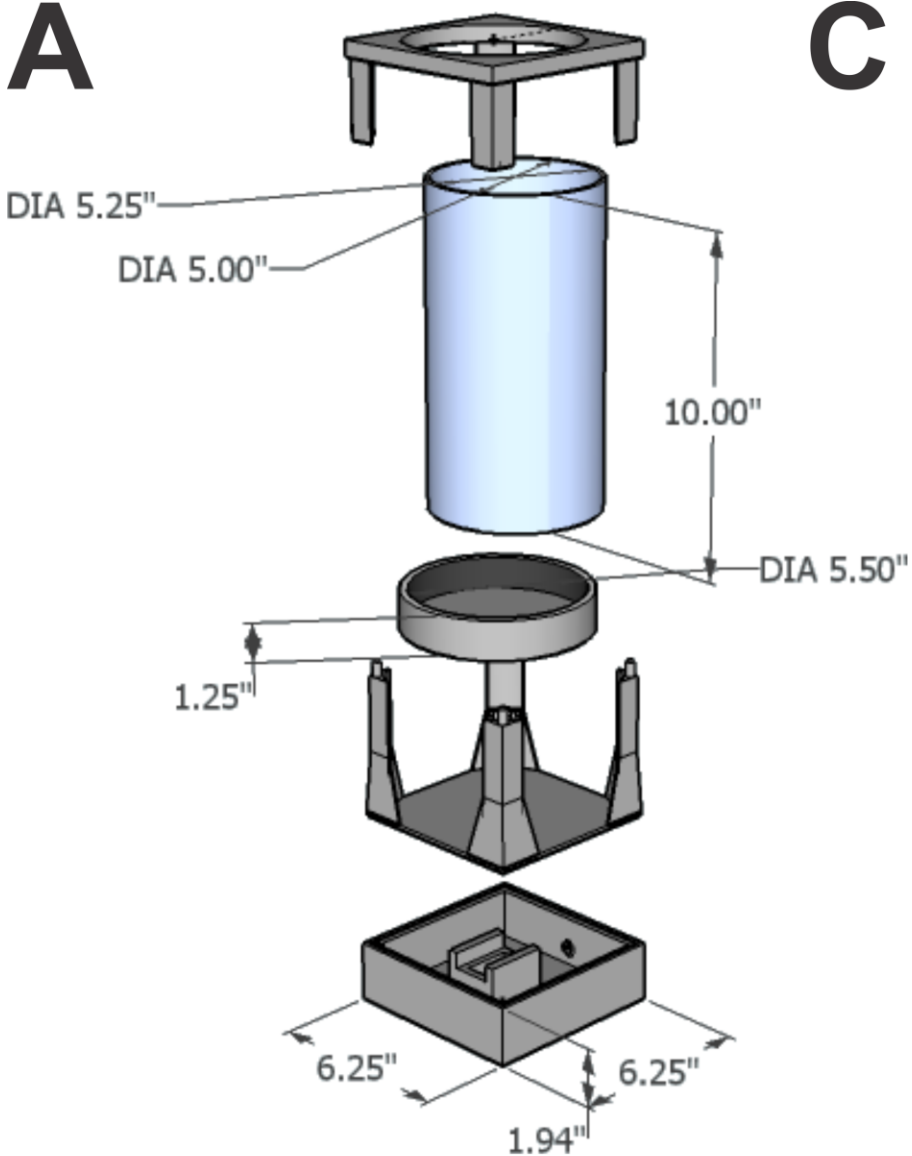**B**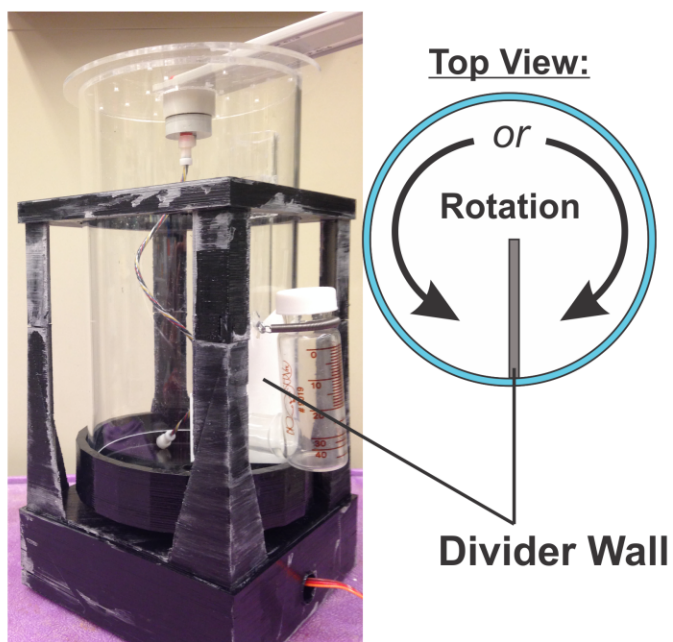**C**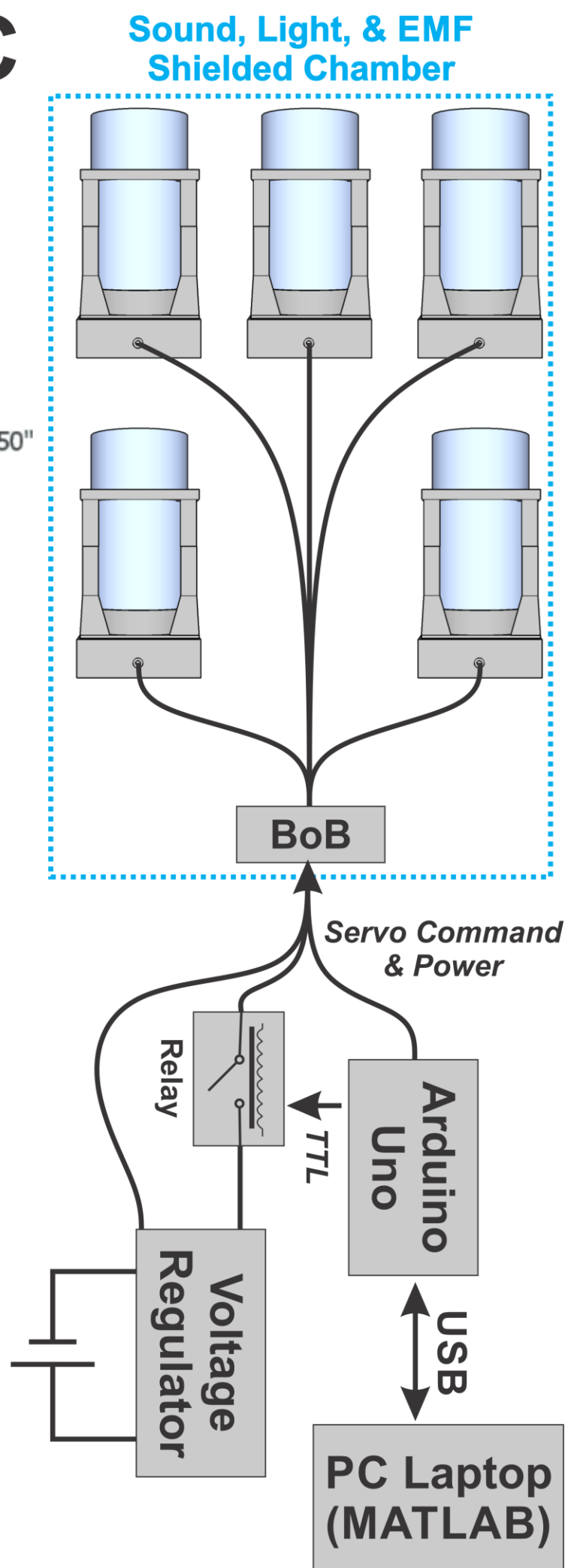

Supplement: S1 Fig — A, Exploded schematic view of structural components of the sleep deprivation chambers labelled with dimensions in inches. B, Photograph of an assembled device and a schematic of a top down view of the chamber. Note that the rotation described in the schematic implies rotation of the chamber floor/disc suspended beneath the clear acrylic chamber wall. Also note the commutator and tether in the photograph. Polysomnographic activity can be recorded in these chambers during the sleep deprivation. C, Schematic overview of assembled system including electronic control components: break-our board (BoB), Arduino, TTL controlled relay to control power circuit to motors, voltage regulator, and computer running custom control software written in MATLAB. (PDF) [file pone.0152473.s002.pdf]

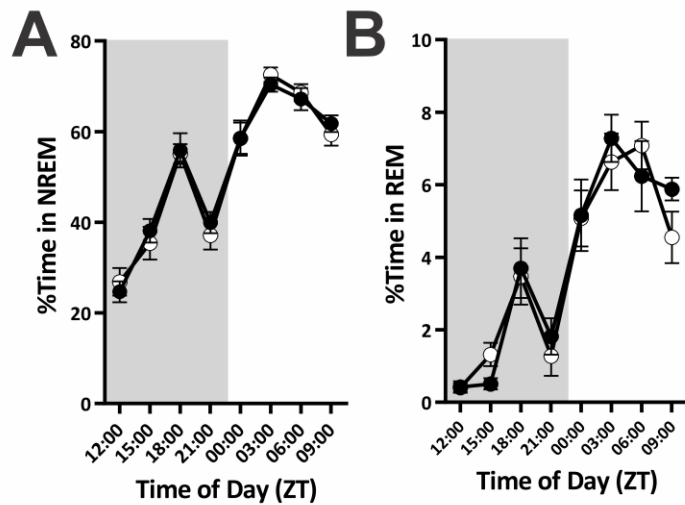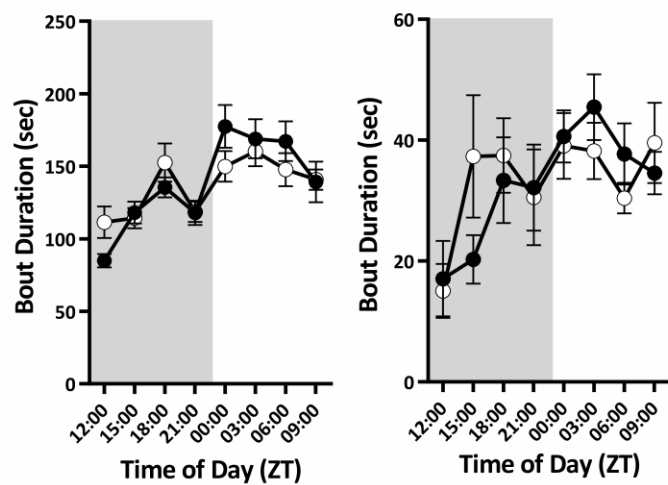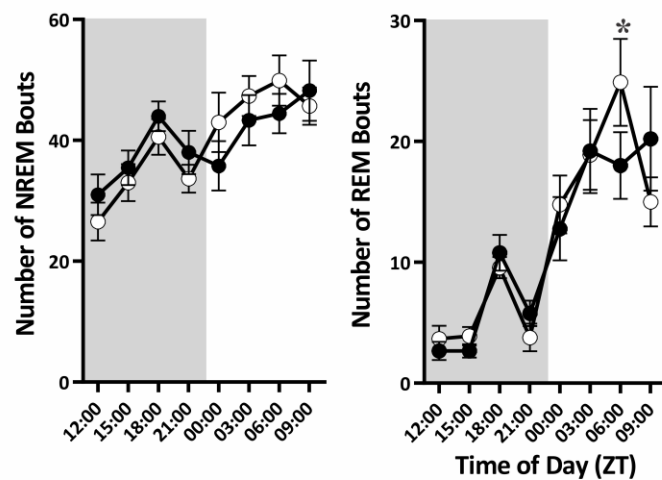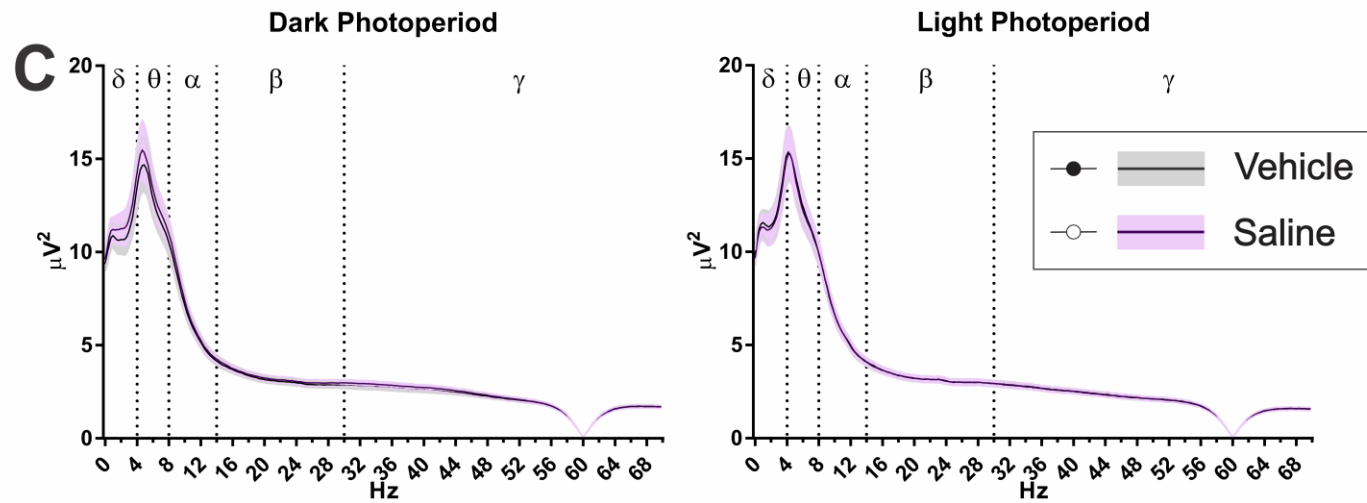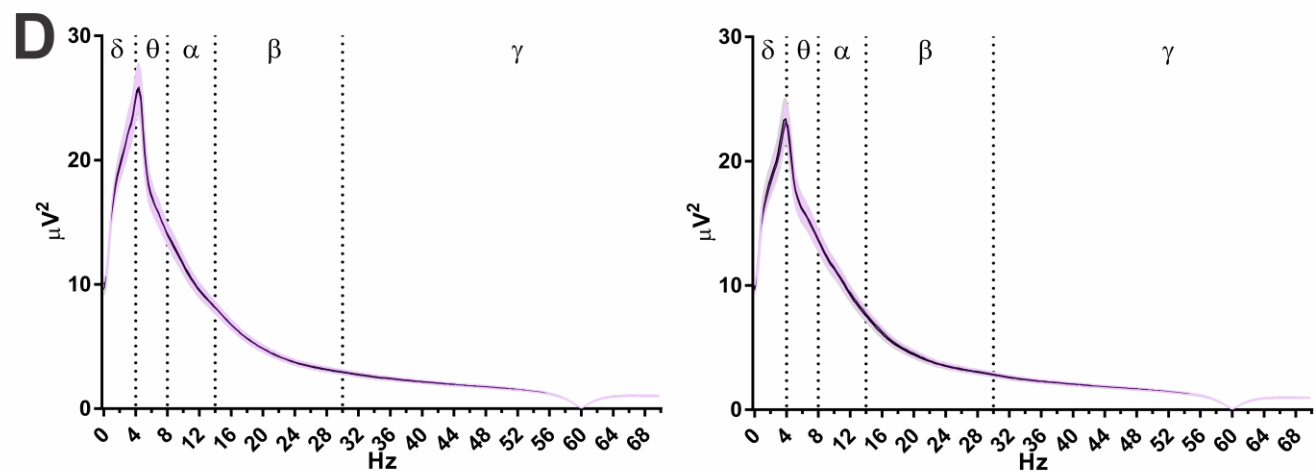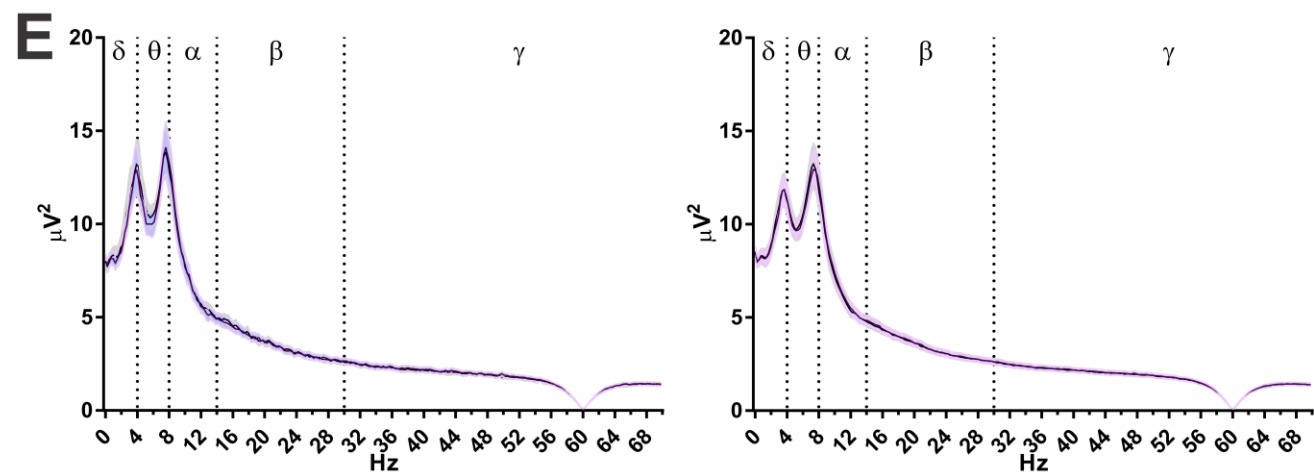

Supplement: S2 Fig — Data are from experiment with CP47 (N = 9), where subjects were administered a saline injection i.p. the day prior to the vehicle injection. The vehicle data depicted here are the same as those depicted in Fig 4. A, NREM sleep time or architecture. Top graph: Percent time in NREM was not affected by the vehicle solution. Middle graph: The duration of NREM bouts was not affected by vehicle injection. Bottom graph: The number of NREM bouts was not affected by the vehicle solution. B, REM sleep time and architecture. Top graph: The percent time in REM was not affected by vehicle injection. Middle graph: The duration of REM bouts was not affected by vehicle injection. Bottom graph: For the number of REM bouts, there was an overall interaction (treatment x time of day within photoperiod, F(6,98.77) = 2.63, p = 0.021), nested interaction (time of day within photoperiod, F(6, 95.49) = 6.56, p < 0.001), and a main effect of treatment (F(1, 74.92) = 82.37, p < 0.001). Overall, the vehicle solution did not alter the number of REM bouts when data were collapsed across the day or when comparisons were made with data collapsed within either LP or DP. However, there was a slight reduction in the number of REM bouts at one point in the LP (ZT06-09: t(82.02) = -2.10, p = 0.039). Given the small effect size, limited to only one measure of REM architecture in a very restricted timeframe many hours after the injection, we conclude that the vehicle solution used in this study has little or no effect on sleep in C57BL/6 mice. C-E, There were no obvious changes in EEG power spectra following vehicle administration. C, Power spectra from wake epochs. D, Power spectra from NREM epochs. E, Power spectra from REM epochs. In A & B, Grey shaded regions indicate the DP, and symbols/bars represent means±SEM across all subjects for each 3 Hr time bin. (PDF) [file pone.0152473.s003.pdf]

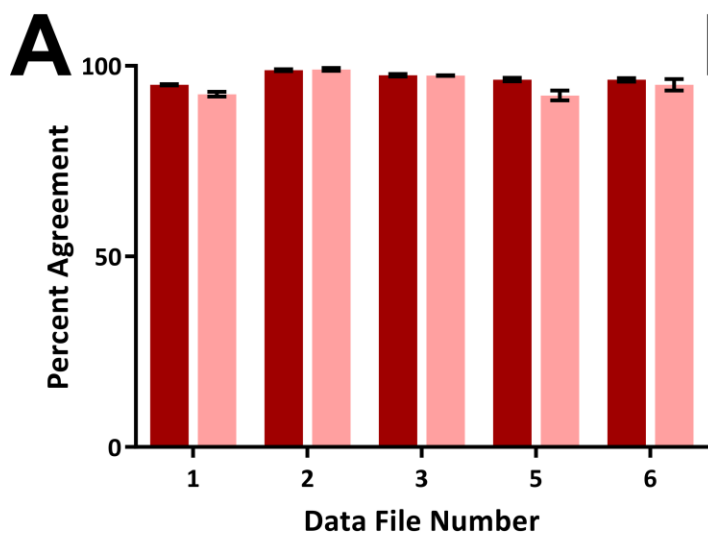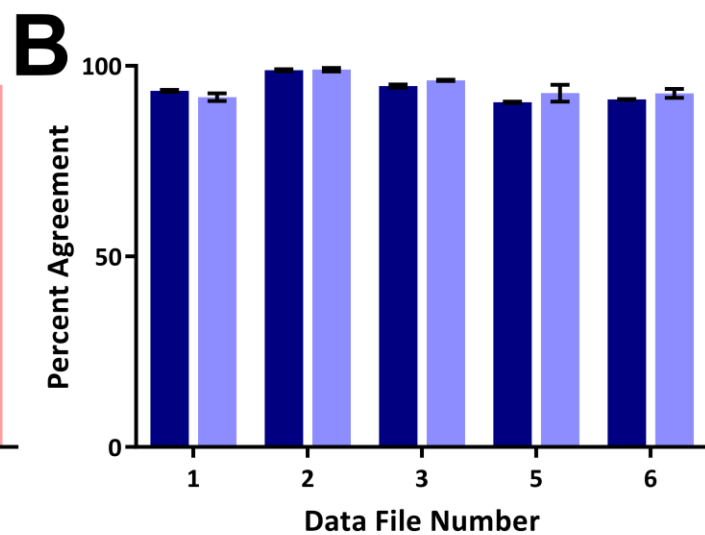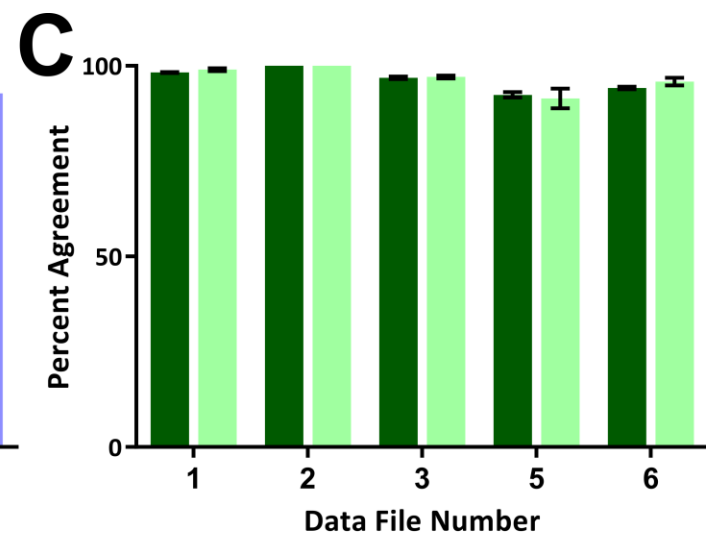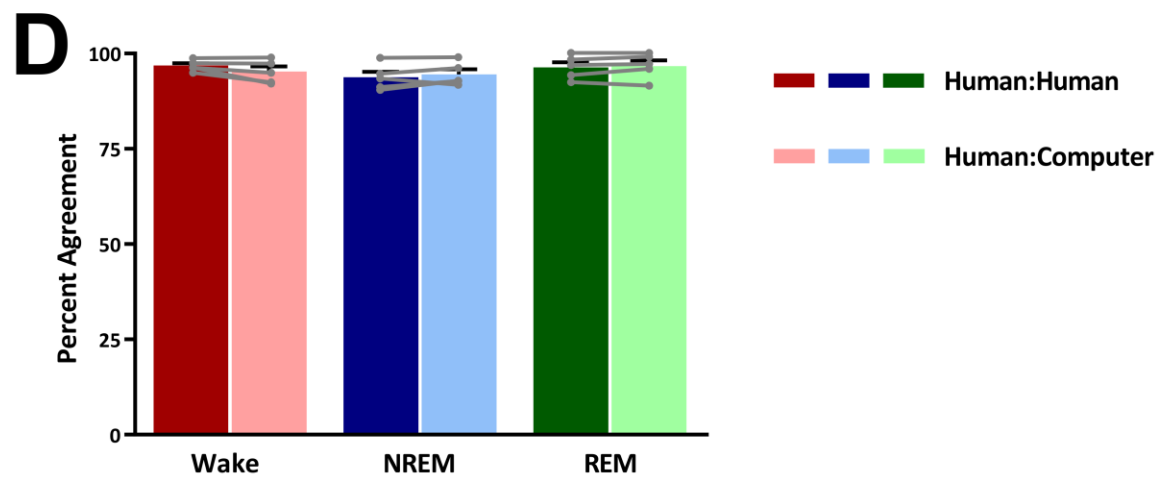

Supplement: S3 Fig — To compute percent agreement by vigilance state, each human’s score and the computer’s score were compared against a template derived from human scored data. This meant that for each human there were two possible templates, and these values were averaged together yielding one human:human percent agreement score per human scorer per each of 5 data files used (the data file with corrupt EMG channel used in overall percent agreement, Fig 1C, was excluded for this analysis as it was unscorable). Thus, for each data file there were three human:human measures and three computer:human measures. The state-specific percent agreement was calculated as the fraction of epochs where the scorer and template agreed that epochs were or were not a target state over the total number of epochs (% agreement = 100% x [agree State + agree not State]/total number of epochs). For each state (wake, NREM, and REM) a two-way repeated measures ANOVA was performed with data file as a repeated factor and scoring comparison (human:human vs. computer:human) as a between-groups factor. A, Results for percent agreement for wake epochs. There was an interaction between scoring type and datafile (F(4,16) = 3.82, p = 0.023) and a main effect of data file (F(4,16) = 21.92, p < 0.001). However, there was a only a slight reduction in percent agreement for data file number 5 in the human:computer (t(20) = 4.14, p = 0.003). B, Results for percent agreement for NREM epochs. There was only a main effect of data file (F(4, 16) = 28.53, p < 0.001). C, Results for percent agreement for REM epochs. There was only a main effect of data file (F(4, 16) = 33.18, p < 0.001). D, Shows data collapsed across scorers and the results of a paired comparison by data file. Grey, connected points superimposed on the bar graph indicate mean human:human and computer:human agreement for each data file. A paired t-test was performed for each vigilance state. Percent agreement was not significantly different for human:human vs huma [file pone.0152473.s004.pdf]

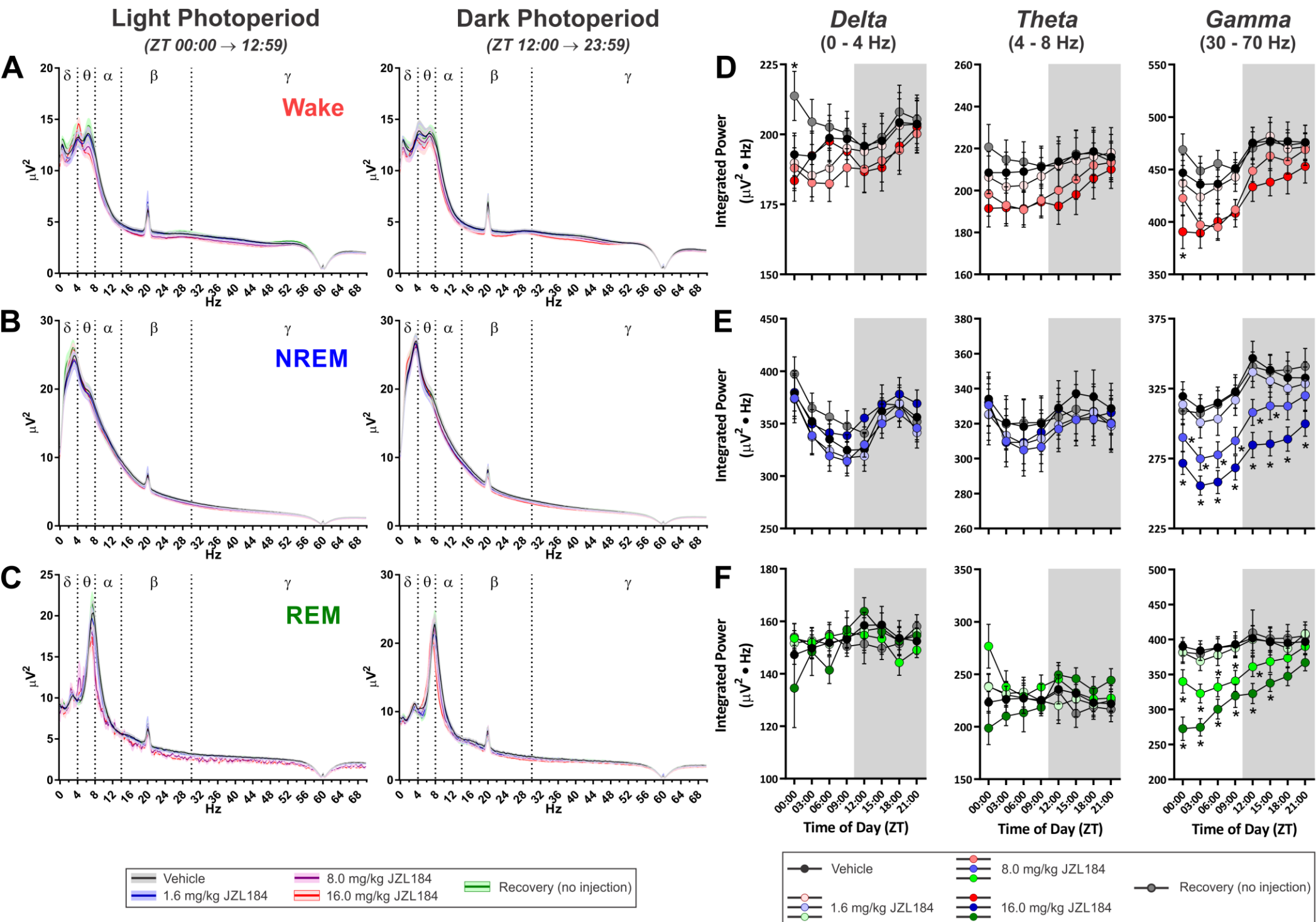

Supplement: S4 Fig — A-C, Average power spectra for epochs of different vigilance states across the entire LP (left hand) and DP (right hand). Solid lines denote means and shaded region around lines denotes SEM. A, Wake. B, NREM. C, REM. D-F, Change over the day in summated power in different frequency bandwidths from the power spectra: delta (left hand column), theta (middle column), and gamma (right hand column). D, Wake epochs. Left panel: For wake delta, there was an overall interaction (treatment x time of day within photoperiod, F(24, 261.30) = 2.08, p = 0.003), nested interaction (time of day within photoperiod, F(6, 242.10) = 9.33, p < 0.001), and a main effect of photoperiod (F(1, 134.75) = 6.88, p = 0.010). The only time point that significantly deviated from vehicle was during the first 3 Hr of the recovery day, when there was an increase in delta power (t(215.67) = 2.86, p = 0.018). Middle panel: No effect of JZL184 on wake theta power. Right panel: For wake gamma power, there was a nested interaction (time of day within photoperiod, F(6, 253.19) = 6.08, p < 0.001) and main effects of both treatment (F(4,67.43) = 3.21, p = 0.018) and photoperiod (F(1, 179.51) = 115.90, p < 0.001). Specifically, 16 mg/kg JZL reduced gamma power during the first 3 Hr of the LP (ZT 00–03: t(57.18) = -2.68, p = 0.038). E, NREM epochs. Left panel: For NREM delta power, there was no effect of JZL treatment. Middle panel: For NREM theta power, there was an overall interaction (treatment x time of day within photoperiod, F(24, 268.23) = 1.64, p = 0.033), a nested interaction (time of day within photoperiod, F(6, 238.31) = 20.36, p < 0.001), and a main effect of photoperiod (F(1, 159.84) = 85.90, p < 0.001). However, there were no specific time points where JZL184 significantly altered NREM theta power relative to vehicle. Right panel: For NREM gamma power, there was an overall interaction (treatment x time of day within photoperiod, F(24, 267.36) = 2.46, p < 0.001), a nested interaction (time of day [file pone.0152473.s005.pdf]

**Dark Photoperiod**  
(ZT 12:00 → 23:59)

**Light Photoperiod**  
(ZT 00:00 → 11:59)

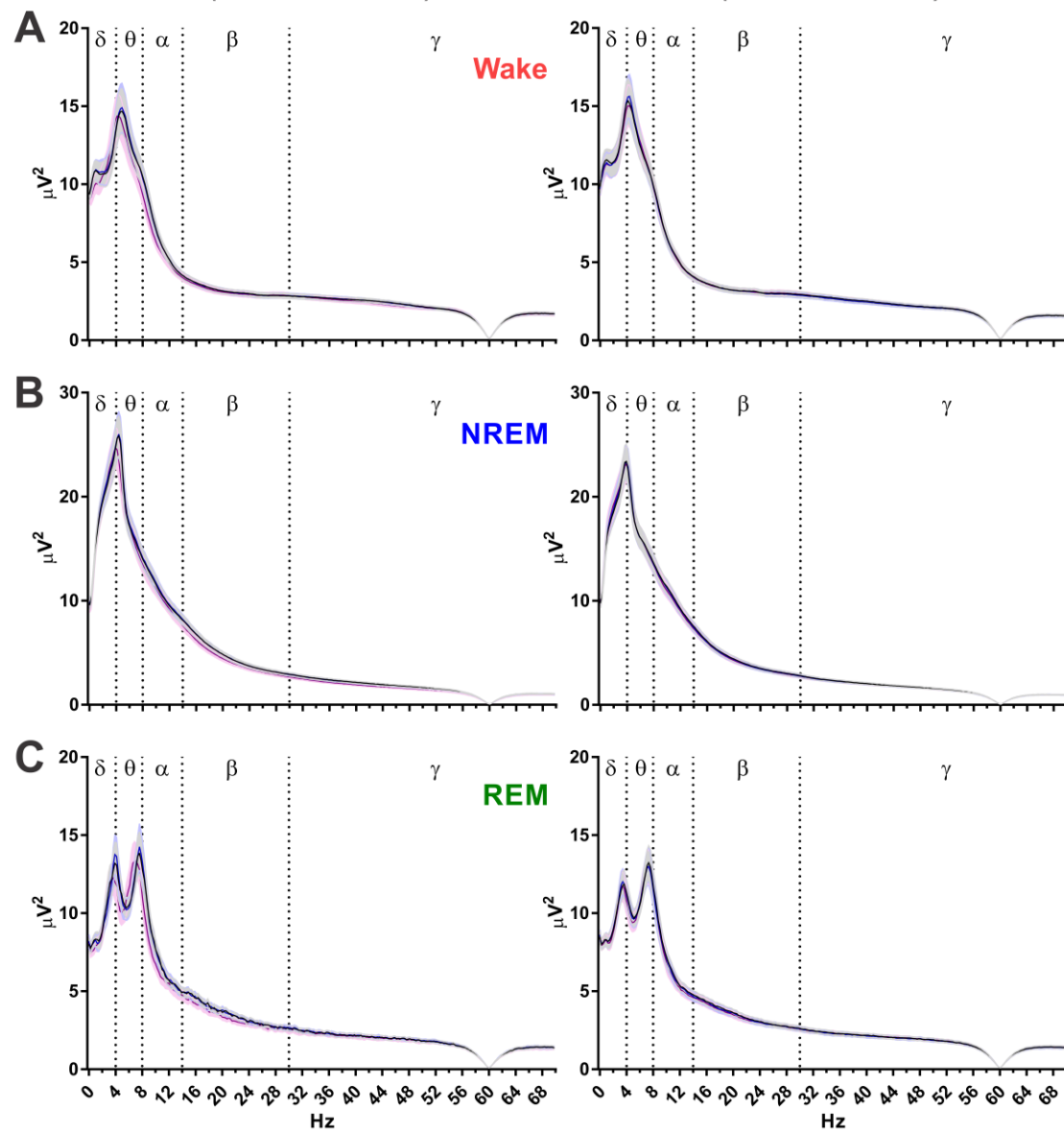

— Vehicle — 0.1 mg/kg CP47,497 — 1.0 mg/kg CP47,497

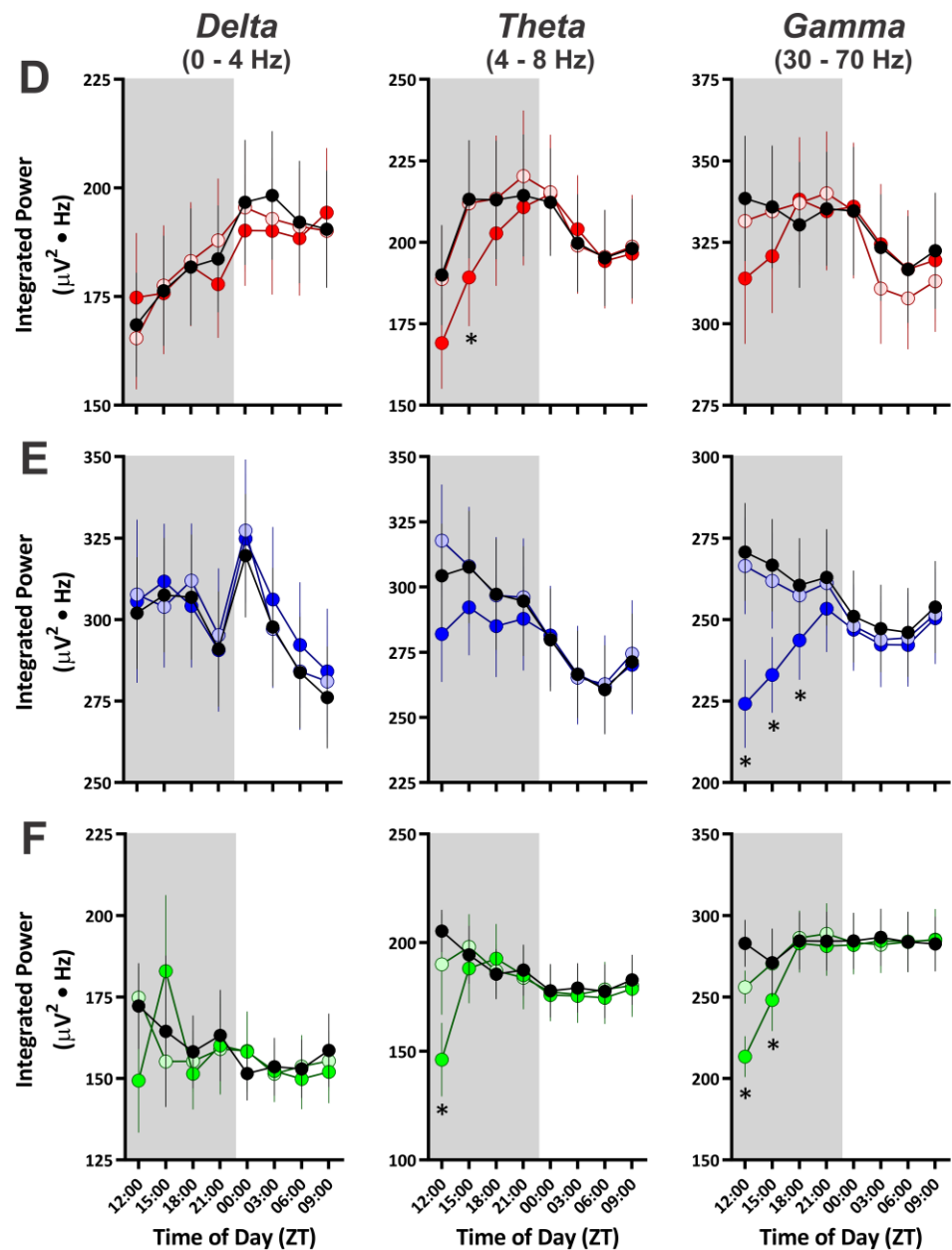

— Vehicle — 0.1 mg/kg CP47,497 — 1.0 mg/kg CP47,497

Supplement: S5 Fig — A-C, Average power spectra for epochs of different vigilance states across the entire DP (left hand) and LP (right hand). Solid lines denote means and shaded region around lines denotes SEM. A, Wake. B, NREM. C, REM. D-F, Change over the day in summated power in different frequency bandwidths from the power spectra: delta (left hand column), theta (middle column), and gamma (right hand column). D, Wake Epochs. Left panel: CP47 had no effect on wake delta power. Middle panel: For wake theta power, there was a significant overall interaction (drug x time of day within photoperiod, F(15, 180.83) = 9.73, p < 0.001) with a significant reduction in wake theta at only a single time point during the dark photoperiod (ZT 15–18: t(186.39) = -2.29, p = 0.047). Right panel: For wake gamma, there was a significant overall interaction (drug x time of day within photoperiod, F(15, 179.99) = 3.04, p < 0.001) with a main effect of photoperiod (F(1, 135.02) = 6.86, p = 0.010). However, there was not a difference at any specific time point between low or high dose CP47 and vehicle. E, NREM Epochs. Left panel: For NREM delta there was an overall interaction (drug x time of day within photoperiod, F(15, 179.22) = 3.07, p < 0.001). However, there were no pair-wise differences at any time point between high or low dose CP47 and vehicle. Middle panel: For NREM theta power, there was an overall interaction (F(15, 180.85) = 2.79, p = 0.001) with main effect of photoperiod (F(1, 157.14) = 50.99, p < 0.001). However, there were no pair-wise difference between drug treatment conditions and vehicle. Right panel: For NREM gamma, there was an overall interaction (drug x time of day within photoperiod, F(15, 181.48) = 3.50, p < 0.001), secondary interaction (drug x photoperiod, F(2, 184.76) = 8.82, p < 0.001) with main effects of drug treatment (F(2, 175.98) = 7.13, p < 0.001) and photoperiod (F(1, 174.89) = 11.39, p = 0.001). Specifically, 1.0 mg/kg CP47 reduced NREM gamma power during the first 9 [file pone.0152473.s006.pdf]

**A**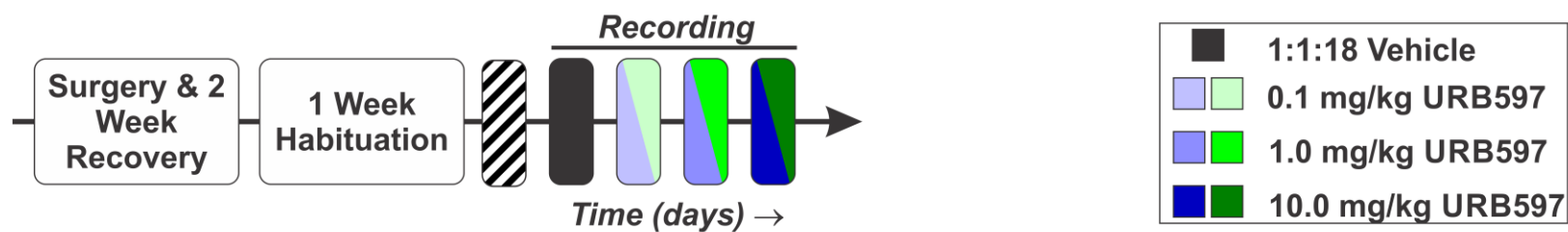**B**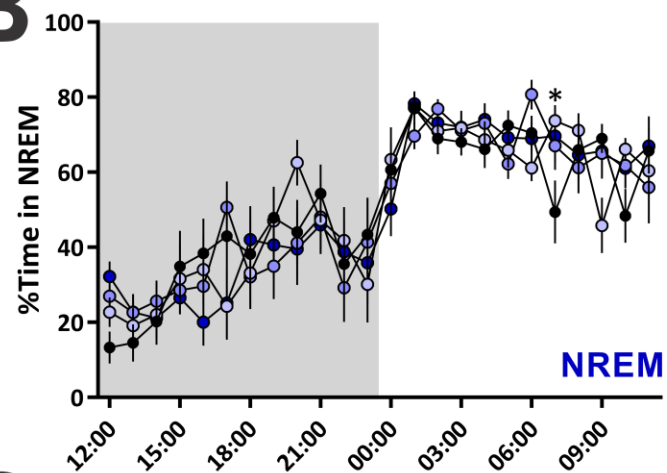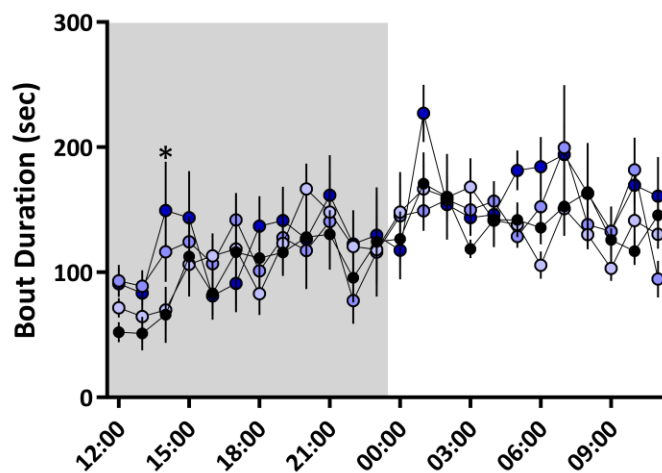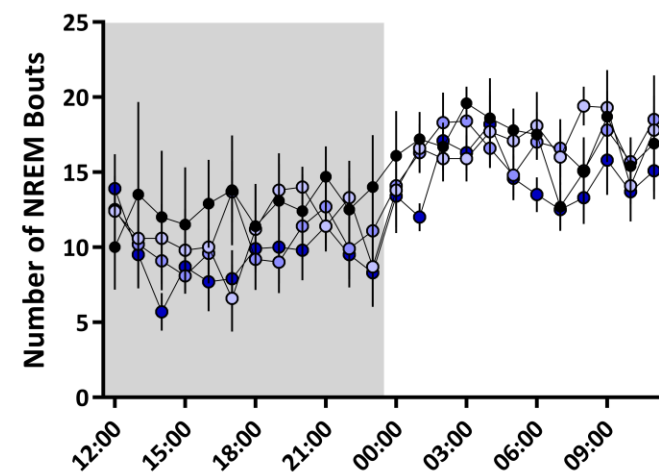**C**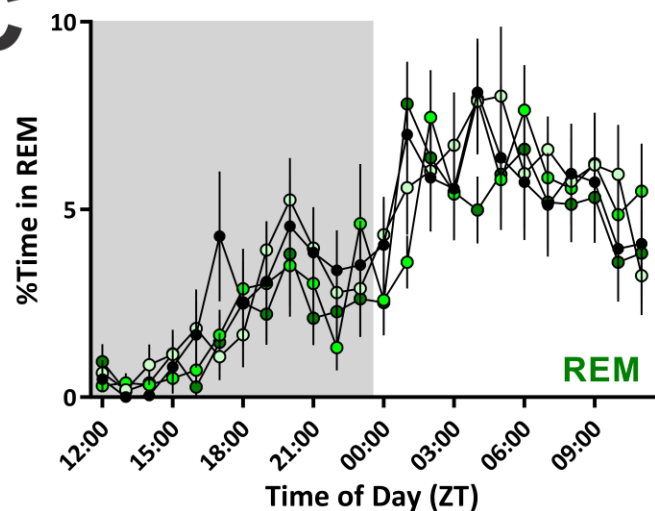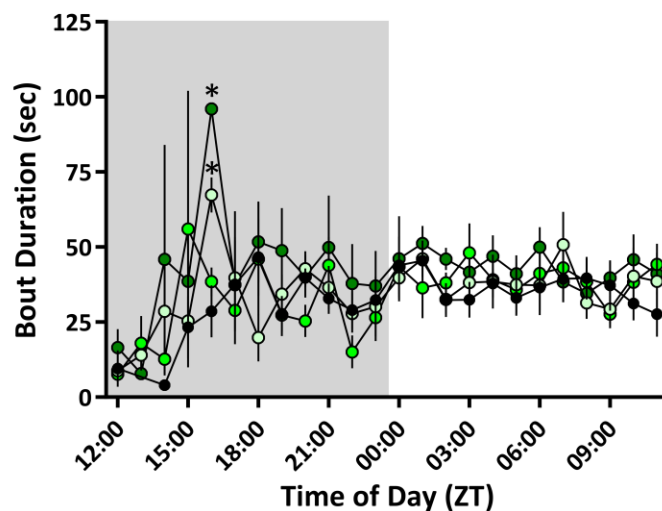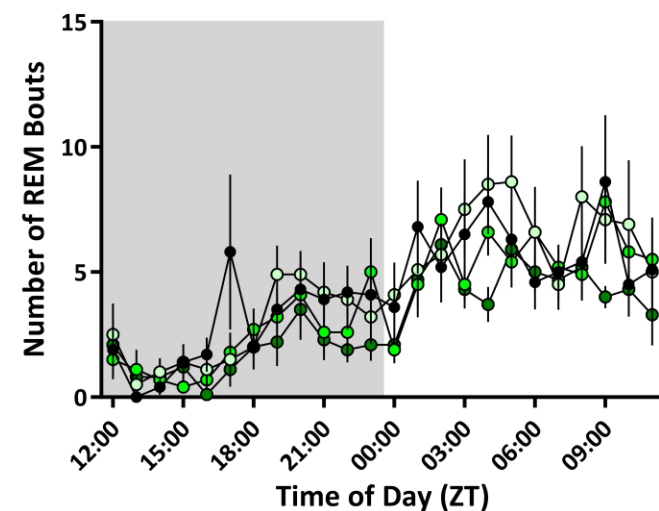

Supplement: S6 Fig — A, Diagram of experimental protocol for recording sleep after administration of the reversible FAAH inhibitor, URB597. B, Effect of URB597 on NREM sleep time and architecture. Left Graph: There was no effect of URB on NREM sleep time. Middle Graph: For NREM bout duration, there was a nested interaction (time of day within photoperiod, F(22,609.62) = 3.04, p < 0.001) and main effects of treatment (F(4, 312.20) = 56.45, p < 0.001) and photoperiod (F(1,365.61) = 80.16, p < 0.001). 10.0 mg/kg URB produced and overall increase in NREM bout duration (t(303.24) = 3.40, p = 0.003), specifically during the third hour of the DP (ZT14-15: t(1002.87) = 2.82, p = 0.020). Right Graph: For the number of NREM bouts, there was a nested interaction (time of day within photoperiod, F(22,690.70) = 1.60, p = 0.041) and main effects of both treatment (F(4,198.00) = 2.97, p = 0.021) and photoperiod (F(1,253.69) = 88.28, p < 0.001). Overall, 10.0 mg/kg URB reduced the number of NREM bouts (t(194.74) = -2.84, p = 0.020), but there were no differences at specific time points. C, Effect of URB597 of REM sleep time and architecture. Left Graph: There was no effect of URB on REM sleep time. Middle Graph: For REM bout duration, there was a nested interaction (time of day within photoperiod, F(22,478.80) = 2.31, p = 0.001) and main effects of drug treatment (F(4,249.61) = 3.80, p = 0.005) and photoperiod (F(1,302.99) = 11.14, p = 0.001). Overall, 10.0 mg/kg URB increased REM bout duration, specifically during the fifth hour of the DP (ZT16-17: t(726.45) = 2.54, p = 0.045). Right Graph: For the number of REM bouts, there was a nested interaction (time of day within photoperiod, F(22,266.50) = 4.84, p = 0.001), and main effects of treatment (F(4, 266.50) = 4.84, p = 0.001) and photoperiod (F(1,318.73) = 152.30, p < 0.001). Overall, 10.0 mg/kg URB reduced the number of REM bouts (t(263.14) = -2.90, p = 0.016), but there were several specific time points throughout the day when 10.0 mg/kg URB decreas [file pone.0152473.s007.pdf]

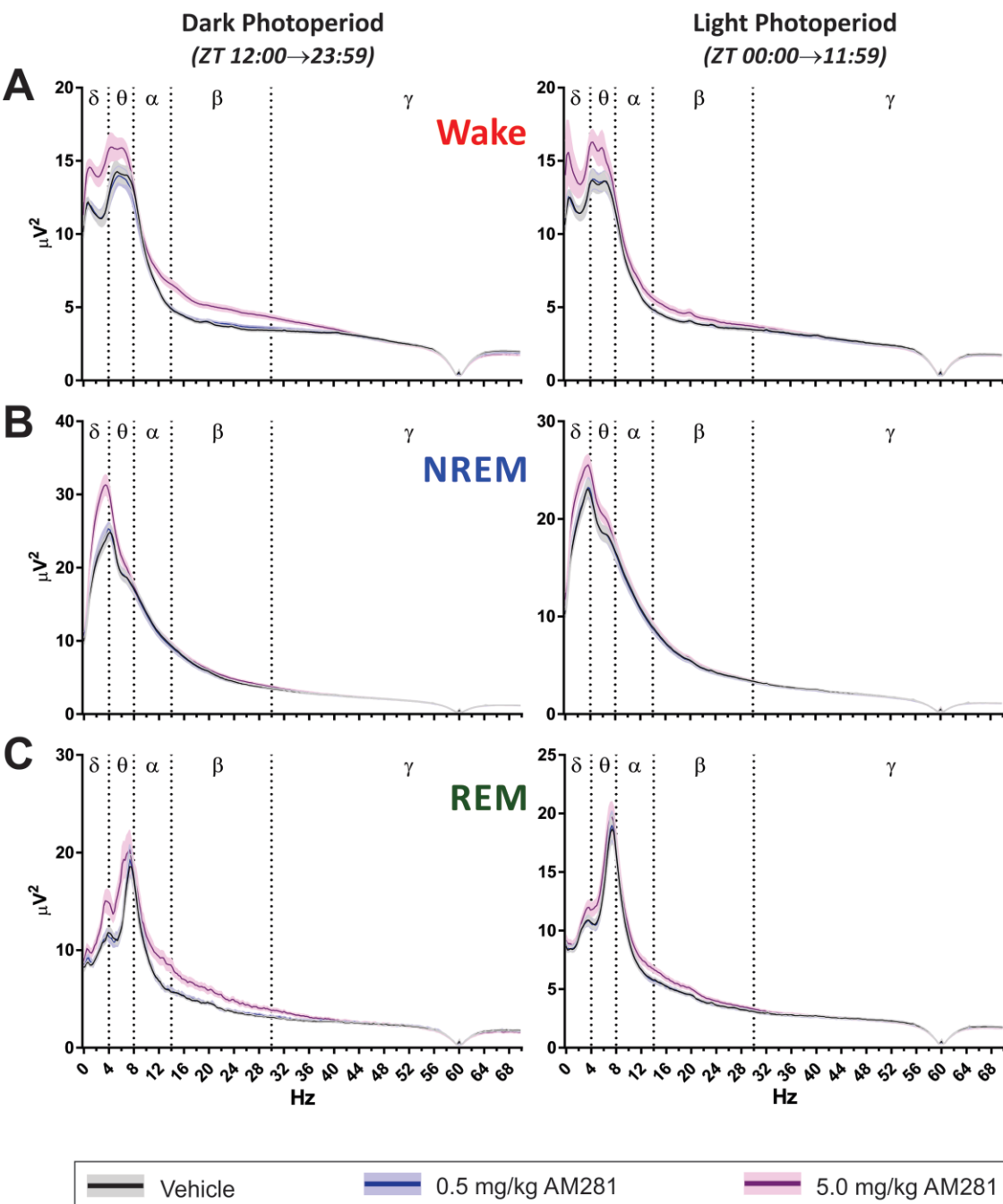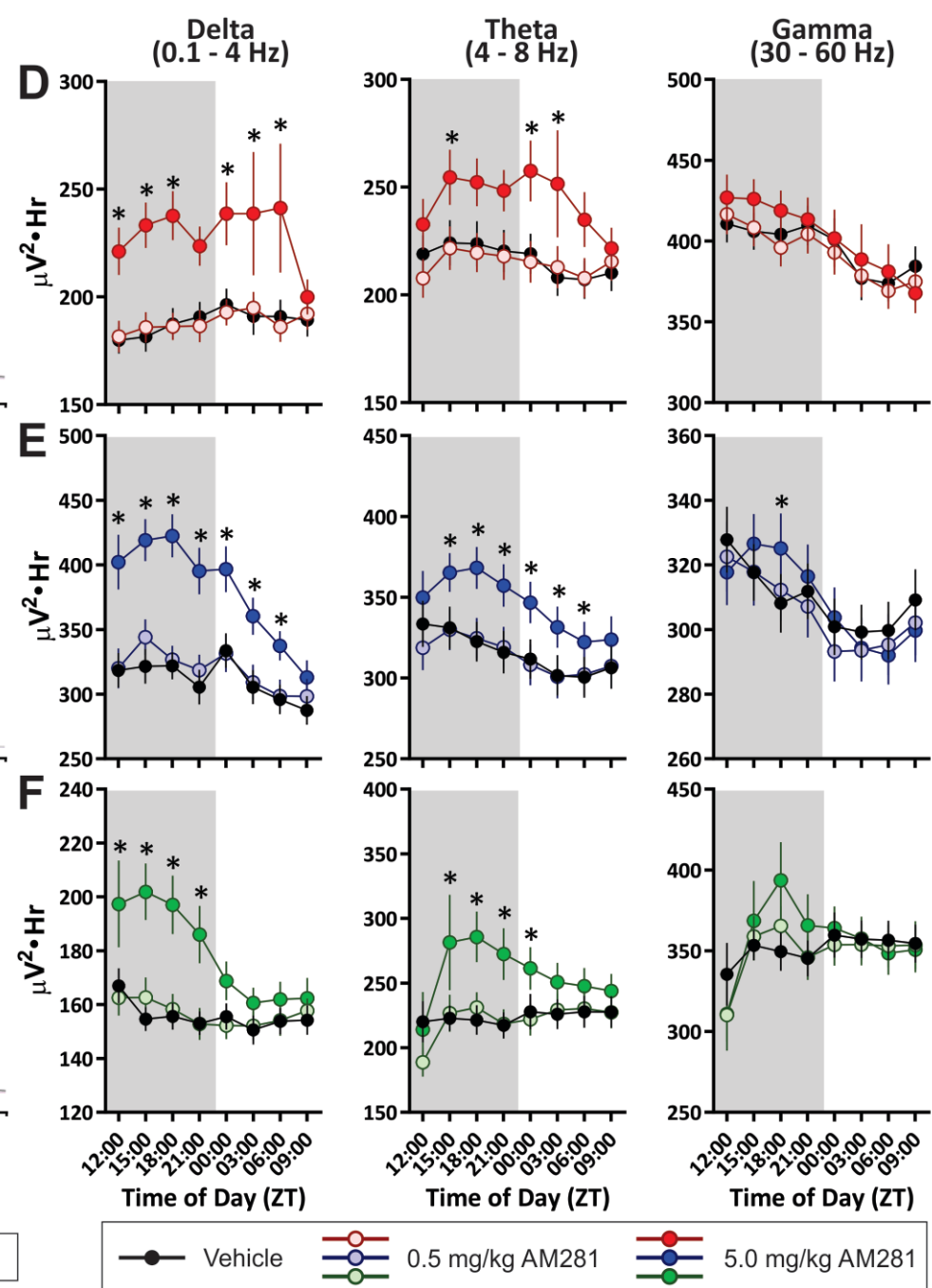

Supplement: S8 Fig — A—C, Power spectra from different vigilance states averaged over 12 Hr light/dark photoperiods. Dark lines represent group means and shaded regions surrounding the lines represent SEM. A, Wake. B, NREM. C, REM. D–F, Quantification of delta (0–4 Hz), theta (4–8 Hz), and gamma (30-60Hz) bandwidths of power spectra across the three vigilance states. D, Wake epochs. Left panel: For delta power, there was a significant overall interaction (drug x time of day within photoperiod, F(12, 232.82) = 2.05, p = 0.021) with a main effect of drug treatment (F(2, 61.15) = 12.80, p < 0.001). 5.0 mg/kg AM281 significantly increased delta power during wake epochs across most of the experiment (ZT 12–21 & 00–09; t(144.52) ≥ 2.71, p ≤ 0.015). Middle panel: For wake theta power, there was a nested interaction (time of day within photoperiod, F(6, 237.52) = 3.78, p = 0.001) with a main effect of drug treatment (F(2, 72.91) = 7.53, p < 0.001). Theta power was increased at one time point during the dark photoperiod (ZT 15–18: t(159.69) = 2.18, p = 0.049) and the first half of the light photoperiod (ZT 00–06: t(159.69) ≥ 2.87, p ≤ 0.009). Right panel: There was no effect of treatment on power in the gamma bandwidth. E, NREM epochs. Left panel: For NREM delta power, there was a significant overall interaction (drug x time of day within photoperiod, F(12, 243.32) = 2.77, p = 0.002), a secondary interaction (drug x photoperiod, F(2, 248.67) = 6.84, p = 0.001), a nested interaction (time of day within photoperiod, F(6, 232.06) = 30.63, p < 0.001), and main effects of both drug treatment (F(2, 104.70) = 35.00, p < 0.001) and photoperiod (F(1, 156.74) = 45.51, p < 0.001). Specifically, high dose AM281 increased NREM delta power across most time bins (ZT 12–21: t(195.82) ≥ 2.89, p ≤ 0.009). Middle panel: For NREM theta power, there was a significant overall interaction (drug x time of day within photoperiod, F(12, 247.79) = 2.40, p = 0.006), a nested interaction (time of day within photoperiod, F(6 [file pone.0152473.s009.pdf]

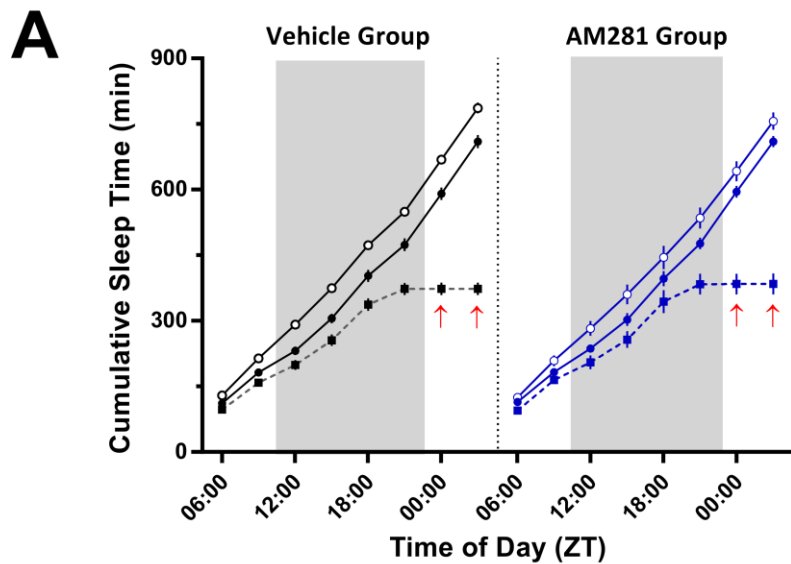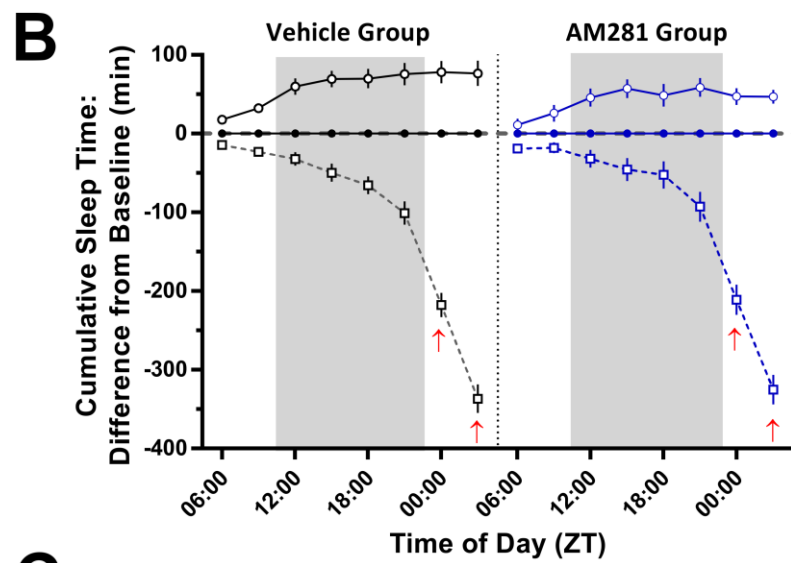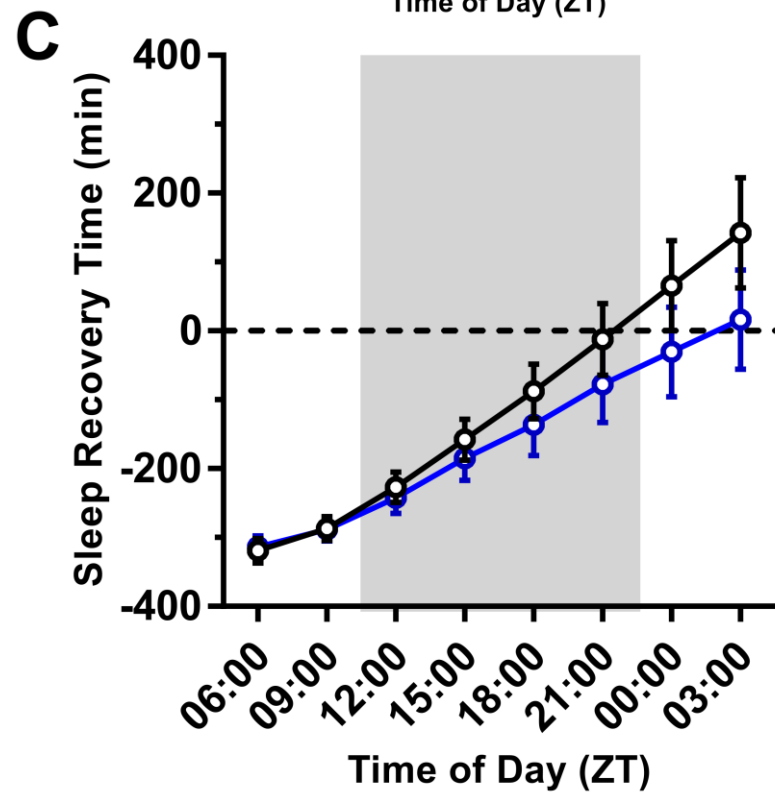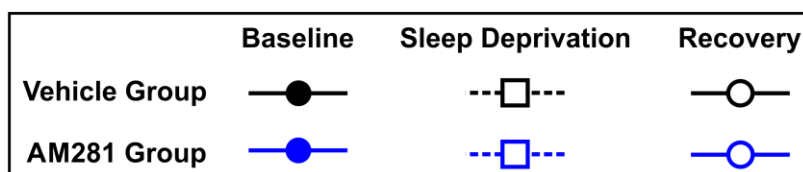

Supplement: S9 Fig — To determine if sleep homeostatic mechanisms were altered by administration of the CB1 antagonist immediately following 6 Hr of acute TSD, we determined the sleep deficit incurred during TSD and computed the recovery from this deficit. A, First the cumulative NREM sleep time was calculated for each subject in both groups over sequential 3 Hr bins across three phases of the experiment: baseline vehicle administration, sleep deprivation, and the recovery day immediately following sleep deprivation. B, Second, each subject’s baseline cumulative sleep was subtracted from each of these three curves. The baseline day is only shown here to demonstrate this normalization. C, The sleep debt incurred by each animal after sleep deprivation was taken as the last bin of the baseline normalized cumulative sleep on the deprivation day. This value was separately calculated for each subject and was subtracted from each point of the baseline normalized cumulative sleep plot for the recovery day (open symbols panel B). This yielded the two curves depicted in panel C for the recovery from sleep debt incurred during TSD. A two-way repeated measures ANOVA was performed on the NREM recovery data shown in panel C with treatment group as a between-groups factor and time of day as a within-subjects repeated measure. There was a significant main effect of time of day (F(7, 126) = 51.62, p < 0.001), but there was neither an interaction (F(7, 126) = 1.35, p = 0.23) nor a main effect of treatment (F(1, 18) = 0.75, p = 0.40), suggesting that both groups recovered similarly from TSD. In panels A and B, the red arrows denote the two time bins when the sleep deprivation device was activated. There was generally lower overall sleep for most of sleep deprivation day in both groups, even prior to activation of the rotor. However, this is not surprising given that the subjects had just been placed into the deprivation chambers and were likely habituating to the new environment. Symbols/Bars represent me [file pone.0152473.s010.pdf]

**A**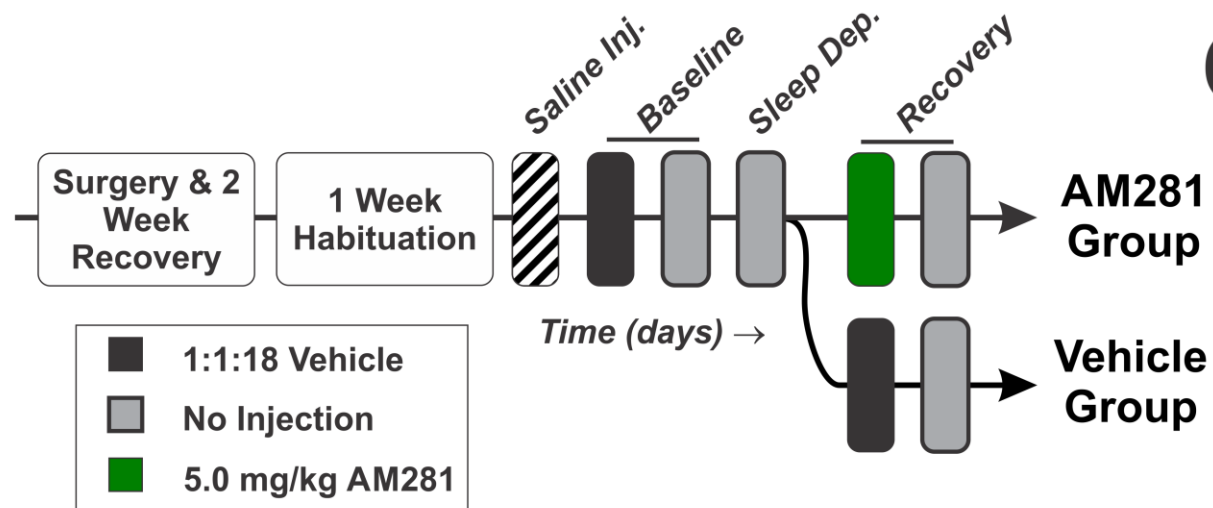**B**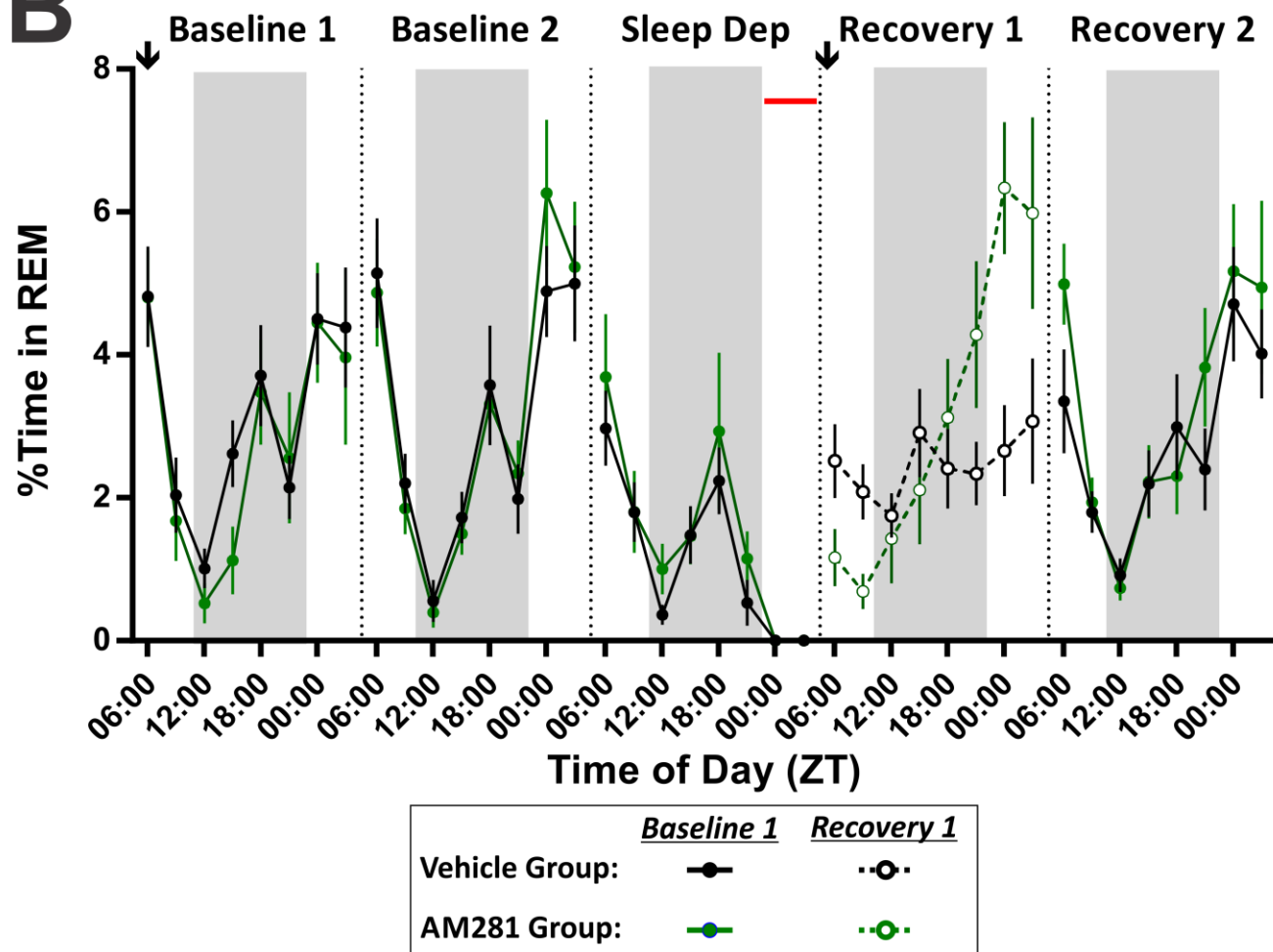**C**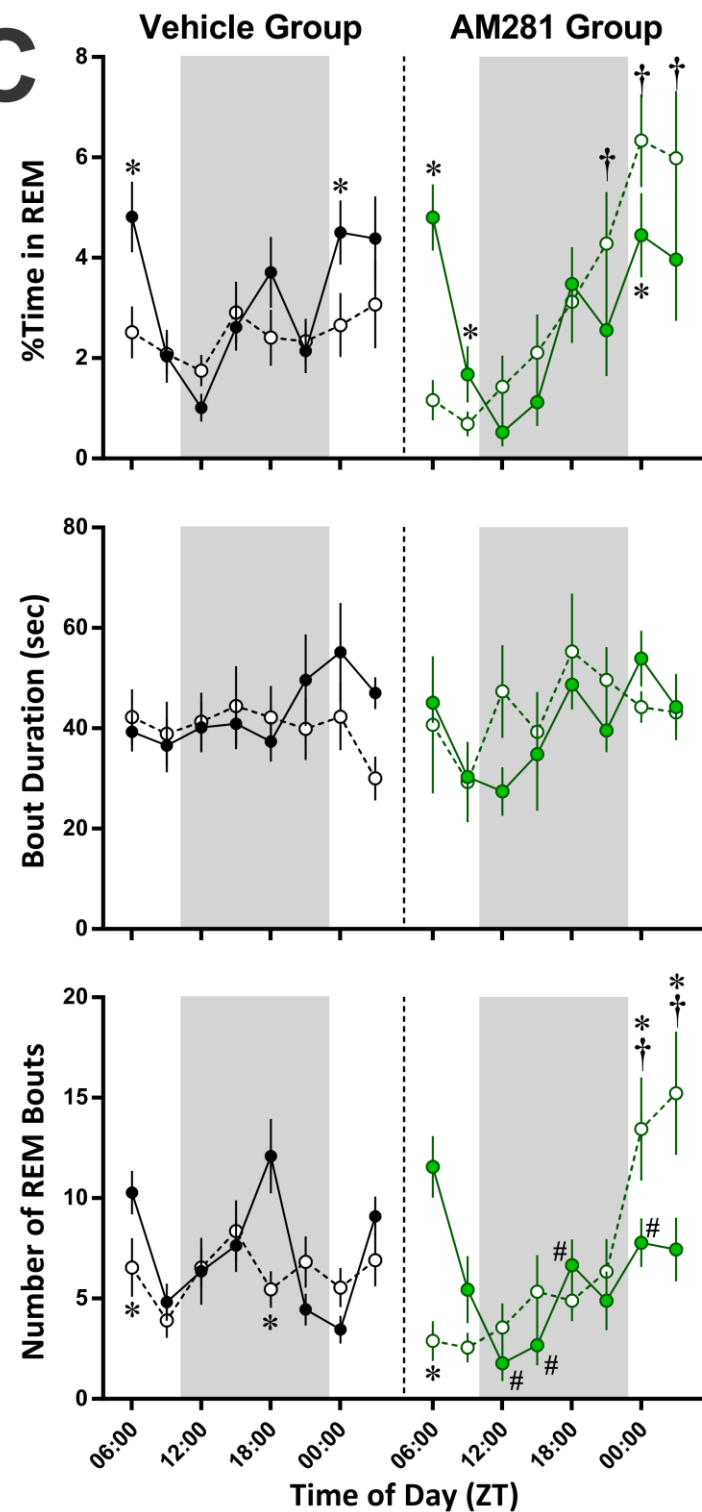

Supplement: S10 Fig — A, Diagram of experimental protocol repeated here for clarity with different color coding to indicate measures reflect REM sleep parameters. B, Overall fluctuation in REM sleep throughout the entire experiment. Downward facing arrows denote times at which injections were given. The red horizontal line indicates the time at which the sleep deprivation chambers were activated. C, Comparisons within and between treatment groups across the first baseline and recovery days. Top Graph: Percent time in REM sleep. There was a significant overall interaction (treatment group x time of day within photoperiod within experimental phase, F(24, 270) = 7.01, p < 0.001), a secondary interaction (treatment group x photoperiod within experimental phase, F(3, 270) = 3.56, p = 0.015), a tertiary interaction (treatment group x experimental phase, F(1, 270) = 6.71, p = 0.010), and a main effect of photoperiod (F(1, 270) = 32.26, p < 0.001). For the vehicle group, there was significantly less REM sleep overall on the recovery day compared to baseline (t(270) = -2.64, p = 0.009), but there was not an overall difference between groups for the amount of REM on either the baseline or recovery days. However, the AM281 group had significantly more REM sleep than vehicle treated mice towards the end of the recovery day (ZT 21–06: t(98.63) ≥ 2.01, p ≤ 0.047). Compared to their baseline sleep, both vehicle and AM281 treated mice had significantly less REM during the first 3 Hr of the recording (ZT 06–09: t(270) ≤ -3.13, p ≤ 0.002), but the AM281 group went on to exhibit a REM rebound late in the recording (ZT 21–06: t(270) ≥ 2.12, p ≤ 0.035), while the vehicle group continued to have less REM than their baseline (ZT 00–03: t(270) = -2.51, p = 0.013). Middle Graph: REM bout duration. There was no effect of either sleep deprivation or AM281 treatment on REM bout duration in this experiment. This is possibly because the estimates of REM bout duration were taken from a small number of REM bouts for ea [file pone.0152473.s011.pdf]

**A**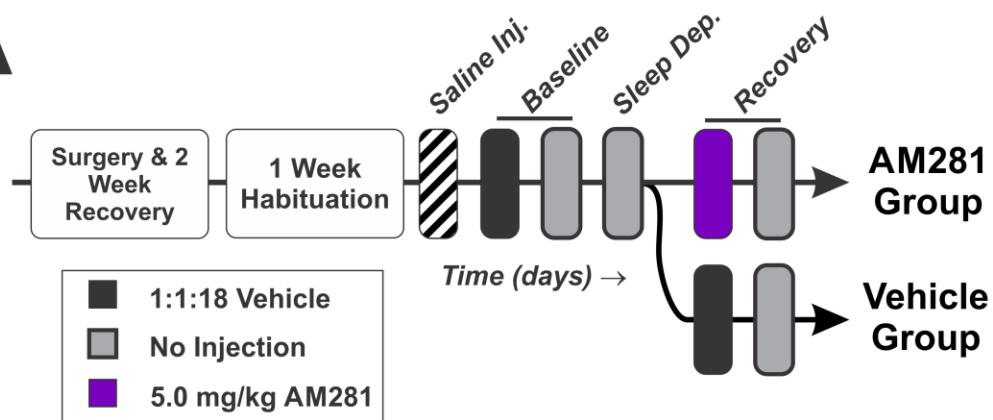**B**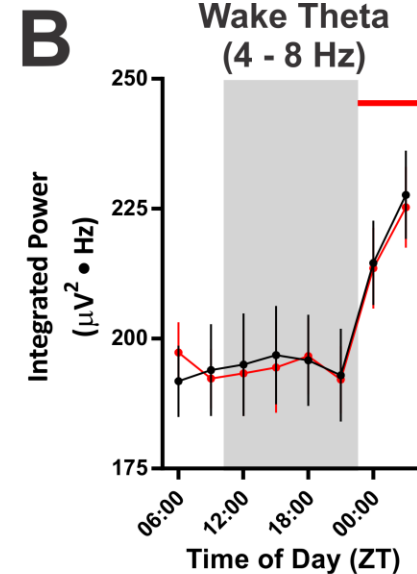**C**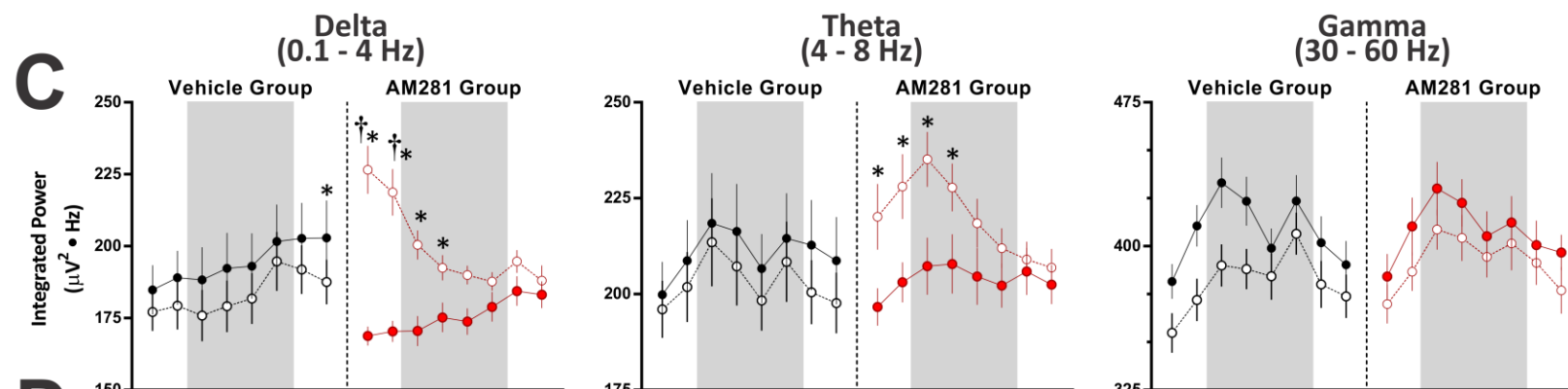**D**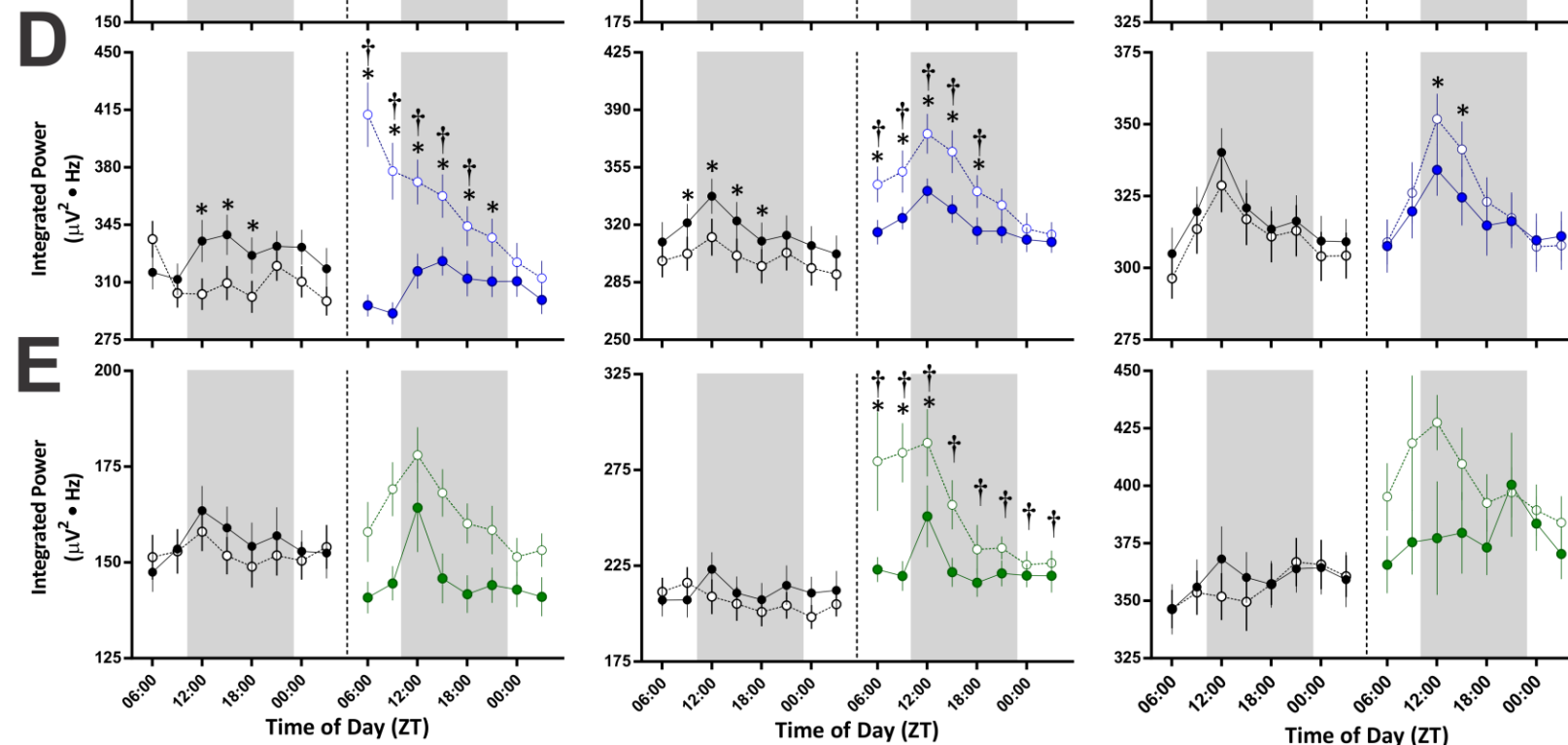**E**

Supplement: S11 Fig — A, Diagram of experimental protocol for sleep deprivation. B, Wake theta power during sleep deprivation. Horizontal red bar indicates sleep deprivation session during first 6 Hr of the LP. C, Wake epochs. Left panel: For wake delta, there was an overall interaction (treatment group x time of day within photoperiod within experimental phase, F(12, 259.47) = 12.27, p < 0.001) and a secondary interaction (treatment group x experimental phase, F(1, 192.08) = 16.66, p < 0.001). For the first 6 Hr following TSD, wake delta was increased by AM281 administration relative to delta power measurements in the vehicle group (ZT06-12: t(35.02) ≥ 3.20, p ≤ 0.003). Additionally, within-group comparisons found that AM281 elevated wake delta across the first 12 Hr of recovery from TSD (ZT06-18: t(255.42) ≥ 2.09, p ≤ 0.038). In contrast, wake delta power was reduced in the vehicle treated group but only during the last 3 Hr of the recovery (ZT03-06: t(255.42) = -2.04, p = 0.042). Middle panel: For wake theta, there was an overall interaction (treatment group x time of day within photoperiod within experimental phase, F(12, 259.47) = 12.27, p < 0.001) and a secondary interaction (treatment group x experimental phase, F(1, 185.67) = 6.26, p = 0.013). There were no pair-wise differences between groups during baseline or recovery. However, treatment with AM281 increased wake theta relative to baseline during the first 12 Hr of recovery (ZT06-18: t(252.32) ≥ 2.18, p ≤ 0.030). For the vehicle group, there were no pair-wise differences in wake theta between baseline and recovery. Right panel: For wake gamma, there was a nested interaction (time of day within photoperiod within experimental phase, F(12, 255.50) = 13.77, p < 0.001) and a main effect of experimental phase (F(1, 139.37) = 9.85, p = 0.002). Across treatment groups, there was an overall reduction of wake gamma power during recovery from TSD relative to baseline (t(139.37) = -3.14, p = 0.002). D, NREM epochs. Left panel: For NREM d [file pone.0152473.s012.pdf]
