# Supplementary material for: Endocannabinoid Signaling Regulates Sleep Stability
Source: PLoS One. 2016 Mar 31;11(3):e0152473. doi: 10.1371/journal.pone.0152473 (PMC4816426; doi:10.1371/journal.pone.0152473)
Supplement: S7 Fig — A-C, Average power spectra for epochs of different vigilance states across the entire DP (left hand) and LP (right hand). Solid lines denote means and shaded region around lines denotes SEM. A, Wake. B, NREM. C, REM. D-F, Change over the day in summated power in different frequency bandwidths from the power spectra: delta (left hand column), theta (middle column), and gamma (right hand column). D, Wake epochs. Left panel: For wake delta power, there was an overall interaction (drug x time of day within photoperiod, F(12, 182.27) = 2.47, p = 0.005) and a secondary interaction (drug x photoperiod, F(2, 185.16) = 3.62, p = 0.029) with a main effect of photoperiod (F(1, 176.67) = 34.22, p < 0.001). Specifically, there was increased wake delta power during the first half of the dark photoperiod on the recovery day (ZT 12–18: t(191.85) ≥ 2.94, p ≤ 0.007). Middle panel: For wake theta power, there was an overall interaction (drug x time of day within photoperiod, F(12, 180.96) = 2.05, p = 0.022), but there were no pair-wise differences at any time point on either the drug or recovery days and vehicle. Right panel: No effect of AM3506 treatment on wake gamma power. E, NREM epochs. Left panel: For NREM delta power, there was an overall interaction (drug x time of day within photoperiod, F(12, 178.50) = 3.88, p < 0.001) with a main effect of photoperiod (F(1, 115.69) = 6.88, p = 0.010). On the recovery day, NREM delta was elevated at one point during the dark photoperiod (ZT 15–18: t(151.37) = 2.55, p = 0.024). Middle panel: No effect of AM3506 on NREM theta. Right panel: For NREM gamma power, there was a secondary interaction (drug x photoperiod, F(2, 185.23) = 3.54, p = 0.031) and a nested interaction (time of day within photoperiod, F(6, 180.37) = 8.35, p < 0.001) with a main effect of drug treatment (F(2, 171.77) = 9.94, p < 0.001). Overall, AM3506 reduced NREM gamma (t(155.32) = -3.45, p = 0.001), and relative to vehicle, AM3506 specifically reduced NREM gamma across the [file pone.0152473.s008.pdf]

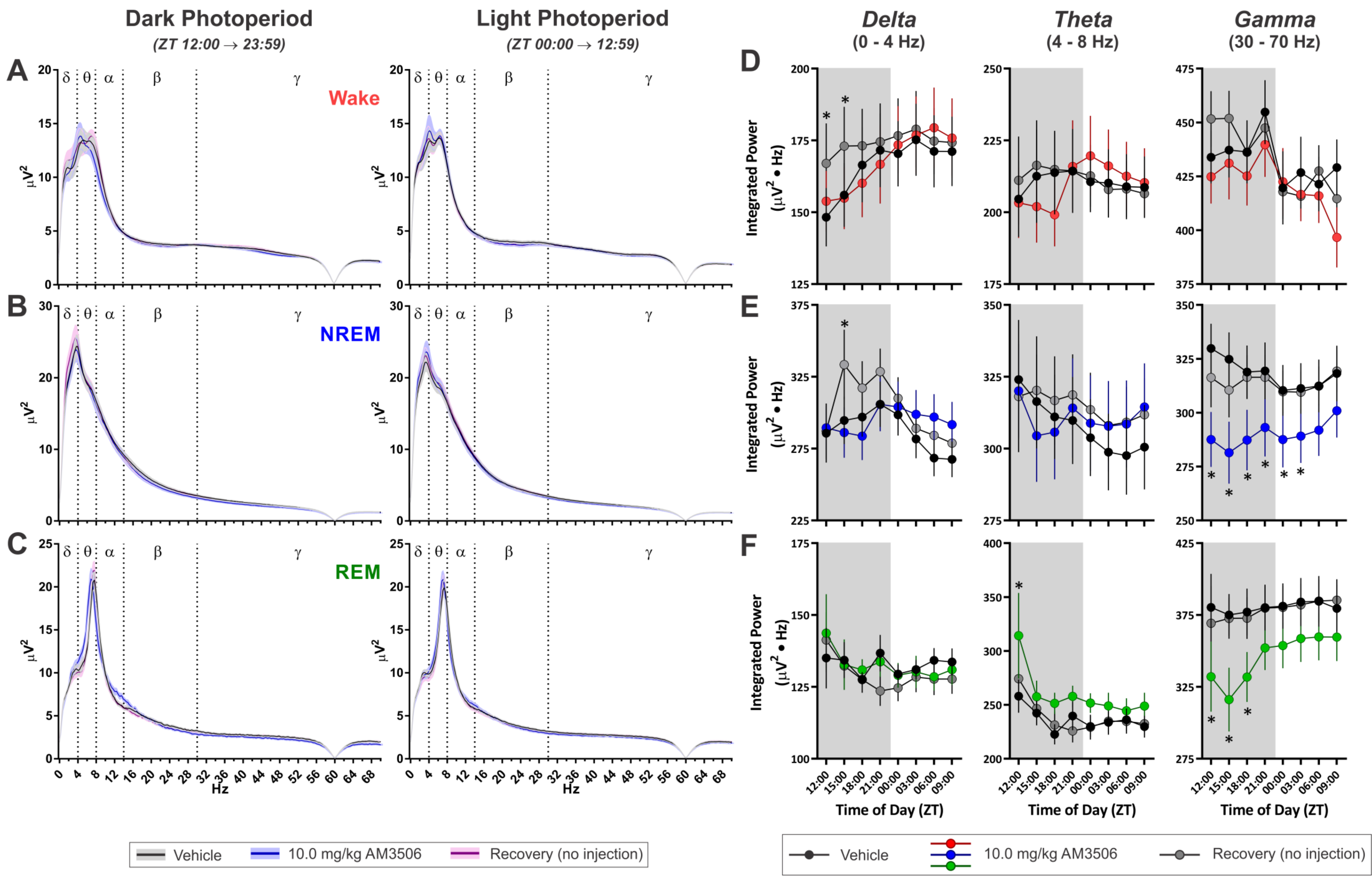

Dark Photoperiod
(ZT 12:00 → 23:59)
Light Photoperiod
(ZT 00:00 → 12:59)
Delta
(0 - 4 Hz)
Theta
(4 - 8 Hz)
Gamma
(30 - 70 Hz)
A
B
C
D
E
F
Wake
NREM
REM
δ θ α β γ
μV2
Hz
Integrated Power
(μV2 • Hz)
Time of Day (ZT)
Vehicle
10.0 mg/kg AM3506
Recovery (no injection)
